# Comparison of the efficacy of supraglottic airway devices in low-risk adult patients: a network meta-analysis and systematic review

**DOI:** 10.1038/s41598-021-94114-7

**Published:** 2021-07-23

**Authors:** Chih-Jun Lai, Yi-Chun Yeh, Yu-Kang Tu, Ya-Jung Cheng, Chih-Min Liu, Shou-Zen Fan

**Affiliations:** 1 Institute of Epidemiology and Preventive Medicine, College of Public Health, National Taiwan University, No. 17, Xu-Zhou Road, Taipei, 100 Taiwan; 2 Department of Anesthesiology, National Taiwan University Hospital, No 7 Zhung Shan S. Road, Taipei, Taiwan; 3 Department of Medical Research, National Taiwan University Hospital, Taipei, Taiwan; 4 Department of Dentistry, National Taiwan University Hospital and School of Dentistry, National Taiwan University, Taipei, Taiwan; 5 Department of Anesthesiology, National Taiwan University Cancer Center, Taipei, Taiwan; 6 Department of Anesthesiology, College of Medicine, National Taiwan University, Taipei, Taiwan

**Keywords:** Health care, Medical research

## Abstract

Numerous supraglottic airway device (SADs) have been designed for adults; however, their relative efficacy, indicated by parameters such as adequacy of sealing, ease of application, and postinsertion complications, remains unclear. We conducted a systematic review and network meta-analysis to evaluate the efficacy of various SADs. We searched electronic databases for randomized controlled trials comparing at least two types of SADs published before December 2019. The primary outcomes were oropharyngeal leak pressure (OLP), risk of first-attempt insertion failure, and postoperative sore throat rate (POST). We included 108 studies (n = 10,645) comparing 17 types of SAD. The Proseal laryngeal mask airway (LMA), the I-gel supraglottic airway, the Supreme LMA, the Streamlined Liner of the Pharynx Airway, the SoftSeal, the Cobra Perilaryngeal Airway, the Air-Q, the Laryngeal Tube, the Laryngeal Tube Suction II, the Laryngeal Tube Suction Disposable, AuraGain, and Protector had significantly higher OLP (mean difference ranging from 3.98 to 9.18 cmH_2_O) compared with that of a classic LMA (C-LMA). The Protector exhibited the highest OLP and was ranked first. All SADs had a similar likelihood of first-attempt insertion failure and POST compared with the C-LMA. Our findings indicate that the Protector may be the best SAD because it has the highest OLP.

Systematic review registration PROSPERO: CRD42017065273.

## Introduction

Supraglottic airway devices (SADs) have been widely used as alternative to tracheal intubation because they can be inserted conveniently and without tracheal intubation associated complications^1^. Since the introduction of the classic laryngeal mask airway (C-LMA)^2^, at least 10 types of SADs with novel materials and designs have been developed^3^. These SADs may have better efficacy than the C-LMA if they have a higher oropharyngeal leak pressure (OLP) and a lower risk of first-attempt insertion failure or airway complications^2^.

Randomized trials have compared only some of the existing SAD types. Moreover, few studies have directly compared all types of SADs, and those that did included a small number of patients. Conducting a trial to compare all types of SADs is exorbitant and impractical. Traditional meta-analyses on some SADs have been conducted, but they tended to pool different types of SADs into a single group, thus precluding the assessment of their clinical performance individually^4–6^. Therefore, which SAD has the best efficacy remains unclear.

A network meta-analysis incorporates direct and indirect evidence into one statistical framework to compare multiple treatments simultaneously, yielding consistent estimates of relative treatment efficacy^7–9^. We therefore conducted a systematic review and network meta-analysis to evaluate the efficacy of SADs in terms of OLP, the risk of first-attempt insertion failure, postoperative sore throat rate (POST) and other efficacy-associated outcomes, including overall insertion failure rate during induction, poor function after successful insertion, SAD failure during maintenance, hypoxia and aspiration.

## Results

**Figure 1:**
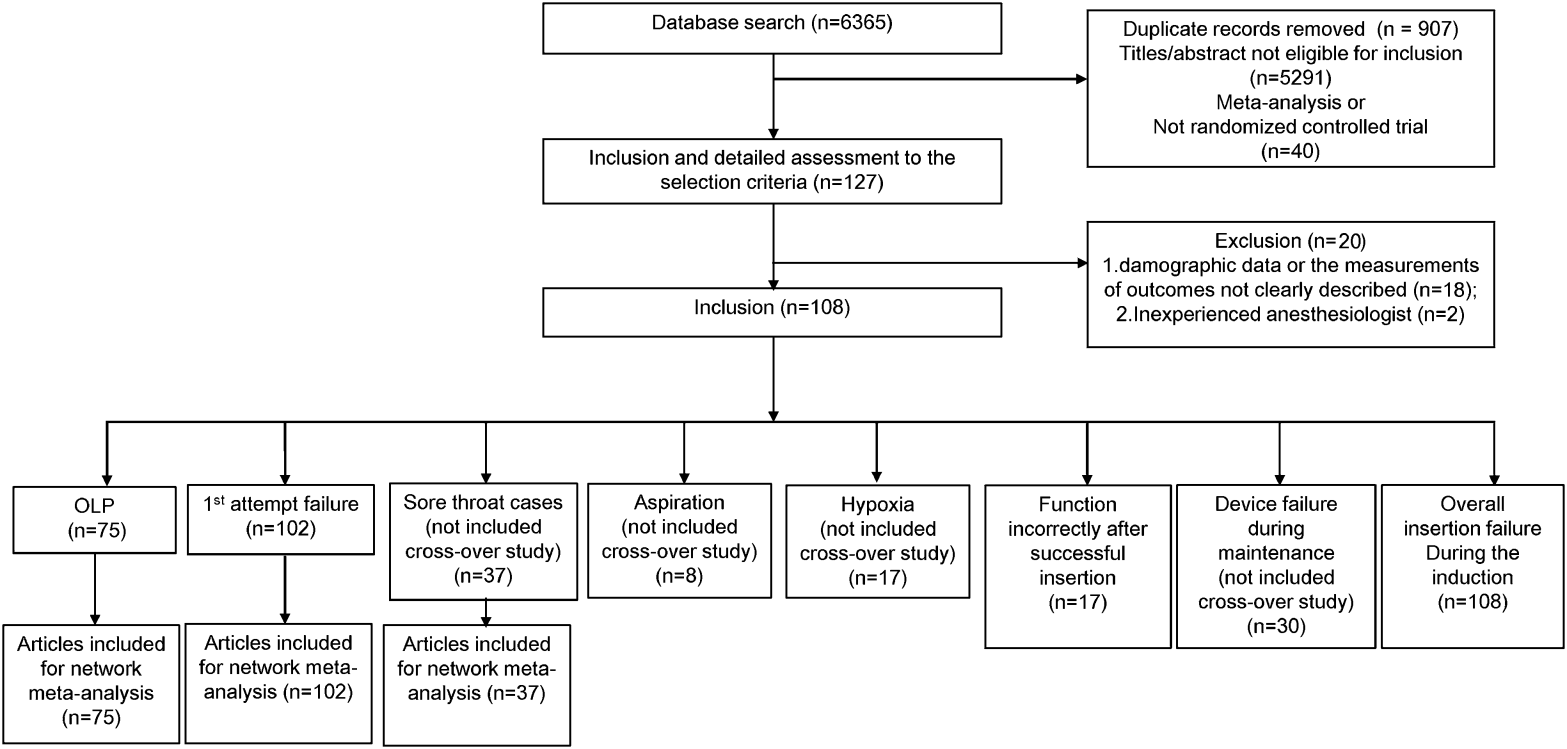
Summary of trial identification and selection. OLP, oropharyngeal leak pressure.

**Figure 2:**
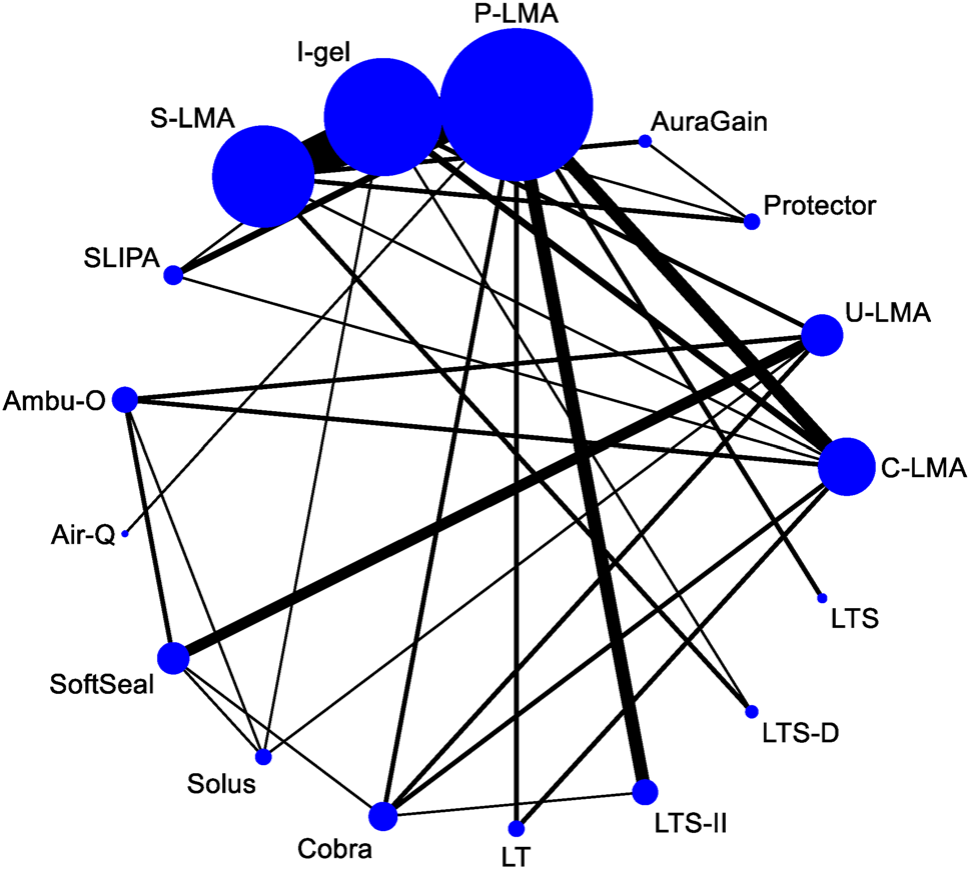
Network of eligible supraglottic airway device comparisons for oropharyngeal leak pressure. The line indicates the number of trials and the dots indicate the number of patients. C-LMA, LMA Classic; U-LMA, Unique LMA; Protector, LMA Protector Airway; AuraGain, Ambu AuraGain Disposable Laryngeal Mask; P-LMA, Proseal LMA; I-gel, I-gel supraglottic airway; S-LMA, Supreme LMA; SLIPA, Streamlined Liner of the Pharynx Airway; Ambu-O, Ambu AuraOnce; Air-Q, Air-Q Masked Laryngeal Airway; SoftSeal, The Portex Soft Seal Laryngeal Mask; Solus, Solus Standard Laryngeal Mask Airway; Cobra, Cobra Perilaryngeal Airway; LT, Laryngeal Tube; LTSII, Laryngeal Tube Suction II; LTS-D, Laryngeal Tube Disposable; LTS, Laryngeal Tube Sonda.

**Figure 3:**
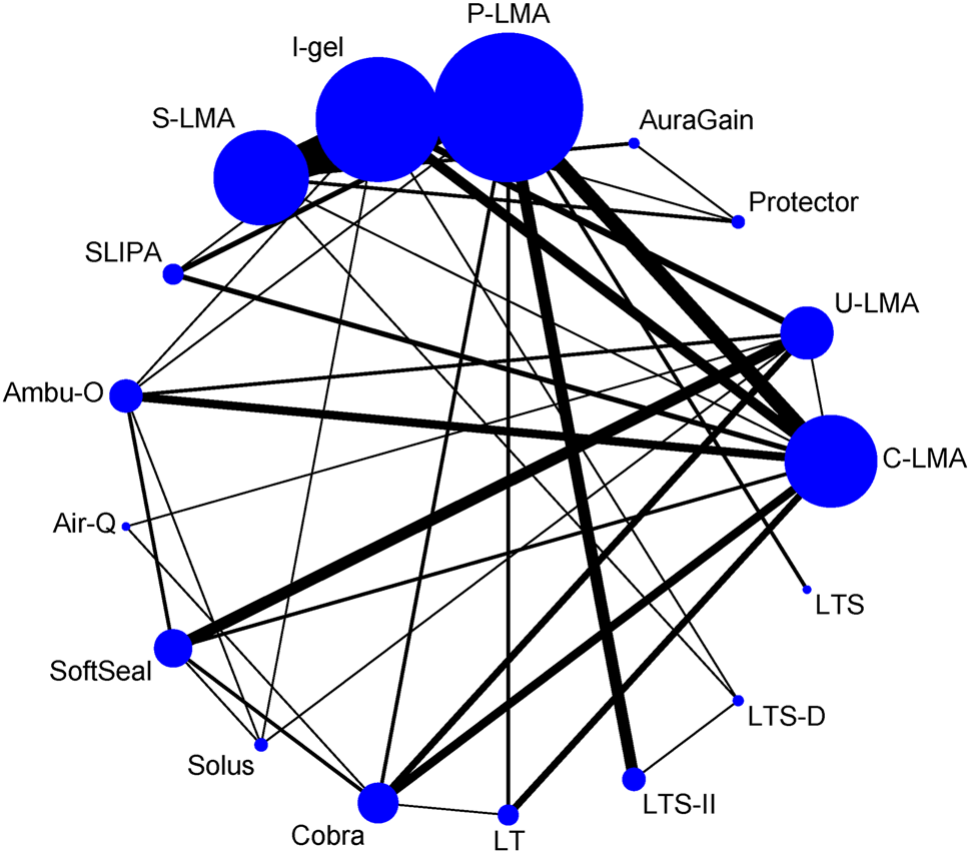
Network of eligible supraglottic airway device comparisons for the risk of first-attempt insertion failure. The line indicates the number of trials and the dots indicate the number of patients. C-LMA, LMA Classic; U-LMA, Unique LMA; Protector, LMA Protector Airway; AuraGain, Ambu AuraGain Disposable Laryngeal Mask; P-LMA, Proseal LMA; I-gel, I-gel supraglottic airway; S-LMA, Supreme LMA; SLIPA, Streamlined Liner of the Pharynx Airway; Ambu-O, Ambu AuraOnce; Air-Q, Air-Q Masked Laryngeal Airway; SoftSeal, The Portex Soft Seal Laryngeal Mask; Solus, Solus Standard Laryngeal Mask Airway; Cobra, Cobra Perilaryngeal Airway; LT, Laryngeal Tube; LTSII, Laryngeal Tube Suction II; LTS-D, Laryngeal Tube Disposable; LTS, Laryngeal Tube Sonda.

**Figure 4:**
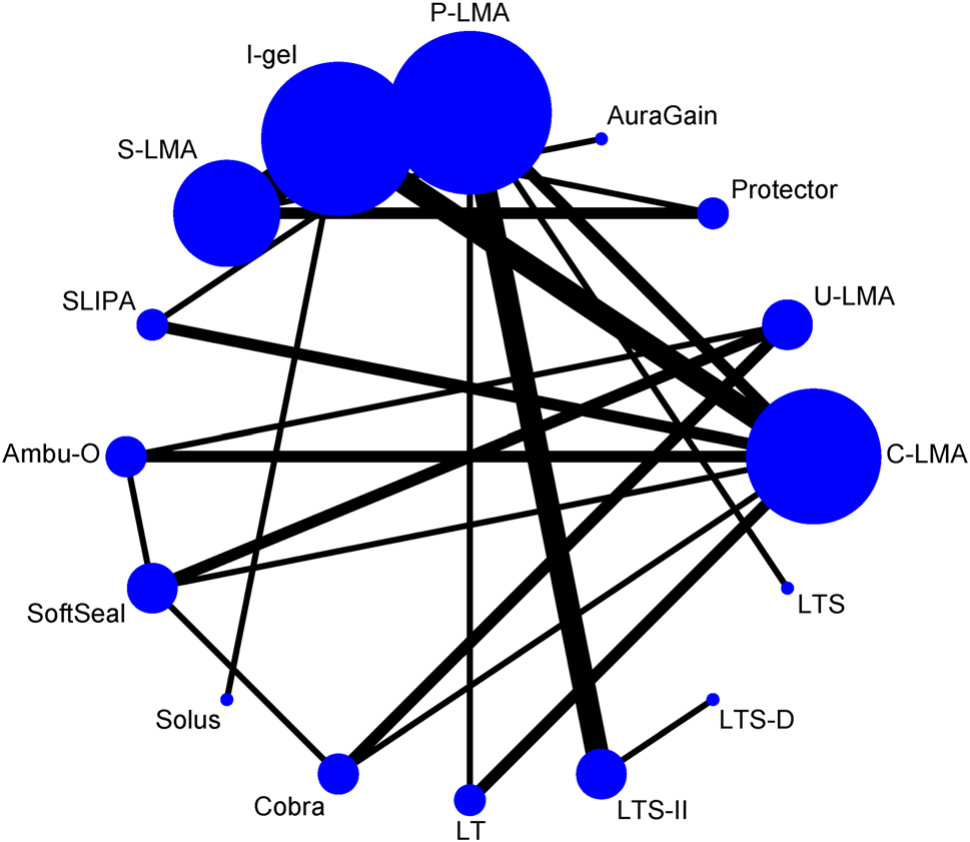
Network of eligible supraglottic airway device comparisons for postoperative sore throat rate. The line indicates the number of trials and the dots indicate the number of patients. C-LMA, LMA Classic; U-LMA, Unique LMA; Protector, LMA Protector Airway; AuraGain, Ambu AuraGain Disposable Laryngeal Mask; P-LMA, Proseal LMA; I-gel, I-gel supraglottic airway; S-LMA, Supreme LMA; SLIPA, Streamlined Liner of the Pharynx Airway; Ambu-O, Ambu AuraOnce; Air-Q, Air-Q Masked Laryngeal Airway; SoftSeal, The Portex Soft Seal Laryngeal Mask; Solus, Solus Standard Laryngeal Mask Airway; Cobra, Cobra Perilaryngeal Airway; LT, Laryngeal Tube; LTSII, Laryngeal Tube Suction II; LTS-D, Laryngeal Tube Disposable; LTS, Laryngeal Tube Sonda.

Figure 1 presents a flow chart of the trial selection process in 108 eligible randomized controlled trials met our inclusion criteria. Figures 2, 3 and 4 illustrate the network of included studies on the OLP, risk of first-attempt insertion failure, and POST. Table 1 summarizes the characteristics of the 17 types of SADs included in this study, and Supplementary Table S1 summarizes the clinical and methodological characteristics and the main outcomes of each trial.

**Table 1:** The characteristics of supraglottic airway devices.

Supraglottic airway device	Reusable (R)/ Disposable (D)	Material	1st or 2nd generation device	Oesophageal channel	Unique features in the design
C-LMA	R	Silicone	1st	–	The original laryngeal mask airway with aperture bars
U-LMA	D	PVC	1st	–	The features similar to the C-LMA made of disposable material
Protector	D	PVC	2nd	✓	The only laryngeal mask that combines a pharyngeal chamber and dual gastric drainage channels
AuraGain	D	PVC	2nd	✓	AuraGain has anatomically curved SGA with integrated gastric access and intubation capability
P-LMA	R	Silicone	2nd	✓	No aperture bars, reinforced airway tube, deeper cuff bowl with posterior cuff, bite block, introducer
I-gel	D	SEBS	2nd	✓	No aperture bars, on-inflatable cuff, bite block, buccal stabilizer
S-LMA	D	PVC	2nd	✓	No aperture bars, performed semi-rigid airway tube, large inflatable plastic cuff without posterior cuff, bite block, fins in the mask bowl
SLIPA	D	EVA	2nd	–	large capacity of the storing chamber of regurgitated gastric fluid, made of thermoplastic material (polyethylene and vinyl acetate), non-inflatable cuff
Ambu-O	D	PVC	1st	–	Performed curved shaft of airway tube, no aperture bar
Air-Q	D	PVC	1st	–	No pilot balloon, self-pressurized
SoftSeal	D	PVC	1st	–	No aperture bar, elliptical cuff, large cuff bowl
Solus	D	PVC	1st	–	No aperture bar, pliable airway tube, smooth-surfaced back plate
Cobra	D	PVC	1st	–	Tip like cobra head, elliptical cuff
LT	R	Silicone	1st		consists of an airway tube with a small cuff attached at the tip (distal cuff) and a larger balloon cuff at the middle part of the tube (proximal cuff)
LTSII	R	Silicone	2nd	✓	LTSII is shorter and softer than the LT
LTS-D	D	PVC	2nd	✓	The feature is similar to the LTSII made of disposable material
LTS	R	Silicone	2nd	✓	LTS is shorter than the LTSII, and distal cuff without surface protruding is different from the LTSII

PVC: polyvinyl chloride; SEBS, Styrene Ethylene Butylene Styrene; EVA, ethylene vinyl-acetate copolymer; C-LMA, LMA Classic; U-LMA, Unique LMA; Protector, LMA Protector Airway; AuraGain, Ambu AuraGain Disposable Laryngeal Mask; P-LMA, Proseal LMA; I-gel, I-gel supraglottic airway; S-LMA, Supreme LMA; SLIPA, Streamlined Liner of the Pharynx Airway; Ambu-O, Ambu AuraOnce; Air-Q, Air-Q Masked Laryngeal Airway.; SoftSeal, The Portex Soft Seal Laryngeal Mask; Solus, Solus Standard Laryngeal Mask Airway; Cobra, Cobra Perilaryngeal Airway; LT, Laryngeal Tube; LTSII, Laryngeal Tube Suction II; LTS-D, Laryngeal Tube Disposable; LTS, Laryngeal Tube Sonda;

### Oropharyngeal leak pressure (OLP)

OLP data were obtained of 7784 adult patients in 75 trials, including 17 types of SADs. The pooled estimates of differences in OLP in the network meta-analysis are displayed in the upper triangle of Table 2, whereas the lower triangle of Table 2 presented the results of traditional pairwise meta-analyses. The Protector laryngeal mask airway (Protector) and Ambu AuraGain Disposable Laryngeal Mask (AuraGain) achieved the greatest highest and most improved OLP compared with that of the C-LMA, with mean differences (95% confidence interval) of 9.18 (5.60, 12.75) and 7.65 (3.63,11.67) cmH_2_O, respectively. The Proseal laryngeal mask airway (P-LMA), I-gel Supraglottic Airway (I-gel), Supreme laryngeal mask airway (S-LMA) and Streamlined Liner of the Pharynx Airway (SLIPA) had significantly higher OLP than that of the C-LMA, with mean differences ranging from 3.98 to 6.72 cmH_2_O. The Portex Soft Seal Laryngeal Mask (SoftSeal), Air-Q Masked Laryngeal Airway (Air-Q), and Cobra Perilaryngeal Airway (Cobra) also achieved higher OLP than did the C-LMA, with mean differences ranging from 4.55 to 6.72 cmH_2_O. In the laryngeal tube category, the Laryngeal Tube (LT) and the Laryngeal Tube Suction II (LTS-II), but not the Laryngeal Tube Disposable (LTS-D) or Laryngeal Tube Sonda (LTS), exhibited significantly greater OLP (5.94 to 7.25 cmH_2_O higher) than that of the C-LMA. The Unique laryngeal mask airway (U-LMA), Solus Standard Laryngeal Mask Airway (Solus), Ambu AuraOnce (Ambu-O), LTS-D and LTS exhibited non-significantly higher OLP than that of C-LMA, with mean differences ranging from − 0.46 to 4.16 cmH_2_O. The Protector had the highest surface under the cumulative ranking curve (SUCRA) values among the SADs (Supplementary Table S2), whereas the U-LMA and C-LMA had the lowest SUCRA values.

**Table 2:** Results for oropharyngeal leak pressure from network meta-analysis.

C-LMA	− 0.46 (− 3.39,2.47)	**9.18** **(5.60,12.75)**	**7.65** **(3.63,11.67)**	**6.72** **(5.05,8.40)**	**3.98** **(2.14,5.82)**	**4.96** **(2.96,6.95)**	**5.39** **(2.41,8.37)**	2.50 (− 0.70,5.71)	**6.72** **(0.49,12.95)**	**4.55** **(1.13,7.96)**	1.41 (− 2.87,5.69)	**5.28** **(2.66,7.90)**	**5.94** **(2.75,9.13)**	**7.25** **(4.40,10.10)**	4.16 (− 0.08,8.39)	4.03 (− 0.77,8.83)
	U− LMA	**9.63** **(5.44,13.83)**	**8.11** **(3.51,12.70)**	**7.18** **(4.33,10.03)**	**4.44** **(1.66,7.23)**	**5.41** **(2.42,8.41)**	**5.85** **(2.02,9.69)**	2.96 (− 0.27,6.19)	**7.18** **(0.54,13.82)**	**5.00** **(2.47,7.54)**	1.87 (− 2.29,6.03)	**5.73** **(2.80,8.66)**	**6.40** **(2.26,10.54)**	**7.71** **(4.10,11.32)**	4.61 (− 0.15,9.38)	4.49 (− 0.84,9.82)
		Protector	− 1.53 (− 5.51,2.45)	− 2.45 (− 5.75,0.84)	− **5.19** **(**− **8.41,**− **1.98)**	− **4.22** **(**− **7.29,**− **1.15)**	− 3.78 (− 7.99,0.43)	− **6.67** **(**− **11.24,**− **2.10)**	− 2.45 (− 9.30,4.39)	− **4.63** **(**− **9.22,**− **0.04)**	− **7.76** **(**− **12.95,**− **2.58)**	− 3.90 (− 7.98,0.18)	− 3.24 (− 7.78,1.31)	− 1.93 (− 5.97,2.12)	− **5.02** **(**− **9.89,**− **0.15)**	− 5.15 (− 10.72,0.43)
		− 1.90 (− 4.48,0.68)	AuraGain	− 0.93 (− 4.69,2.84)	− 3.66 (− 7.40,0.07)	− 2.69 (− 6.21,0.83)	− 2.26 (− 6.85,2.34)	− **5.15** **(**− **10.08,**− **0.21)**	− 0.93 (− 8.01,6.16)	− 3.10 (− 8.05,1.85)	− **6.24** **(**− **11.76,**− **0.72)**	− 2.37 (− 6.86,2.11)	− 1.71 (− 6.61,3.19)	− 0.40 (− 4.84,4.04)	− 3.49 (− 8.68,1.69)	− 3.62 (− 9.49,2.25)
**7.65** **(7.18,8.12)**				P− LMA	− **2.74** **(**− **4.02,**− **1.46)**	− **1.77** **(**− **3.19,**− **0.35)**	− 1.33 (− 4.08,1.42)	− **4.22** **(**− **7.57,**− **0.87)**	− 0.00 (− 6.00,6.00)	− 2.18 (− 5.57,1.21)	− **5.31** **(**− **9.52,**− **1.10)**	− 1.45 (− 4.01,1.12)	− 0.78 (− 3.98,2.42)	0.53 (− 1.84,2.89)	− 2.57 (− 6.57,1.43)	− 2.69 (− 7.19,1.81)
**3.49** **(2.01,4.97)**	**10.03** **(9.45,10.61)**	− **4.00** **(**− **6.44,**− **1.56)**		− **2.90** **(**− **3.37,**− **2.42)**	i− gel	0.97 (− 0.40,2.34)	1.41 (− 1.45,4.27)	− 1.48 (− 4.83,1.87)	2.74 (− 3.40,8.87)	0.56 (− 2.79,3.92)	− 2.57 (− 6.69,1.54)	1.29 (− 1.39,3.98)	1.96 (− 1.41,5.32)	**3.26** **(0.59,5.93)**	0.17 (− 3.76,4.10)	0.05 (− 4.63,4.73)
**8.50** **(7.26,9.74)**		− **4.27** **(**− **5.39,**− **3.16)**	− **4.35** **(**− **5.25,**− **3.45)**	− **1.11** **(**− **1.65,**− **0.58)**	**0.57** **(0.04,1.10)**	S− LMA	0.44 (− 2.56,3.43)	− 2.45 (− 5.95,1.04)	1.77 (− 4.40,7.93)	− 0.41 (− 3.93,3.11)	− 3.54 (− 7.83,0.74)	0.32 (− 2.50,3.14)	0.99 (− 2.45,4.42)	2.29 (− 0.45,5.04)	− 0.80 (− 4.62,3.02)	− 0.93 (− 5.65,3.79)
0.00 (− 1.82,1.82)				0.20 (− 1.15,1.55)	**2.84** **(1.35,4.33)**		SLIPA	− 2.89 (− 7.08,1.30)	1.33 (− 5.27,7.93)	− 0.85 (− 5.09,3.40)	− 3.98 (− 8.90,0.94)	− 0.12 (− 3.77,3.54)	0.55 (− 3.58,4.67)	1.85 (− 1.76,5.47)	− 1.24 (− 6.02,3.54)	− 1.36 (− 6.64,3.91)
1.63 (− 0.26,3.51)	**3.87** **(1.98,5.75)**							Ambu− O	4.22 (− 2.65,11.09)	2.05 (− 1.42,5.51)	− 1.09 (− 5.52,3.34)	2.77 (− 0.88,6.43)	3.44 (− 0.98,7.86)	**4.75** **(0.71,8.79)**	1.65 (− 3.45,6.75)	1.53 (− 4.08,7.14)
				0.00 (− 2.55,2.55)					Air− Q	− 2.18 (− 9.07,4.72)	− 5.31 (− 12.64,2.02)	− 1.45 (− 7.97,5.08)	− 0.78 (− 7.58,6.02)	0.53 (− 5.92,6.98)	− 2.57 (− 9.78,4.64)	− 2.69 (− 10.19,4.81)
	**3.12** **(2.39,3.86)**							**2.66** **(0.65,4.66)**		SoftSeal	− 3.13 (− 7.48,1.21)	0.73 (− 2.67,4.13)	1.39 (− 3.12,5.91)	2.70 (− 1.34,6.74)	− 0.39 (− 5.51,4.72)	− 0.52 (− 6.15,5.12)
	− 1.35 (− 4.12,1.42)				**3.30** **(0.60,6.01)**			− **4.08** **(**− **6.55,**− **1.61)**		− **8.16** **(**− **10.68,**− **5.64)**	Solus	3.86 (− 0.65,8.38)	4.53 (− 0.65,9.71)	**5.84** **(1.06,10.62)**	2.74 (− 2.92,8.40)	2.62 (− 3.54,8.78)
**4.42** **(2.85,5.99)**	**5.29** **(3.91,6.66)**			− 0.20 (− 1.40,1.00)						− 1.00 (− 2.50,0.50)		Cobra	0.66 (− 3.28,4.60)	1.97 (− 1.29,5.24)	− 1.12 (− 5.79,3.55)	− 1.25 (− 6.43,3.93)
**7.26** **(5.30,9.22)**				− 2.10 (− 4.20,0.00)									LT	1.31 (− 2.65,5.27)	− 1.79 (− 6.86,3.29)	− 1.91 (− 7.43,3.61)
				**1.17** **(0.20,2.14)**								1.50 (− 0.37,3.37)		LTS− II	− 3.09 (− 7.73,1.54)	− 3.22 (− 8.30,1.87)
					− 1.90 (− 4.23,0.43)	− 0.11 (− 1.54,1.31)									LTS− D	− 0.13 (− 6.15,5.89)
				− **2.41** **(**− **4.68,**− **0.15)**												LTS

The device in column one is the index device and that in row one is the comparator. The upper triangle is the results of the network meta-analysis, and the lower triangle is the results of the traditional pairwise meta-analysis. The device in the row one minus the device in column one equals the mean difference, which unit is cmH_2_O. Bold and underscored means significance. The significance means the confidence interval not containing the “0”. C-LMA, LMA Classic; U-LMA, Unique LMA; Protector, LMA Protector Airway; AuraGain, Ambu AuraGain Disposable Laryngeal Mask; P-LMA, Proseal LMA; I-gel, I-gel supraglottic airway; S-LMA, Supreme LMA; SLIPA, Streamlined Liner of the Pharynx Airway; Ambu-O, Ambu AuraOnce; Air-Q, Air-Q Masked Laryngeal Airway.; SoftSeal, The Portex Soft Seal Laryngeal Mask; Solus, Solus Standard Laryngeal Mask Airway; Cobra, Cobra Perilaryngeal Airway; LT, Laryngeal Tube; LTSII, Laryngeal Tube Suction II; LTS-D, Laryngeal Tube Disposable; LTS, Laryngeal Tube Sonda.

### First-attempt insertion failure

Data on the first-attempt insertion failure were obtained for 10,191 adult patients in 102 trials with 17 types of SAD. The results of the network meta-analysis are presented in the upper triangle of in Table 3. The lower triangle in Table 3 presents the results of traditional pairwise meta-analyses. The risk of first-attempt insertion failure of each of the SADs was comparable to that of the C-LMA. The S-LMA and Ambu-O had significantly lower risks of first-attempt insertion failure than that of the P-LMA. The S-LMA also achieved a significantly lower risk than that of the I-gel. The P-LMA, Cobra and LTS achieved significantly higher risks than that of the U-LMA. The Cobra and LTS demonstrated significantly higher risks than that of the S-LMA. In terms of SUCRA values, the Ambu-O and S-LMA were ranked the highest, the AuraGain was ranked fifth and the Protector, was ranked seventh (Supplementary Table S4).

**Table 3:** Results for the risk of first-attempt insertion failure from network meta-analysis and pairwise meta-analysis.

C-LMA	0.74 (0.44,1.24)	0.90 (0.38,2.11)	0.71 (0.29,1.79)	1.34 (0.96,1.86)	0.99 (0.70,1.40)	0.69 (0.45,1.04)	1.29 (0.67,2.51)	0.67 (0.40,1.13)	0.56 (0.11,2.94)	1.06 (0.60,1.88)	1.22 (0.59,2.55)	1.37 (0.77,2.42)	0.85 (0.52,1.38)	1.16 (0.64,2.10)	1.40 (0.56,3.50)	2.40 (0.88,6.53)
1.49 (0.46,4.84)	U-LMA	1.21 (0.47,3.11)	0.96 (0.35,2.64)	**1.80** **(1.05,3.11)**	1.33 (0.80,2.23)	0.93 (0.52,1.66)	1.75 (0.77,3.96)	0.90 (0.49,1.64)	0.75 (0.15,3.81)	1.43 (0.89,2.30)	1.65 (0.80,3.42)	**1.84** **(1.03,3.30)**	1.14 (0.57,2.28)	1.57 (0.76,3.26)	1.89 (0.70,5.13)	**3.24** **(1.09,9.65)**
		Protector	0.80 (0.27,2.30)	1.49 (0.65,3.38)	1.10 (0.49,2.47)	0.77 (0.35,1.66)	1.44 (0.51,4.08)	0.74 (0.28,1.96)	0.62 (0.10,3.93)	1.18 (0.44,3.16)	1.36 (0.47,3.98)	1.52 (0.56,4.11)	0.94 (0.36,2.44)	1.29 (0.50,3.36)	1.56 (0.49,4.94)	2.67 (0.76,9.36)
		**0.08** **(0.01,0.57)**	AuraGain	1.87 (0.77,4.54)	1.38 (0.57,3.34)	0.96 (0.42,2.19)	1.81 (0.60,5.44)	0.93 (0.33,2.61)	0.78 (0.12,5.10)	1.49 (0.52,4.22)	1.71 (0.56,5.27)	1.91 (0.67,5.44)	1.18 (0.43,3.27)	1.63 (0.59,4.48)	1.96 (0.60,6.47)	3.36 (0.92,12.28)
**1.68** **(1.28,2.22)**				P-LMA	0.74 (0.55,1.00)	**0.51** **(0.37,0.72)**	0.97 (0.49,1.90)	**0.50** **(0.28,0.89)**	0.42 (0.08,2.22)	0.79 (0.43,1.46)	0.92 (0.43,1.93)	1.02 (0.56,1.87)	0.63 (0.37,1.07)	0.87 (0.53,1.42)	1.05 (0.43,2.55)	1.80 (0.70,4.63)
**1.47** **(1.01,2.14)**	1.21 (0.64,2.29)	0.80 (0.23,2.82)		**0.71** **(0.53,0.96)**	I-gel	**0.70** **(0.50,0.96)**	1.31 (0.65,2.64)	0.68 (0.38,1.20)	0.56 (0.11,2.99)	1.07 (0.60,1.94)	1.24 (0.61,2.53)	1.38 (0.75,2.54)	0.86 (0.49,1.50)	1.18 (0.66,2.09)	1.42 (0.60,3.37)	2.43 (0.90,6.55)
0.50 (0.17,1.51)		1.48 (0.87,2.52)	0.68 (0.36,1.28)	**0.45** **(0.32,0.64)**	**0.81** **(0.60,1.08)**	S-LMA	1.88 (0.90,3.91)	0.97 (0.52,1.81)	0.81 (0.15,4.39)	1.54 (0.81,2.94)	1.78 (0.83,3.85)	**1.99** **(1.03,3.81)**	1.23 (0.68,2.24)	1.69 (0.93,3.06)	2.04 (0.86,4.85)	**3.49** **(1.28,9.53)**
0.78 (0.40,1.52)				2.24 (0.94,5.35)	0.11 (0.01,2.00)		SLIPA	0.52 (0.22,1.19)	0.43 (0.07,2.56)	0.82 (0.35,1.94)	0.95 (0.36,2.49)	1.06 (0.45,2.49)	0.65 (0.29,1.46)	0.90 (0.39,2.07)	1.09 (0.36,3.25)	1.86 (0.58,5.93)
0.71 (0.42,1.18)	1.07 (0.57,2.02)			0.50 (0.13,1.86)	0.43 (0.12,1.54)			Ambu-O	0.84 (0.15,4.58)	1.59 (0.85,2.98)	1.83 (0.85,3.95)	**2.04** **(1.01,4.15)**	1.27 (0.62,2.57)	1.74 (0.82,3.71)	2.10 (0.76,5.84)	**3.59** **(1.19,10.88)**
	0.49 (0.10,2.26)								Air-Q	1.90 (0.36,10.10)	2.19 (0.38,12.70)	2.45 (0.48,12.45)	1.52 (0.27,8.50)	2.08 (0.36,11.90)	2.52 (0.39,16.36)	4.30 (0.63,29.38)
1.56 (0.65,3.79)	**1.49** **(1.06,2.10)**							1.39 (0.79,2.45)		SoftSeal	1.15 (0.55,2.42)	1.29 (0.67,2.46)	0.80 (0.38,1.66)	1.09 (0.50,2.38)	1.32 (0.47,3.72)	2.26 (0.73,6.95)
	**2.30** **(1.22,4.32)**				0.86 (0.43,1.70)			**2.09** **(1.15,3.82)**		1.35 (0.83,2.21)	Solus	1.12 (0.48,2.59)	0.69 (0.29,1.64)	0.95 (0.39,2.32)	1.15 (0.38,3.50)	1.96 (0.59,6.54)
1.22 (0.63,2.37)	1.50 (0.83,2.71)			1.93 (0.61,6.13)					1.50 (0.31,7.30)	1.98 (0.85,4.58)		Cobra	0.62 (0.30,1.29)	0.85 (0.39,1.85)	1.03 (0.36,2.91)	1.76 (0.57,5.40)
**0.48** **(0.33,0.71)**				1.52 (0.88,2.63)								**9.00** **(1.21,66.70)**	LT	1.38 (0.67,2.82)	1.66 (0.60,4.57)	2.84 (0.96,8.38)
				0.90 (0.66,1.21)										LTS-II	1.21 (0.45,3.25)	2.06 (0.71,6.00)
					1.81 (0.97,3.37)	1.68 (0.93,3.06)								1.00 (0.07,15.26)	LTS-D	1.71 (0.47,6.25)
				1.84 (0.89,3.80)												LTS

The device in column one is the index device and that in row one is the comparator. The upper triangle is the results of the network meta-analysis, and the lower triangle is the results of the traditional pairwise meta-analysis. The device in column one is divided by the device in the row one equals the risk ratio, which did not have unit. Bold and underscored means significance. The significance means the confidence interval not containing the “1”. C-LMA, LMA Classic; U-LMA, Unique LMA; Protector, LMA Protector Airway; AuraGain, Ambu AuraGain Disposable Laryngeal Mask; P-LMA, Proseal LMA; I-gel, I-gel supraglottic airway; S-LMA, Supreme LMA; SLIPA, Streamlined Liner of the Pharynx Airway; Ambu-O, Ambu AuraOnce; Air-Q, Air-Q Masked Laryngeal Airway.; SoftSeal, The Portex Soft Seal Laryngeal Mask; Solus, Solus Standard laryngeal mask airway; Cobra, Cobra Perilaryngeal airway; LT. Laryngeal Tube; LTSII, Laryngeal Tube Suction II; LTS-D, Laryngeal Tube Disposable; LTS, Laryngeal Tube Sonda.

### Postoperative sore throat rate (POST) within 24 h and other secondary efficacy-related outcomes

Data on POST were obtained for 4125 adult patients in 37 trials, with 16 types of SADs. POST-related data on the Air-Q were unavailable. POST ranged from 0 to 50%. The results of the network meta-analysis are presented in the upper triangle of Table 4; none of the SAD exhibited a significant difference compared with the C-LMA. The S-LMA and Cobra had significantly higher POSTs than that of the AuraGain. The Cobra also attained a significantly higher POST than that of the Ambu-O. The lower triangle of Table 4 presents the results of the traditional pairwise analysis. The SADs with the highest ranking and lowest POST were the AuraGain and Cobra (Supplementary Table S7). The results regarding the overall insertion failure rate during induction, poor function after successful insertion, device failure during maintenance, hypoxia, and aspiration are presented in Supplementary Tables S10 to S14. Because the evidence on these outcomes is limited and includes many zero events, a network meta-analysis was considered infeasible.

**Table 4:** Results for postoperative sore throat rate from network meta-analysis and pairwise meta-analysis.

C-LMA	1.39 (0.43,4.53)	0.92 (0.33,2.55)	0.23 (0.05,1.11)	0.85 (0.46,1.59)	0.58 (0.31,1.08)	0.99 (0.44,2.23)	0.80 (0.31,2.06)	0.39 (0.13,1.12)	1.00 (0.39,2.54)	1.36 (0.19,9.86)	1.72 (0.64,4.57)	1.12 (0.53,2.37)	0.84 (0.32,2.20)	2.59 (0.08,81.40)	2.03 (0.36,11.52)
	U-LMA	0.66 (0.14,3.14)	0.17 (0.02,1.18)	0.61 (0.16,2.32)	0.41 (0.11,1.57)	0.71 (0.17,2.97)	0.58 (0.13,2.60)	0.28 (0.06,1.27)	0.72 (0.25,2.08)	0.98 (0.10,9.78)	1.23 (0.49,3.12)	0.81 (0.20,3.25)	0.60 (0.13,2.75)	1.86 (0.05,71.05)	1.45 (0.18,11.88)
		Protector	0.25 (0.05,1.23)	0.93 (0.35,2.46)	0.63 (0.26,1.51)	1.08 (0.46,2.50)	0.87 (0.24,3.19)	0.42 (0.10,1.85)	1.09 (0.27,4.34)	1.48 (0.19,11.77)	1.87 (0.45,7.68)	1.22 (0.36,4.13)	0.91 (0.27,3.09)	2.82 (0.08,96.00)	2.20 (0.33,14.63)
			AuraGain	3.64 (0.82,16.24)	2.46 (0.55,11.01)	**4.23** **(1.12,15.96)**	3.43 (0.60,19.56)	1.66 (0.25,10.96)	4.27 (0.70,26.17)	5.84 (0.53,64.34)	**7.34** **(1.17,46.17)**	4.80 (0.89,25.91)	3.58 (0.67,18.97)	11.09 (0.27,451.17)	8.67 (0.95,78.67)
**0.44** **(0.25,0.77)**				P-LMA	0.68 (0.35,1.30)	1.16 (0.58,2.32)	0.94 (0.37,2.43)	0.46 (0.13,1.58)	1.17 (0.38,3.59)	1.60 (0.22,11.68)	2.02 (0.63,6.46)	1.32 (0.55,3.16)	0.98 (0.47,2.06)	3.05 (0.10,90.50)	2.38 (0.47,12.05)
0.91 (0.59,1.41)		0.69 (0.35,1.34)		**0.40** **(0.19,0.83)**	I-gel	1.72 (0.86,3.45)	1.40 (0.50,3.92)	0.68 (0.20,2.31)	1.74 (0.56,5.40)	2.37 (0.36,15.46)	2.98 (0.93,9.55)	1.95 (0.77,4.94)	1.45 (0.55,3.87)	4.51 (0.14,142.14)	3.52 (0.61,20.25)
		1.00 (0.46,2.20)	**4.23** **(1.73,10.38)**	0.93 (0.58,1.50)	**2.81** **(1.26,6.26)**	S-LMA	0.81 (0.26,2.50)	0.39 (0.10,1.50)	1.01 (0.29,3.47)	1.38 (0.19,10.18)	1.73 (0.49,6.19)	1.13 (0.40,3.21)	0.85 (0.31,2.32)	2.62 (0.08,83.37)	2.05 (0.35,11.92)
0.44 (0.16,1.21)				1.81 (0.90,3.66)			SLIPA	0.48 (0.12,1.98)	1.24 (0.33,4.75)	1.70 (0.20,14.45)	2.14 (0.55,8.30)	1.40 (0.44,4.45)	1.04 (0.32,3.43)	3.23 (0.10,108.76)	2.52 (0.39,16.51)
**0.45** **(0.23,0.89)**	0.34 (0.01,8.14)							Ambu-O	2.57 (0.66,9.95)	3.51 (0.37,33.04)	**4.42** **(1.09,17.88)**	2.89 (0.79,10.58)	2.15 (0.51,9.04)	6.67 (0.18,245.87)	5.21 (0.68,40.10)
0.75 (0.41,1.38)	0.89 (0.35,2.27)							7.18 (0.38,134.48)	SoftSeal	1.37 (0.15,12.21)	1.72 (0.63,4.69)	1.12 (0.34,3.73)	0.84 (0.22,3.21)	2.59 (0.07,92.28)	2.03 (0.28,14.54)
					2.37 (0.48,11.74)					Solus	1.26 (0.14,11.41)	0.82 (0.10,6.66)	0.61 (0.07,5.08)	1.90 (0.04,96.43)	1.48 (0.11,19.28)
**2.14** **(1.02,4.50)**	1.06 (0.52,2.13)								2.14 (0.77,5.94)		Cobra	0.65 (0.19,2.24)	0.49 (0.12,1.93)	1.51 (0.04,54.39)	1.18 (0.16,8.69)
1.37 (0.84,2.23)				0.71 (0.24,2.13)								LT	0.74 (0.24,2.33)	2.31 (0.07,76.48)	1.81 (0.29,11.38)
				1.11 (0.65,1.87)									LTS-II	3.10 (0.11,84.81)	2.42 (0.41,14.41)
													3.10 (0.13,73.14)	LTS-D	0.78 (0.02,33.53)
				2.38 (0.65,8.68)											LTS

The device in column one is the index device and that in row one is the comparator. The device in the column one is divided by the device in the row one equals risk ratio, which did not have unit. Bold and underscored means significance. The significance means the confidence interval not containing the “1”. C-LMA, LMA Classic; U-LMA, Unique LMA; Protector, LMA Protector Airway; AuraGain, Ambu AuraGain Disposable Laryngeal Mask; P-LMA, Proseal LMA; I-gel, I-gel supraglottic airway; S-LMA, Supreme LMA; SLIPA, Streamlined Liner of the Pharynx Airway; Ambu-O, Ambu AuraOnce; SoftSeal, The Portex Soft Seal Laryngeal Mask; Solus, Solus Standard Laryngeal Mask Airway; Cobra, Cobra Perilaryngeal Airway; LT, Laryngeal Tube; LTS II, Laryngeal Tube Suction II; LTS-D, Laryngeal Tube Disposable; LTS, Laryngeal Tube Sonda.

### Meta-regression of the effect of using the neuromuscular blocking agents on OLP, risk of first-attempt insertion failure and POST, and effect of positive pressure ventilation on POST

Assessment of the transitivity assumption was performed for the use of neuromuscular blocking agents (NMBAs) (Supplementary Fig. S4). Meta-regression analysis revealed that using NMBAs was negatively associated with the risk of first-attempt insertion failure, and positively associated with OLP, but not associated with POST. The risk of first-attempt insertion failure of using NMBAs in combination with the P-LMA and I-gel was lower than the risk of first-attempt insertion failure of any of the SADs alone. The use of NMBAs in combination with the SLIPA, Solus Standard Laryngeal Mask Airway (Solus), Cobra and LTS-D achieved even higher OLP. Supplementary Tables S20 to S25 present the results of subgroup analysis stratified according to the use of NMBA. Meta-regression analysis indicated the positive pressure ventilation was not associated with POST (*P* = 0.97).

### Publication bias

Comparison-adjusted funnel plots (Supplementary Figs. S8 to S10) and Egger’s regression test for study outcomes (OLP: *P* = 0.68; the risk of first-attempt insertion failure: *P* = 0.10; POST: *P* = 0.81) indicated that no small study bias was present.

### Inconsistency

The design-by-treatment interaction model (*P* = 0.01) and loop inconsistency model (*P* = 0.002) were inconsistent within the network meta-analysis for the risk of first-attempt insertion failure. Significant inconsistencies between the results of direct and indirect comparisons were observed for the following comparisons: the C-LMA and LT (*P* < 0.001), the Protector and AuraGain (*P* = 0.001), the Protector and S-LMA (*P* = 0.004), the AuraGain and S-LMA (*P* = 0.01), the P-LMA and SLIPA (*P* = 0.03) and the P-LMA and LT (*P* = 0.004). For POST, we observed an inconsistency between direct and indirect comparisons of the C-LMA and P-LMA (*P* = 0.002) and the C-LMA and I-gel (*P* = 0.03). Finally, no evidence of inconsistency between direct and indirect comparisons was observed for OLP.

### Other results

The value of kappa was 0.76. The results of sensitivity analysis of the risk of bias are presented in Supplementary Tables S3, S6, and S9. The results of the grading of recommendation, assessment, development, and evaluation (GRADE) are presented in Supplementary Tables 17, 18 and 19.

## Discussion

Compared with the C-LMA, many SADs achieved a significantly higher OLP, with a mean difference ranging from 3.98 to 9.18 cmH_2_O. The Protector achieved the highest OLP and ranked the best among the SADs. All SAD exhibited similar risks of first-attempt insertion failure, and not significant differences in POST compared with those of the C-LMA. Evidences on other secondary efficacy-related outcomes was limited due to extremely low incidence rates. Although the Protector achieved the highest OLP, its risk of first-attempt insertion failure and POST were similar to those of the C-LMA.

The Protector had the highest OLP, followed by AuraGain, relatively newly developed SAD. The high OLP of the Protector may be attributable to the fact that it is made of medical grade silicone. Moreover, its curve airway tube and inflatable cuff may allow it to better conforms to the anatomical contours of an individual’s hypopharynx^10^. Other SADs also attained better OLP than that of the C-LMA, and this finding is consistent with that of a recent network meta-analysis of SADs for pediatric patients, which indicated that the I-gel, Cobra and P-LMA achieved 3.4 to 4.6 cmH_2_O higher OLP than that of the C-LMA^11^. Our results also revealed substantial differences in the efficacy of the SADs: this could not be observed in previous meta-analyses that pooled various SADs into one group^4–6^. In addition, some of the studies included in these meta-analyses used nonstandard methods of measuring OLP or did not clearly describe their measurement method. By contrast, our network meta-analysis evaluated these SADs separately and only included randomized controlled trials that measured OLP through plateau pressure measurement, thereby providing more accurate estimates.

Our results revealed that all SADs exhibited similar risks of first-attempt insertion failure to that of the C-LMA, consistent with the results of a previous network meta-analysis on pediatric patients^11^. Unlike previous studies^6^, we did not use the criterion of “ease of insertion” because it is a highly subjective, operator-dependent assessment; therefore, we used first-attempt insertion failure instead.

Our analysis indicated that all SADs had a POST comparable to that of the C-LMA in contrast to the results of previous meta-analyses^4,5,12,13^. This discrepancy may be because other meta-analyses have pooled data on patients who had received laparoscopic surgery and those from crossover studies when estimating the POST. We excluded crossover studies account for the carry-over effect. Moreover, patients receiving laparoscopic surgery were also excluded due to the higher OLP required, which would result in a greater risk of perilaryngeal tissue trauma.

Our results also demonstrated that adjunctive NMBAs had a significant effect. However, NMBAs exerted a significant effects on the risk of first-attempt insertion failure and OLP, which disagree with the findings of a previous network meta-analysis in which an NMBA was found to have no significant effects on OLP, the POST, or the risk of first-attempt insertion failure^11^.

Our study has several strengths. Rather than grouping various SADs into one group, we assessed the efficacy of each SAD individually and compares them within the same evidence base. Assessment of both OLP and the risk of first-attempt insertion failure according to SUCRA ranking may provide more complete information on SADs, which can help in selecting the appropriate SAD. This network meta-analysis also enabled us to compare therapies indirectly when no head-to-head trial had been performed and obtain more precise effect estimates by jointly assessing direct and indirect comparisons^8^. Furthermore, we also analysed major and efficacy-associated outcomes. The results of our analysis also provide updated evidence, with implications for using SADs in low-risk adult patients.

This study had some limitation. First, the data in our network meta-analysis were mostly derived from low risk, elective, nonobese patients with a low risk of aspiration. Second, some of our efficacy-related outcomes were reported by a limited number of studies with zero events, thereby resulting in greater uncertainty in our assessment of these outcomes. Third, some of the included studies did not report on NMBA use with SADs, affecting the strength of the meta-regression results on NMBA effects. Fourth, we performed a grey literature search but did not identify additional relevant studies that met our inclusion criteria. Fifth, many anesthetic agents have been developed, and anesthetic strategies have changed over the last 30 years since the C-LMA was developed. We included 108 studies in our network meta-analysis, and this large number of studies may have diluted the impact of the different anesthetic agents within our comparisons. Sixth, the number of patients in our network meta-analysis who were treated with the relatively newer SADs, such as the AuraGain, was much lower than number of patients treated with other SADs, such as the P-LMA or I-gel. A lower number of patients yields a wider confidence interval. More studies are therefore required to confirm the efficacy of these new devices.

In conclusion, SADs including the Proseal LMA, I-gel, Supreme LMA, Streamlined Liner of the Pharynx Airway, SoftSeal, Cobra Perilaryngeal Airway, Air-Q, Laryngeal Tube, and Laryngeal Tube Suction II (LTS-II), Laryngeal Tube Suction Disposable (LTS-D), Protector, and AuraGain, achieve significantly higher OLP and similar risks of first-attempt insertion failure compared with those of the C-LMA. Our data indicate that the Protector may be the best SAD because it achieves the highest OLP and a similar risks of first-attempt insertion failure and similar POST to those of C-LMA.

## Methods

### Data source and search strategy

The protocol for this network meta-analysis was registered with PROSPERO (number: CRD42017065273). We searched the EMBASE, Cochrane Central Register of Controlled Trials, and PubMed databases from their inception to December 2019 for randomized controlled trials that compared at least two types of SADs. We applied no language restrictions and performed a manual literature search. We searched for additional studies in the reference lists of all identified publications, including relevant meta-analyses and systematic reviews. We used the following keywords: I-gel, ProSeal LMA (P-LMA), Classic LMA (C-LMA), Supreme LMA (S-LMA), AuraOnce (Ambu-O), single-use Ambu LMA, Unique LMA (U-LMA), Streamlined Liner of the Pharynx Airway (SLIPA), Solus LMA (Solus), Portex Soft Seal LMA (SoftSeal), Air-Q LMA (Air-Q), Cobra Perilaryngeal Airway (Cobra), Laryngeal Tube (LT), Laryngeal Tube Suction (LTS-II), Laryngeal Tube Disposable (LTS-D), Laryngeal Tube Sonda (LTS), Ambu AuraGain Disposable Laryngeal Mask (AuraGain), LMA Protector Airway (Protector), oropharyngeal leak pressure (OLP), overall insertion failure during the induction, first-time insertion failure, insertion failure at first attempt, failure of the device during the maintenance, improper ventilation during the maintenance, ease of insertion, hypoxia, complications, sore throat, pulmonary aspiration, and aspiration. Details of the search strategy are provided in Supplementary Table S26 and Supplementary Fig. S11.

### Study selection

We included randomized controlled trials on patients, who received elective surgeries under general anesthesia and had an indication for SAD insertion with an American Society of Anesthesiologists (ASA) score of I to III, body mass index (BMI) < 40, and age > 18 years. The SADs identified in the literature included the C-LMA, I-gel, P-LMA, S-LMA, Ambu-O, U-LMA, SLIPA, Solus, SoftSeal, Air-Q, Cobra, LT, LTS-II, LTS-D, LTS, AuraGain, and Protector. We used the C-LMA as the reference because it was the first SAD^14^. The included trials compared at least two SADs and reported one of the following primary or secondary outcomes. The primary outcomes were OLP, the risk of first-attempt insertion failure, and POST within 24 h. The secondary outcomes were overall insertion failure rate, successful insertion but with poor function, device failure during maintenance, aspiration and hypoxia. When OLP was assessed at several time points, we extracted the data recorded immediately after SAD insertion. When OLP was assessed in different head positions, we extracted the data recorded in the neutral head position. The overall insertion failure rate was measured during induction. Successful insertion but with poor function was defined as the SAD need for reinsertion, reposition, manipulation, or failure during intubation following successful insertion. Device failure during maintenance was defined as the need to change to another airway device due to laryngospasm or a hiccup, SAD of a different size was then used in case of poor sealing pressure and the SAD was inserted if necessary during maintenance. Hypoxia was regarded as any episode of hypoxia, hypoxemia or desaturation. The POST was evaluated simply in terms of the record of sore throat within 24 h of surgery. We excluded crossover studies in our analysis of the POST because the causative device could not be ascertained. When analyzing device failure, we excluded studies in which the SAD was not used for intraoperative airway management. We excluded studies in which OLP, rate of device failure during maintenance, hypoxia, aspiration, and POST were measured during laparoscopic surgery because the condition of such surgeries may affect these parameters. However, the OLP measured before the laparoscopic condition in the trials was still extracted in our meta-analysis. Higher OLP may be required for adequate sealing during laparoscopic surgery^15^, resulting in increased risks of device failure, hypoxia, and aspiration^16^. Laparoscopic position than in a supine position^17^, leading to a higher risk of ischemic injury at the oropharyngeal mucosa^18^. Therefore, we excluded trials that measured the POST after laparoscopic surgery. In addition, we extracted studies that measured OLP according to plateau pressure. To measure OLP, the expiratory valve of the circle system is closed at a fixed gas flow and the airway pressure is recorded.

The following studies were excluded: (1) duplicate publications; (2) animal or manikin experimental studies; (3) studies with unclear patient conditions (i.e., no clear definition of BMI or ASA); (4) studies in which the size of the selected SAD was not specified ( or in which clinicians did not follow the manufacturers' instructions; (5) studies with a lack of outcome data; (6) studies with unclear descriptions of outcome measurements; (7) studies involving patients with a higher aspiration risk, such as pregnant patients, patients with a history of gastroesophageal reflux, and those with an insufficient fasting time; and (8) studies of patients undergoing emergency surgery.

### Data extraction and quality assessment

Two investigators (CJ Lai and YC Yeh) independently screened all titles and abstracts and evaluated relevant articles. If a study was deemed eligible by any reviewer, then it was included for full-text review. The two investigators then independently assessed the full texts of the studies, and any disagreement was resolved through consensus among the members of the study team. These two investigators extracted and entered information regarding the following aspects into an electronic database: study design, patient characteristics, interventions, comparisons and outcomes (OLP, the risk of first-attempt insertion failure, POST, insertion failure during induction, device failure during maintenance, poor device function after successful insertion, aspiration, and hypoxia). When the relevant information on design or outcomes was unclear, we contacted the original authors for clarifications. The two investigators also independently evaluated the methodological quality of eligible trials by using the Cochrane Collaboration’s tool for assessing the risk of bias^19^. Disagreements in the evaluation were resolved through consultation with another investigator (YKT). The kappa statistic was used to evaluate the interrater agreement^20^.

### Data synthesis and analysis

We conducted network meta-analyses by using the random effects model proposed by Lu and Ades but implemented within the frequentist statistical framework^21^. Risk ratios were used to measure the relative treatment effect on the risk of first-attempt insertion failure (failed or successful) and the occurrence of postoperative sore throat (yes/no), weighted mean differences were used to measure changes in OLP (cmH_2_O). For crossover trials, we used the adjusting variance approach to address correlations between different procedures within trials^22^. We evaluated the transitivity assumption by comparing the distribution of NMBAs used across studies. We also conducted a meta-regression analysis to assess its impact on our network meta-analysis. We used meta-regression analysis to evaluate whether the injection of NMBAs affected the risk of first-attempt insertion failure, OLP, or POST outcomes, and we performed a subgroup analysis stratified according to NMBA use. We also used a meta-regression to evaluate whether positive pressure ventilation affected the POST. The restricted maximum likelihood method and DerSimonian-Laird method were used to estimate random effect models for network meta-analysis and traditional pairwise meta-analyses, respectively^23^. A risk of bias evaluation and sensitivity analysis were also performed^24^. We further applied the GRADE approach (grading of recommendation, assessment, development, and evaluation) to assess the quality of evidence regarding the primary outcomes (OLP, the risk of first-attempt insertion failure, POST)^25^.

For each outcome, we calculated the ranking probabilities for the different SADs. The SUCRA is a percentage of the mean rank of each treatment. The greater the area under the curve, the higher the ranking of the intervention; that is, the better its performance^26^.

### Publication bias

A comparison-adjusted funnel plot and Egger’s regression test were used to test for publication bias. An asymmetrical funnel plot and the obtainment of *P* < 0.05 for Egger’s test suggested small study bias.

### Inconsistency

To evaluate potential inconsistency between the results of direct and indirect comparisons within the network meta-analysis, we used three models: design-by-treatment interaction, the loop-specific inconsistency, and the node-splitting^27–29^.

All statistical analyses in this study were performed with Stata (version 15.0, StataCorp, College Station, TX).

## Supplementary Information

Supplementary Tables.

Supplementary Figures.
