# Supplementary material for: Comparison of the efficacy of supraglottic airway devices in low-risk adult patients: a network meta-analysis and systematic review
Source: Sci Rep. 2021 Jul 23;11:15074. doi: 10.1038/s41598-021-94114-7 (PMC8302760; doi:10.1038/s41598-021-94114-7)
Supplement: Supplementary file 1 — Supplementary Tables. [file 41598_2021_94114_MOESM1_ESM.docx]

**Comparison of the Efficacy of Supraglottic Airway Devices in Low-Risk Adult Patients: A Network Meta-analysis and Systematic Review**

Chih-Jun Lai^†1,2^, Yi-Chun Yeh^†1,3^, Yu-Kang Tu*****^1,3,4^, Ya-Jung Cheng^5,6^, Chih-Min Liu^2^, Shou-Zen Fan*****^2,6^

Short title: Network meta-analysis of supraglottic airways

†Both authors are contributed equally to this work

* Corresponding author: Yu-Kang Tu and Shou-Zen Fan

Affiliation:

1.Institute of Epidemiology and Preventive Medicine, National Taiwan University

2. Department of Anesthesiology, National Taiwan University Hospital

3. Department of Medical Research, National Taiwan University Hospital

4. Department of Dentistry, National Taiwan University Hospital and School of Dentistry, National Taiwan University

5. Department of Anesthesiology, National Taiwan University Cancer Center

6. Department of Anesthesiology, College of Medicine, National Taiwan University

*Corresponding author: Yu-Kang Tu, DDS, MSc, PhD

Address: Institute of Epidemiology and Preventive Medicine, College of Public Health, National Taiwan University, No. 17, Xu-Zhou Road, Taipei 100, Taiwan

Telephone: +88633668039;

Fax: 886-2-2351-1955

E-mail: yukangtu@ntu.edu.tw

*Corresponding author: Shou-Zen, Fan, MD, PhD

Address: Department of Anesthesiology, National Taiwan University Hospital.

No 7 Zhung Shan S. Road, Taipei, Taiwan

Telephone: +886223123456-65410;

Fax: +886227044688

E-mail: shouzen@gmail.com

**Supplementary table**

Table S1: Summary of studies included in the network meta-analysis………………..4

Table S2: Results for ranking of the oropharyngeal leak pressure…………………...20

Table S3: Results for ranking of the oropharyngeal leak pressure excluded the studies related to the high risk bias…………………………………………………………..21

Table S4: Results for ranking of the risk of first-attempt insertion failure…………………...............................................................................................22

Table S5 Results for ranking of the risk of first-attempt insertion failure (from low to high failure rate) excluded two studies related to the inconsistent effect…………….........................................................................................................23

Table S6 Results for ranking of the risk of first-attempt insertion failure (from low to high failure rate) excluded the studies related to the high risk of bias………………………………………………………………………………….. 24

Table S7: Results for ranking of the postoperative sore throat rate………………….25

Table S8: Results for ranking of the postoperative sore throat rate (from low to high postoperative sore throat rate) excluded two studies related to the inconsistent effect……………………………………………….…………………………………26

Table S9: Results for ranking of the postoperative sore throat rate (from low to high postoperative sore throat rate) the studies related to the high risk of bias…………………………………………………………………………………...27

Table S10: Results of systematic review for overall insertion failure rate during the induction……………………………………………………………………………...28

Table S11: Results of systematic review for function incorrectly after successful insertion………………………………………………………………………………29

Table S12: Results of systematic review for device failure during maintenance……..........................................................................................................30

Table S13: Results of systematic review for hypoxia………………………………..31

Table S14: Results of systematic review for aspiration……………………………...32

Table S15: Risk bias of included studies……………………………………………..33

Table S16: the list of excluded studies……………………………...………………..39

Table S17. Estimates of effects and quality ratings for comparison of different supraglottic airway devices to oropharyngeal leak pressure………...…………...…..41

Table S18. Estimates of effects and quality ratings for comparison of different supraglottic airway devices to the risk of first-attempt insertion failure…..…...…..47

Table S19. Estimates of effects and quality ratings for comparison of different supraglottic airway devices to the postoperative sore throat rate……...……………. 53

Table S20: Results for oropharyngeal leak pressure without neuromuscular blocking agents from network meta-analysis…………………………………………………..58

Table S21: Results for oropharyngeal leak pressure with neuromuscular blocking agents from network meta-analysis…………………………………………………..59

Table S22: Results for the risk of first-attempt insertion failure without neuromuscular blocking agents from network meta-analysis.………………………………………..60

Table S23: Results for the risk of first-attempt insertion failure with neuromuscular blocking agents from network meta-analysis.………………………………………..61

Table S24: Results for the postoperative sore throat rate without neuromuscular blocking agents from network meta-analysis……………….………………………..62

Table S25: Results for the postoperative sore throat rate with neuromuscular blocking agents from network meta-analysis……………….…………………...……………. 63

Table S26: Search strategy…………………………………………………………...64

**Table S1: Summary of studies included in the network meta-analysis.**

| **Study, Year, Country** | **Kinds of Supraglottic Airway Devices** | **No. of Subject** | **Muscle relaxant**  **(yes/no)** | **1^st^ attempt failure**  **(n/No.)** | **Oropharyngeal leak pressure(cmH2O)(No.)** | **Sore throat (n/No.)** |
| --- | --- | --- | --- | --- | --- | --- |
| WJ. Shin et al., 2010, KP^1^ | I-gel  Proseal  Classic | 64  53  50 | yes | 14/64  6/53  8/50 | 27.33 ±6.82(64)  30.00±4.57(53)  23.67 ±5.34(50) | 5/64  5/53  12/50 |
| L.Gasteiger et al., 2010, AU^2^ | Proseal  I-gel | 76  75 | no | 1/76  2/75 | 30.00±7.00(76)  23.00±7.00(75) | - |
| W.H.L.Teoh et al., 2010, SG^3^ | Supreme  I-gel | 50  50 | yes | 3/50  2/50 | 26.40±5.10(50)  25.00±5.70(50) | - |
| B. Sharma et al., 2010, IN^4^ | Proseal  I-gel | 30  30 | yes | 6/30  2/30 | 38.93±3.18(30)  35.63±4.84(30) | - |
| PA. Jadhav et al., 2015, IN^5^ | I-gel  Proseal | 30  30 | no | 1/30  6/30 | 20.07±2.94(30)  25.73±2.21(30) | 1/30  5/30 |
| E.F.F.Chew et al., 2010, MY^6^ | Supreme  I-gel | 45  45 | no | 1/45  3/45 | 25.60±5.10(45)  20.70±5.90(45) | 4/42  2/45 |
| AM. Helmy et al., 2010, EG^7^ | I-gel  Classic | 40  40 | no | 4(/40  8/40 | 25.62±4.90(40)  21.20±7.70(40) | 15/40  15/40 |
| WJ. Jeon et al., 2012, KP^8^ | Proseal  I-gel | 15  15 | yes | 0/15  0/15 | 25.90±5.20(15)  24.30±3.40(15) | - |
| R.Ragazzi et al., 2012, IT^9^ | Supreme  I-gel | 39  41 | no | 9/39  13/41 | 29.63±9.00(39)  24.63±7.50(41) | - |
| G. Chauhan et al., 2013, IN^10^ | Proseal  I-gel | 40  40 | yes | 0/40  0/40 | 29.55±3.53(40)  26.73±2.52(40) | 7(40)  0(40) |
| T.C.R.V. van Zundert et al., 2012, AU^11^ | I-gel  Proseal  Supreme | 50  50  50 | no | 3/50  2/50  0/50 | 30.00±11.00(50)  33.00±7.00(50)  32.00±6.00(50) | 2(50)  1(50)  0(50) |
| D.Cattano et al., 2011, US^12^ | I-gel  Unique | 25  25 | no | 8/25  4/25 | 23.10±7.30(25)  20.90±4.50(25) | - |
| E. Seet et al., 2010, CA^13^ | Supreme  Proseal | 50  49 | no | 1/50  6/49 | 21.00±5.00(50)  25.00±6.00(49) | 10(50)  10(49) |
| N. Joly et al., 2014, CA^14^ | I-gel  Supreme | 50  50 | yes | 7/50  6/50 | 23.00±7.00(46)  21.00±8.00(46) |  |
| R. Polat et al., 2014, TR^15^ | I-gel  Classic | 60  60 | no | 7/60  7/60 | - | 10(59)  5(59) |
| O. Ekinci et al., 2014, TR.^16^ | I-gel  Proseal | 40  40 | yes | 0/40  7/40 | - | - |
| JM. Beleña et al., 2015, ES^17^ | I-gel  Supreme | 69  71 | no | 14/69  4/71 | 28.20±3.90(69)  27.50±4.00(71) | - |
| JM. Beleña et al., 2013, ES^18^ | Supreme  Proseal | 60  60 | no | 2/60  17/60 | 27.00±4.00(60)  31.00±6.00(60) | - |
| S. Chattopadhyay et al., 2013, IN^19^ | Supreme  I-gel | 45  45 | no | 4/45  5/45 | 24.40±4.00  23.60±3.80 | - |
| T. Hoşten et al., 2012, TR^20^ | Proseal  Supreme | 29  30 | yes | 2/29  2/30 | 27.00±4.70(29)  27.80±2.90(30) |  |
| 1. Ali et al., 2009, TR^21^ | Supreme  Classic | 35  35 | yes | - | 34.60±3.10(35)  26.10±2.10(35) |  |
| 1. Kumar et al., 2015, IN^22^ | I-gel  Proseal | 30  30 | yes | 2/30  6/30 | - | - |
| S. Mukadder et al., 2015, TR^23^ | Proseal  Supreme  I-gel | 35  35  35 | yes | 9/35  5/35  2/35 | 23.90±2.40(35)  24.90±2.90(35)  21.00±3.60(35) | - |
| SY. Park et al., 2015, KP^24^ | I-gel  Supreme | 47  46 | yes | 1/47  1/46 | - | - |
| S.Y.NG et al., 2007, SG^25^ | AuraOnce(single-use, Ambu LMA)  Classic | 50  55 | no | 4/50  9/55 | 27.20±5.43(50)  28.55±8.16(55) | 0(50)  2(55) |
| AB Suzanna et al., 2011, MY^26^ | Classic  AuraOnce | 60  58 | no | 8/60  10/58 | - | 20(60)  9(58) |
| AM López et al., 2008, ES^27^ | AuraOnce(single-use, Ambu LMA)  Solus  Unique  Soft Seal | 50  50  50  50 | no | 11/50  23/50  10/50  17/50 | 32.60±6.00(49)  28.55±5.44(46)  30.00±8.15(49)  36.71±6.80(46) |  |
| CS. Strydom et al., 2008, ZA^28^ | Classic  Unique  Soft Seal  CobraPLA | 25  21  25  20 | no | 4/25  5/21  6/25  5/20 |  | - |
| MZ. Ali et al., 2011, EG^29^ | I-gel  Supreme | 30  30 | yes | 8/30  12/30 | 25.50±4.80(30)  21.10±7.60(30) | 1(30)  4(30) |
| H. Francksen et al., 2009, DE^30^ | I-gel  Unique | 40  40 | no | 4/40  6/40 | 29.00±5.00(40)  18.00±5.00(40) |  |
| J. Brimacombe et al., 2002, AU^31^ | Proseal  Classic | 192  192 | no | 33/192  18/192 | 27.00±7.00(189)  22.00±6.00(192) | - |
| L. Gaitini et al., 2006, US^32^ | CobraPLA  Unique | 40  40 | no | 3/40  1/40 | 27.00±7.16(40)  21.00±4.21(40) | 4(40)  6(40) |
| CJ. Chung et al., 2009, KP^33^ | CobraPLA  Proseal | 60  60 | yes | 9/60  3/60 | 27.70±3.70(60)  28.70±4.10(60) | - |
| RE. Galgon et al., 2011, US^34^ | Air-Q  Proseal | 52  48 | no | 6/52  1/48 | 30.00±7.00(52)  30.00±6.00(48) |  |
| S. Amini et al., 2010, IR^35^ | Solus  I-gel | 60  60 | yes | 12/60  14/60 | 22.60±7.70(59)  19.30±7.10(56) | 5(59)  2(56) |
| K. Schebesta et al., 2010, AT^36^ | CobraPLA  Classic | 30  30 | no | 7/30  3/30 | 24.00±4.00(30)  20.00±4.00(30) | - |
| O. Akca et al., 2004, US^37^ | CobraPLA  Classic | 40  41 | no | 3/40  3/38 | 23.00±6.00(40)  18.00±5.00(41) | - |
| 1. Abualhassan et al., 2011, EG^38^ | Proseal  SLIPA | 59  58 | yes | 2/59  3/58 | 38.33±5.03(59)  36.84±3.94(58) |  |
| AV Zundert et al., 2006, BE^39^ | Unique  Soft Seal  CobraPLA | 107  103  110 | no | 6/10  2/103  15/110 | 25.00±6.00(107)  31.00±5.00(103)  30.00±6.00(110) | 8(103)  5(102)  11(105) |
| K. EL-Radaideh et al., 2015, JO^40^ | SLIPA  I-gel | 42  40 | Yes | 2/42  4/40 | 28.76±3.11(42)  25.92±3.65(40) |  |
| YM Choi et al., 2010, KR^41^ | SLIPA  Proseal | 29  30 | Yes | 7/29  2/30 | 33.31±9.11(29)  35.48±1.94(30) | 14(29)  8(30) |
| YC Woo et al., 2011, KR^42^ | SLIPA  Proseal | 49  51 | Yes | 5/49  3/51 | 27.30±5.60(49)  24.70±7.80(51) | - |
| LK Anand et al., 2016, IN^43^ | Supreme  Proseal | 40  40 | yes | 2/40  6/40 | 24.90±5.30(40)  28.40±5.80(40) | - |
| K Hayashi et al., 2012, JP^44^ | Supreme  Proseal | 50  50 | yes | 6/50  8/50 |  | - |
| H. Francksen et al., 2007, DE^45^ | Unique  AuraOnce  Soft Seal | 40  40  40 | no | 5/40  5/40  5/40 |  | 11(40)  0(39(  3(38) |
| DA Fernández et al., 2009, ES^46^ | Supreme  I-gel | 42  43 | no | 2/42  6/43 |  | 4(42)  2(43) |
| SK Mishra et al., 2015, IN^47^ | I-gel  Proseal | 30  30 | yes | 3/30  0/30 | 24.00±4.00(30)  29.00±4.00(30) | - |
| S Taxak et al., 2013, IN^48^ | Proseal  Classic | 50  50 | yes | 3/50  3/50 | - | - |
| YB Shi et al., 2013, CN^49^ | Supreme  Proseal  I-gel | 30  30  30 | yes | 3/30  3/30  2/30 | - | - |
| K Schebesta et al., 2014, AT^50^ | Unique  Air-Q  CobraPLA | 8  11  11 | no | 3/8  2/11  3/8 | - | - |
| AJ van Zundert et al., 2003, UK^51^ | Classic  Soft Seal | 100  100 | no | 3/100  5/100 | - | 20(100)  15(100) |
| SK OH et al., 2012, KP^52^ | SLIPA  Classic | 57  57 | no | 7/57  13/57 | - |  |
| EM Galvin et al., 2007, AN^53^ | CobraPLA  Classic | 20  20 | no | 3/20  1/20 | - | - |
| A Turan et al., 2006, TR^54^ | Classic  CobraPLA | 30  30 | yes | 13/30  1/30 | - | 7(30)  15(30) |
| YK Kim et al., 2007, KP^55^ | CobraPLA  Proseal | 24  19 | yes | 0/24  2/19 | - |  |
| P.P. Lu et al., 2002, AU^56^ | Classic  Proseal | 40  40 | yes | 0/40  7/40 | 19.00±4.00(40)  29.00±6.00(40) | - |
| T.Hosten et al., 2009, TR^57^ | Supreme  Proseal | 30  30 | no | 3/30  5/30 | 26.90±6.60(30)  26.10±5.20(30) |  |
| A.Das et al., 2014,IN^58^ | I-gel  Proseal | 30  30 | yes | 3/30  5/30 |  |  |
| U.Braun et al., 2002, DE^59^ | Proseal  Classic | 145  135 | no | 22(145)  14(135) | 29.88±0.21(145)  21.31±0.21(135) | 11(145)  22(135) |
| T.M.Cook et al., 2005, UK^60^ | Soft Seal  Unique | 50  50 | no | 16(50)  11(50) | 26.75±5.90(45)  23.25±6.24(50) |  |
| Y. Li. et al., 2011, CN^61^ | Proseal  Supreme  I-gel | 40  40  40 | yes | 8(40)  3(40)  3(40) | 28.00±4.00(40)  28.00±3.00(40)  28.00±4.00(40) | - |
| AR.I et al., 2012,MY^62^ | Classic  SLIPA | 31  31 | no | 4(31)  6(31) | - | 6(31)  4(31) |
| B.Bein et al., 2005,DE^63^ | Proseal  Laryngeal tube suction | 30  30 | no | 4(30)  2(30) | - | 6(30)  15(30) |
| J.Brimacombe et al., 2002, AT^64^ | Proseal  Laryngeal tube | 60  60 | yes | 9(60)  8(60) | 33.00±7.00(60)  31.00±8.00(60) | 7(60)  5(60) |
| TM.Cook et al., 2003, JP^65^ | Laryngeal tube  Classic | 36  36 | yes | 5(36)  5(36) | 28.00±7.00(35)  21.00±6.00(36) | 12(35)  11(36) |
| E.Figueredo et al., 2003, ES^66^ | Proseal  Laryngeal tube | 35  35 | no | 8(35)  17(35) | - | - |
| LA. Gaitini et al., 2004, US^67^ | Proseal  Laryngeal tube suction | 72  71 | yes | 18(75)  15(75) | 28.00±7.00(75)  34.00±6.00(75) | 9(72)  4(71) |
| HV.Genzwuerker et al., 2007,DE^68^ | Proseal  Laryngeal tube suction | 49  48 | no | 7(50)  6(50) | 32.00±6.25(49)  33.10±5.50(48) | 3(49)  3(48) |
| J.F.Heuer et al., 2009,DE^69^ | Classic  Proseal  AuraOnce  I-gel | 40  40  40  40 | no | 3(40)  6(40)  3(40)  7(40) | - | - |
| T.Kikuchi et al., 2008,JP^70^ | Proseal  Laryngeal tube suction | 48  37 | yes | 19(48)  8(37) | 23.00±5.84(48)  18.50±6.59(37) | 9(48)  4(37) |
| G.Kini et al.,2014,IN^71^ | I-gel  Proseal | 24  24 | no | 5(24)  6(24) | 23.58±4.90(24)  21.83±5.92(24) | - |
| M.Lange et al., 2007, DE^72^ | Classic  SLIPA | 65  59 | no | 1(65)  1(59) | 24.00±4.00(65)  24.00±6.00(58) | 9(65)  1(59) |
| A.Lee et al., 2009, SG^73^ | Supreme  Proseal | 35  35 | yes | 2(35)  3(35) | - | - |
| S.Russo et al., 2012,DE^74^ | I-gel  Supreme  Laryngeal tube suction-D | 36  37  28 | no | 10(38)  11(39)  19(40) | 25.90±5.60(36)  27.10±5.20(37)  24.00±3.90(28) | - |
| A.Siddiqui et al.,2010,PK^75^ | Classic  I-gel | 50  50 | yes | 7(50)  5(50) | - | 0(50)  0(50) |
| C.Thee et al. 2010,DE^76^ | Laryngeal tube suction  Laryngeal tube suction-D | 30  30 | no | 1(30)  2(30) | - | 15(30)  5(29) |
| H.Roth et al.2005,DE^77^ | Proseal  Laryngeal tube suction | 25  25 | no | 2(25)  2(25) | - | - |
| T.S.Yildiz et al., 2007, TR^78^ | Laryngeal tube  Classic | 66  66 | yes | 10(66)  29(66) | - | 17(66)  10(66) |
| K.Esa et al., 2011,MY^79^ | Laryngeal tube suction  Proseal | 27  27 | yes | 3(27)  1(27) | 33.60±3.60(27)  35.70±5.10(27) | - |
| N.S.Klaver et al., 2007,AN^80^ | Laryngeal tube suction  Proseal | 82  78 | no | 26(82)  21(78) | 25.33±8.31(73)  25.33±7.55(74) | - |
| M.Wrobel et al., 2004, DE^81^ | Laryngeal tube  Classic | 50  50 | no | 5(50)  16(50) | 27.74±7.04(47)  20.29±4.08(44) | - |
| S.Mishra et al., 2015, IN^82^ | Proseal  I-gel | 30  30 | yes | - | 28.00±4.19(30)  22.00±3.23(30) | - |
| S.Park et al., 2009,KP^83^ | Proseal  Laryngeal tube suction  CobraPLA | 45  38  45 | yes |  | 26.60±6.60(45)  30.20±4.00(38)  28.70±4.60(45) |  |
| G.Liew et al., 2016, SG^84^ | I-gel  Supreme  Proseal | 49  50  50 | no | 5(49)  4(50)  14(50) | 27.31±0.92(49)  23.60±0.80(50)  24.44±0.70(50) | 2(49)  14(50)  15(50) |
| M.Somri et al., 2016,IL^85^ | Laryngeal tube suction-D  Supreme | 80  80 | yes | - | 35.00±6.00(80)  33.00±6.00(80) | - |
| J.Belena et al., 2016,ES^86^ | Proseal  Supreme  I-gel | 60  60  60 | no | 17(60)  2(60)  14(60) | 30.80±2.60(60)  29.00±3.80(60)  29.20±3.30(60) | - |
| F.Zand et al., 2007, IR^87^ | Laryngeal tube sonda  Proseal | 50  50 | yes | 6(50)  5(50) | 20.00±8.60(49)  24.10±10.80(49) | 7(48)  3(49) |
| T. Wong et al., 2018, CA^88^ | AuraGain  Supreme | 81  84 | No | 19(81)  5(84) | 26.40±2.80(81)  21.60±3.40(84) | - |
| B.Moser et al., 2018, CH^89^ | Protector  Supreme | 48  48 | no | 2(48)  1(48) | 30.90±7.40(48)  25.60±4.40(48) | 5(48)  5(48) |
| A Laurent et al., 2018, CH^90^ | AuraGain  Protector | 49  49 | yes | 1(49)  13(49) | 28.20±7.00(49)  30.10±6.00(49) | - |
| v. Zundert et al., 2020, AU^91^ (accepted in 2019) | Protector  Supreme | 50  50 | no | 14(50)  22(50) | 31.7±2.9(50)  27.7±3.5(50) | 6(50)  6(50) |
| Chang et al., 2019, R.O.K.^92^ | Protector  I-gel | 55  55 | yes | 5(55)  4(55) | 31±7(55)  27±6(55) | 16(55)  11(55) |
| Shariffuddin et al., 2016,MY.^93^ | AuraGain  Supreme | 50  50 | No | 7(50)  11(50) | 24.1±7.4(49)  23.6±6.2(44) | 5(49)  19(44) |
| Cross-over study | | | | | | |
| I.I.Shariffuddin et al., 2008, MY^94^ | AuraOnce  Classic | 40  40 | Yes | 1(40)  5(40) | 19.20±7.50(39)  15.30±5.20(40) | - |
| TM.Cook. et al., 2003, UK^95^ | Proseal  Laryngeal tube | 32  32 | yes | - | 25.75±7.26(32)  23.50±8.22(30) | - |
| TM.Cook. et al. 2005, UK^96^ | Proseal  Laryngeal tube sonda | 32  32 | yes | 4(32)  10(32) | 27.50±5.32(32)  26.00±7.26(29) | - |
| TM.Cook. et al., 2002, UK^97^ | Proseal  Classic | 180  180 | no | 35(180)  19(180) | 30.00±11.21(178)  18.00±5.98(180) | - |
| M.J.Paech et al., 2005,AU^98^ | Soft Seal  Unique | 162  162 | no | 14(162)  7(162) | - | - |
| C.Janakiraman et al., 2009, UK^99^ | I-gel  Classic | 50  50 | no | 23(50)  7(50) | 19.33±7.67(42)  17.00±7.65(46) | - |
| J.Brimacombe et al., 2004, AT^100^ | Unique  Soft Seal | 90  90 | no | 1(90)  10(90) | 24.00±4.00(90)  25.00±4.00(90) | - |
| J.Brimacombe et al., 2003, AT^101^ | Proseal  Classic | 30  30 | yes |  | 30.00±8.00(30)  22.00±7.00(30) |  |
| H.Tham et al., 2010, SG^102^ | Supreme  Proseal | 60  60 | yes | 2(60)  2(60) | 19.60±5.80(60)  20.90±6.70(60) | - |
| V.Uppal et al.,2009,UK^103^ | I-gel  Unique | 39  39 | yes | 1(39)  0(39) | 25.50±1.86(39)  15.23±0.96(39) |  |
| J.Brimacombe et al., 2001, AU^104^ | Proseal  Classic | 30  30 | no | 0(30)  0(30) |  |  |
| J.Brimacombe et al., 2000, AU^105^ | Proseal  Classic | 60  60 | No | 8(60)  0(60) |  |  |
| S.Eschertzhuber et al., 2009, AU^106^ | Supreme  Proseal | 93  93 | yes | 5(93)  7(93) | - | - |
| G.Sudfir et al., 2007,UK^107^ | AuraOnce  Classic | 50  50 | no | 4(50)  8(50) | - | - |
| U.Weber et al., 2011, AT^108^ | I-gel  Unique | 50  50 | no | 4(50)  4(50) |  |  |

**Note:** Classic, LMA Classic; Unique, Unique LMA; Protector, LMA Protector Airway; AuraGain, Ambu AuraGain Disposable Laryngeal Mask; Proseal, Proseal LMA; I-gel, I-gel supraglottic airway; Supreme, Supreme LMA; SLIPA, Streamlined Liner of the Pharynx Airway; AuraOnce, Ambu AuraOnce; Air-Q, Air-Q Masked Laryngeal Airway.; Soft Seal, The Portex Soft Seal Laryngeal Mask; Solus, Solus Standard Laryngeal Mask Airway; CobraPLA, Cobra Perilaryngeal Airway; LT, Laryngeal Tube; LTSII, Laryngeal Tube Suction II; LTS-D, Laryngeal Tube Disposable; LTS, Laryngeal Tube Sonda.

**Reference for all included studies**

1 Shin, W. J., Cheong, Y. S., Yang, H. S. & Nishiyama, T. The supraglottic airway I-gel in comparison with ProSeal Laryngeal Mask Airway and Classic Laryngeal Mask Airway in anaesthetized patients. *Eur J Anaesthesiol.* **27**, 598-601 (2010).

2 Gasteiger, L., Brimacombe, J., Perkhofer, D., Kaufmann, M. & Keller, C. Comparison of guided insertion of the LMA ProSeal vs the I-gel. *Anaesthesia.* **65**, 913-916 (2010).

3 Teoh, W. H. et al. Comparison of the LMA Supreme vs the I-gel in paralysed patients undergoing gynaecological laparoscopic surgery with controlled ventilation. *Anaesthesia*. **65**, 1173-1179 (2010).

4 Sharma, B., Sehgal, R., Sahai, C. & Sood, J. PLMA vs. I-gel: A Comparative Evaluation of Respiratory Mechanics in Laparoscopic Cholecystectomy. *J Anaesthesiol Clin Pharmacol.* **26**, 451-457 (2010).

5 Jadhav P.A., D. N. P., Tendolkar B.A. I-gel versus Laryngeal Mask Airway-Proseal: Comparison of two supraglottic airway devices in short surgical procedures. *J Anaesthesiol Clin Pharmacol.* **31**, 221-225 (2015).

6 Chew, E. E., Hashim, N. H. & Wang, C. Y. Randomised comparison of the LMA Supreme with the I-gel in spontaneously breathing anaesthetised adult patients. *Anaesth Intensive Care.* **38**, 1018-1022 (2010).

7 Helmy, A. M., Atef, H. M., El-Taher, E. M. & Henidak, A. M. Comparative study between I-gel, a new supraglottic airway device, and Classical Laryngeal Mask Airway in anesthetized spontaneously ventilated patients. *Saudi journal of anaesthesia*. **4**, 131-136 (2010).

8 Jeon, W. J., Cho, S. Y., Baek, S. J. & Kim, K. H. Comparison of the Proseal LMA and intersurgical I-gel during gynecological laparoscopy. *Korean J Anesthesiol.* **63**, 510-514 (2012).

9 Ragazzi, R., Finessi, L., Farinelli, I., Alvisi, R. & Volta, C. A. LMA Supreme vs I-gel--a comparison of insertion success in novices. *Anaesthesia*. 67, 384-388 (2012).

10 Chauhan, G. et al. Comparison of clinical performance of the I-gel with LMA proseal. *J Anaesthesiol Clin Pharmacol.* **29**, 56-60 (2013).

11 Van Zundert, T. C. & Brimacombe, J. R. Similar oropharyngeal leak pressures during anaesthesia with I-gel, LMA-ProSeal and LMA-Supreme Laryngeal Masks. Similar oropharyngeal leak pressures during anaesthesia with i-gel, LMA-ProSeal and LMA-Supreme Laryngeal Masks. *Acta Anaesthesiol Belg*. **63**, 35-41 (2012).

12 Cattano, D. et al. A randomized clinical comparison of the Intersurgical I-gel and LMA Unique in non-obese adults during general surgery. *Minerva Anestesiol.* **77**, 292-297 (2011).

13 Seet, E. et al. Safety and efficacy of Laryngeal Mask Airway Supreme versus Laryngeal Mask Airway ProSeal: a randomized controlled trial. *Eur J Anaesthesiol.* **27**, 602-607 (2010).

14 Joly, N. et al. Randomized prospective trial comparing two supraglottic airway devices: I-gel and LMA-Supreme in paralyzed patients. *Can J Anaesth.* **61**, 794-800 (2014).

15 Polat, R. et al. Comparison of the I-gel and the Laryngeal Mask Airway Classic in terms of clinical performance. *Rev Bras Anestesiol.(English Edition)* **65**, 343-348 (2015).

16 Ekinci, O. et al. The comparison of ProSeal and I-gel laryngeal mask airways in anesthetized adult patients under controlled ventilation. *Saudi Med J.* **36**, 432-436 (2015).

17 Belena, J. M. et al. Randomized comparison of the I-gel with the LMA Supreme in anesthetized adult patients. *Anaesthesist.* 64, 271-276 (2015).

18 Belena, J. M. et al. Comparison of Laryngeal Mask Airway Supreme and Laryngeal Mask Airway Proseal with respect to oropharyngeal leak pressure during laparoscopic cholecystectomy: a randomised controlled trial. *Eur J Anaesthesiol.* **30**, 119-123 (2013).

19 Suman Chattopadhyay, S. G. A Comparative Study of Two Disposable Supraglottic Devices in Diagnostic Laparoscopic in Gynecology. *Journal of South Asian Federation of Obstetrics and Gynaecology.* **5**, 124-128 (2013).

20 Tülay Hoşten, T. Ş. Y., Alparslan Kuş, Mine Solak & Kamil Toker. Comparison of Supreme Laryngeal Mask Airway and ProSeal Laryngeal Mask Airway during Cholecystectomy. *Balkan Med J.* **29**, 314-319 (2012).

21 Ali, A., Canturk, S., Turkmen, A., Turgut, N. & Altan, A. Comparison of the Laryngeal Mask Airway Supreme and Laryngeal Mask Airway Classic in adults. *Eur J Anaesthesiol.* **26**, 1010-1014 (2009).

22 Ajay Kumar, A. S., Uma & Vimal Chandhar. Prospective Randomized Comparative Evaluation of the Clinical Performance of I-gel LMA and Proseal LMA in Patients Undergoing Elective Surgeries. R*esearch Journal of Pharmaceutical, Biological and Chemical Sciences.* **6**, 49-55 (2015).

23 Mukadder, S. et al. Comparison of the Proseal, Supreme, and I-gel SAD in gynecological laparoscopic surgeries. *ScientificWorldJournal.* **2015**, 634320 (2015).

24 Park, S. Y. et al. Comparison of I-gel and LMA Supreme during laparoscopic cholecystectomy. *Korean J Anesth.* **68**, 455-461 (2015).

25 Ng, S. Y., Teoh, W. H. L., Lim, Y. & Cheong, V G. Comparison of the AMBU Laryngeal Mask and the LMA Classic in anaesthetised, spontaneously breathing patients. *Anaesth Intensive Care*. **35**, 57-61 (2007).

26 Suzanna, A. B., Liu, C. Y., Rozaidi, S. W. & Ooi, J. S. Comparison between LMA-Classic and AMBU AuraOnce Laryngeal Mask Airway in patients undergoing elective general anaesthesia with positive pressure ventilation. *Med J Malaysia.* **66**, 304-307 (2011).

27 Lopez, A. M. et al. A clinical evaluation of four disposable laryngeal masks in adult patients. *J Clin Anesth.* **20**, 514-520 (2008).

28 Strydom , C.S.& Le Roux, PJ. A clinical comparison of disposable airway devices. *Southern African Journal of Anaesthesia and Analgesia*. **14**, 31-36 (2008).

29 Ali, M. Z., Ebied, R. S., El-Tawdy, A. F., Refaat, A. I. & Kamal, N. M. Controlled mechanical ventilation with LMA Sureme versus I-gel in anesthetized adult patients. *J Egypt Soc Parasitol.* **41**, 365-378 (2011).

30 Francksen, H. et al. A comparison of the I-gel with the LMA-Unique in non-paralysed anaesthetised adult patients. *Anaesthesia.* **64**, 1118-1124 (2009).

31 Brimacombe, J. et al. A multicenter study comparing the ProSeal and Classic Laryngeal Mask Airway in anesthetized, nonparalyzed patients. *Anesthesiology.* **96**, 289-295 (2002).

32 Gaitini, L. et al. A comparison between the PLA Cobra and the Laryngeal Mask Airway Unique during spontaneous ventilation: a randomized prospective study. *Anesth Analg.* **102**, 631-636 (2006).

33 Chung, C.J., Jang, M. K., Choi, S.R., Lee, S.C. & Lee, J.H. A comparative study of the Cobra perilaryngeal airway and Proseal laryngeal mask airway during laparoscopic cholecystectomy. *Korean J Anesthesiol.* **56**, 151-155 (2009).

34 Galgon, R. E., Schroeder, K. M., Han, S., Andrei, A. & Joffe, A. M. The Air-Q Intubating Laryngeal Airway vs the LMA-ProSeal : a prospective, randomised trial of airway seal pressure. *Anaesthesia*. **66**, (2011).

35 Amini, S. & Khoshfetrat, M. Comparison of the Intersurgical Solus Laryngeal Mask Airway and the I-gel supralaryngeal device. *Anaesthesia*. **65**, 805-809 (2010).

36 Schebesta, K. et al. Exposure to anaesthetic trace gases during general anaesthesia: CobraPLA vs. LMA Classic. *Acta Anaesthesiol Scand.* **54**, 848-854 (2010).

37 Akca, O. et al. The new perilaryngeal airway (CobraPLA) is as efficient as the Laryngeal Mask Airway (LMA) but provides better airway sealing pressures. *Anesth Analg*. **99**, 272-278 (2004).

38 Abdellatif, A. A. & Ali, M. A. Comparison of Streamlined Liner of the Pharynx Airway (SLIPA) with the Laryngeal Mask Airway Proseal for lower abdominal laparoscopic surgeries in paralyzed, anesthetized patients. *Saudi J Anaesth.* **5**, 270-276 (2011).

39 van Zundert, A.V. et al. Comparison of Three Disposable Extraglottic Airway Devices in Spontaneously Breathing AdultsThe LMA-Unique, the Soft Seal Laryngeal Mask, and the Cobra Perilaryngeal Airway. *Anesthesiology.* **104**, 1165-1169 (2006).

40 EL-Radaideh, K., Alloway, A.A.& Hani, D.B. Comparison of the Disposable Streamlined Liner of the Pharynx Airway and the Disposable I-gel in Anaesthetized, Paralyzed Adults: A Randomized Prospective Study. *Anesthesiol Res Pract*. **2015,** 971059 (2015).

41 Choi, Y.M. et al. The clinical effectiveness of the Streamlined Liner of Pharyngeal Airway (SLIPA) compared with the Laryngeal Mask Airway ProSeal during general anesthesia. *Korean J Anesthesiol.* **58**, 450-457 (2010).

42 Woo, Y. C. et al. Less perilaryngeal gas leakage with SLIPA than with LMA-ProSeal in paralyzed patients. *Can J Anaesth.* **58**, 48-54 (2011).

43 Anand, L. K., Goel, N., Singh, M. & Kapoor, D. Comparison of the Supreme and the ProSeal Laryngeal Mask Airway in patients undergoing laparoscopic cholecystectomy: A randomized controlled trial. *Acta Anaesthesiol Taiwan.* **54**, 44-50 (2016).

44 Hayashi, K. et al. [Comparison of the Supreme Laryngeal Mask Airway(SLMA), single use, with the reusable Proseal Laryngeal Mask Airway(PLMA) in anesthetized adult Japanese patients]. *Masui.* **61**, 1048-1052 (2012).

45 Francksen, H. et al. Comparison of LMA Unique, Ambu Laryngeal Mask and Soft Seal Laryngeal Mask during routine surgical procedures. *Eur J Anaesthesiol.* **24**, 134-140 (2007).

46 Fernandez Diez, A. et al. [Supreme Laryngeal Mask Airway vs the I-gel supraglottic airway in patients under general anesthesia and mechanical ventilation with no neuromuscular block: a randomized clinical trial]. *Rev Esp Anestesiol Reanim.* **56**, 474-478 (2009).

47 Mishra, S. K. et al. Effect of pneumoperitoneum and Trendelenberg position on oropharyngeal sealing pressure of I-gel and ProSeal LMA in laparoscopic gynecological surgery: A randomized controlled trial. *Anesth Essays Res.* **9**, 353-358 (2015).

48 Taxak, S., Kaur, K., Kaushik, S. & Singh, R. A randomized study to compare ProSeal Laryngeal Mask Airway with Classic Laryngeal Mask Airway in anesthetized patients. *Egyptian Journal of Anaesthesia*. **29**, 285-290 (2013).

49 Shi, Y. B., Zuo, M. Z., Du, X. H. & Yu, Z. Comparison of the efficacy of different types of laryngeal mask airways in patients undergoing laparoscopic gynecological surgery. *Zhonghua Yi Xue Za Zhi.* **93**, 1978-1980 (2013).

50 Schebesta, K. et al. Distance from the glottis to the grille: the LMA Unique, Air-Q and CobraPLA as intubation conduits: A randomised trial. *Eur J Anaesthesiol.* **31**, 159-165 (2014).

51 Van Zundert, A. A., Fonck, K., Al-Shaikh, B. & Mortier, E. Comparison of the LMA-Classic with the new disposable Soft Seal Laryngeal Mask in spontaneously breathing adult patients. *Anesthesiology.* **99**, 1066-1071 (2003).

52 Oh, S.K., Lim, G.B., Kim, H., Lim, S.H. Comparison of the clinical effectiveness between the Streamlined Liner of Pharyngeal Airway (SLIPA) and the Laryngeal Mask Airway by novice personnel. *Korean J Anesthesiol.* **63**, 136-141 (2012).

53 Galvin, E. M. et al. A Randomized Prospective Study Comparing the Cobra Perilaryngeal Airway and Laryngeal Mask Airway-Classic During Controlled Ventilation for Gynecological Laparoscopy. *Anesth Analg*. 104, 102-105 (2007).

54 Turan, A., Kaya, G., Koyuncu, O., Karamanlioglu, B. & Pamukcu, Z. Comparison of the Laryngeal Mask (LMA) and Laryngeal Tube (LT) with the new perilaryngeal airway (CobraPLA) in short surgical procedures. *Eur J Anaesthesiol.* **23**, 234-238 (2006).

55 Kim, Y.K., Jeon, H.Y., Yang, H.S. Comparison of the Clinical Effectiveness of the Cobra Perilaryngeal Airway and the Proseal Laryngeal Mask Airway during Anesthesia with Controlled Ventilation. *Anesth Pain Med.* **2**, 160-165 (2007).

56 Lu, P. P., Brimacombe, J., Yang, C. & Shyr, M. ProSeal versus the Classic Laryngeal Mask Airway for positive pressure ventilation during laparoscopic cholecystectomy. *Br J Anaesth.* **88**, 824-827 (2002).

57 Hosten, T. et al. A new supraglottic airway device: LMA-Supreme, comparison with LMA-Proseal. *Acta Anaesthesiol Scand.* **53**, 852-857 (2009).

58 Das, A. et al. i-gel in Ambulatory Surgery: A Comparison with LMA-ProSeal in Paralyzed Anaesthetized Patients. *J Clin Diagn Res.* **8**, 80 -84 (2014).

59 Braun, U. et al. [A comparison of the Proseal Laryngeal Mask to the standard Laryngeal Mask on anesthesized, non-relaxed patients]. *Anasthesiol Intensivmed Notfallmed Schmerzther.* **37**, 727-733 (2002).

60 Cook, T. M., Trümpelmann, P., Beringer, R. & Stedeford, J. A randomised comparison of the Portex Softseal Laryngeal Mask Airway with the LMA-Unique during anaesthesia. *Anaesthesia*. **60**, 1218-1225 (2005).

61 Li, Y., L, J., Li, C., Zhang, W. & Yu, D. Comparison of effectiveness of the

ProSeal Laryngeal Mask, the Supreme Laryngeal Mask and the I-gel Laryngeal

Mask with airway management for laparoscopic cholecystectomy in patients. *Chin J Anesthesiol*. **31**, 1146-1148 (2011).

62 Abd Rahman I, et al. Comparison between the Use of LMA and SLIPA in Patients Undergoing Minor Surgeries. *J. surg. Acad.* **2**, 8-13 (2012).

63 Bein, B. et al. A comparison of the Proseal Laryngeal Mask Airway, the Laryngeal Tube S and the oesophageal-tracheal combitube during routine surgical procedures. *Eur J Anaesthesiol.* **22**, 341-346 (2005).

64 Brimacombe, J., Keller, C. & Brimacombe, L. A comparison of the Laryngeal Mask Airway ProSeal and the Laryngeal Tube Airway in paralyzed anesthetized adult patients undergoing pressure-controlled ventilation. *Anesth Analg*. **95**, 770-776 (2002).

65 Cook, T. M., McCormick, B. & Asai, T. Randomized comparison of Laryngeal Tube with Classic Laryngeal Mask Airway for anaesthesia with controlled ventilation. *Br J Anaesth.* **91**, 373-378 (2003).

66 Figueredo, E., Martinez, M. & Pintanel, T. A comparison of the ProSeal Laryngeal Mask and the Laryngeal Tube in spontaneously breathing anesthetized patients. *Anesth Analg*. **96**, 600-605 (2003).

67 Gaitini, L. A. et al. A randomized controlled trial comparing the ProSeal Laryngeal Mask Airway with the Laryngeal Tube Suction in mechanically ventilated patients. *Anesthesiology.* **101**, 316-320 (2004).

68 Genzwuerker, H. V. et al. Prospective randomized comparison of the new Laryngeal Tube Suction LTS II and the LMA-ProSeal for elective surgical interventions. *Acta Anaesthesiol Scand.* **51**, 1373-1377 (2007).

69 Heuer, J. F. et al. Evaluation of the new supraglottic airway devices Ambu AuraOnce and Intersurgical I-gel. *Anaesthesist.* **58**, 813-820 (2009).

70 Kikuchi, T., Kamiya, Y., Ohtsuka, T., Miki, T. & Goto, T. Randomized prospective study comparing the Laryngeal Tube Suction II with the ProSeal Laryngeal

Mask Airway in anesthetized and paralyzed patients. *Anesthesiology.* **109**, 54-60 (2008).

71 Kini, G., Devanna, G.M., Mukkapati, K.R., Chaudhuri, S. & Thomas, D. Comparison of I-gel with proseal LMA in adult patients undergoing elective surgical

procedures under general anesthesia without paralysis: A prospective randomized study. *J Anaesthesiol Clin Pharmacol.* **30**, 183-187 (2014).
72 Lange, M. et al. The Effectiveness and Patient Comfort of the Novel Streamlined Pharynx Airway Liner (SLIPA) Compared with the Conventional Laryngeal Mask Airway in Ophthalmic Surgery. *Anesth Analg.* **104**, 431-434 (2007).

73 Lee, A. K., Tey, J. B., Lim, Y. & Sia, A. T. Comparison of the single-use LMA Supreme with the reusable ProSeal LMA for anaesthesia in gynaecological laparoscopic surgery. *Anaesth Intensive Care.* **37**, 815-819 (2009).

74 Russo, S. G. et al. Randomized comparison of the I-gel, the LMA Supreme, and the Laryngeal Tube Suction-D using clinical and fibreoptic assessments in elective patients. *BMC Anesthesiol.* **12**, 1-9 (2012).

75 Siddiqui, A.S., Raees, U.S., Siddiqui, S.Z., Haider, S. & Raza S.A. Comparison of performance and safety of I-gel with Laryngeal Mask Airway (Classic) for general anaesthesia with controlled ventilation. *Anaesth Pain & Intensive Care.* **14**, 17-20 (2010).

76 Thee, C.S. et al. Laryngeal Tube S II, Laryngeal Tube S Disposable, Fastrach Laryngeal Mask and Fastrach Laryngeal Mask Disposable during elective surgery: a

randomized controlled comparison between reusable and disposable supraglottic airway devices. *Eur J Anaesthesiol.* **27**, 468-472 (2010).
77 Roth, H., Genzwuerker, H.V., Rothhaas, A., Finteis, T. & Schmeck, J. The ProSeal Laryngeal Mask Airway and the Laryngeal Tube Suction for ventilation in

gynaecological patients undergoing laparoscopic surgery. *Eur J Anaesthesiol.* **22**, 117-122 (2005).

78 Yildiz, T. S., Solak, M. & Toker, K. Comparison of Laryngeal Tube with Laryngeal Mask Airway in anaesthetized and paralysed patients. *Eur J Anaesthesiol.* **24**, 620-625 (2007).

79 Esa, K., Azarinah, I., Muhammad, M., Helmi, M.A. & Jaafar, M.Z. A comparison between Laryngeal Tube Suction II Airway and Proseal Laryngeal Mask

Airway in laparascopic surgery. *Med J Malaysia.* **66**, 182-186 (2011).

80 Klaver, N. S., Kuizenga, K., Ballast, A. & Fidler, V. A comparison of the clinical use of the Laryngeal Tube S and the ProSeal Laryngeal Mask Airway by first-month anaesthesia residents in anaesthetised patients. *Anaesthesia.* **62**, 723-727 (2007).

81 Wrobel, M., Grundmann, U., Wilhelm, W., Wagner, S. & Larsen, R. Laryngeal Tube Versus Laryngeal Mask Airway in Anaesthetised Non-Paralysed patients: A Comparison of Handling and Postoperative Morbidity. *Anaesthesist.* **53**, 702-708 (2004).

82 Mishra, S. K. et al. Influence of Head and Neck Position on Oropharyngeal Leak Pressure and Cuff Position with the ProSeal Laryngeal Mask Airway and the I-gel: A Randomized Clinical Trial. *Anesthesiol Res Pract.* **2015**, 705869 (2015).

83 Park, S.H., Han, S.H., Do, S.H., Kim, J.W. & Kim, J.H. The influence of head and neck position on the oropharyngeal leak pressure and cuff position of three

supraglottic airway devices. *Anesth Analg.* **108**, 112-117 (2009).
84 Liew, G. H., Yu, E. D., Shah, S. S. & Kothandan, H. Comparison of the clinical performance of I-gel, LMA Supreme and LMA ProSeal in elective surgery. *Singapore Med J.* **57**, 432-437 (2016).

85 Somri, M. et al. A randomized prospective controlled trial comparing the Laryngeal Tube Suction disposable and the Supreme Laryngeal Mask Airway: the influence of head and neck position on oropharyngeal seal pressure. *BMC Anesthesiol.* **16**, 87 (2016).

86 Beleña, J.M. et al. Use of second generation supra-glottic airway devices during laparoscopic cholecystectomy: a prospective, randomized comparison of

LMA ProsealTM, LMA SupremeTM and I-gel. *Acta Anaesth Belg*. **67**, 121-128 (2016).

87 Zand, F., Amini, A., Sadeghi, S.E., Gureishi, M. & Chohedri, A. A comparison of the Laryngeal Tube-S and Proseal Laryngeal Mask during outpatient surgical

procedures. *Eur J Anaesthesiol.* **24**, 847-851 (2007).
88 Wong, D.T. et al. Comparison of oropharyngeal leak pressure between the Ambu AuraGain and the LMA Supreme supraglottic airways: a randomizedcontrolled trial. *Can J Anaesth.* 65, 797-805 (2018)

89 Moser, B., Keller, C., Audige, L. & Bruppacher, H.R. Oropharyngeal leak pressure of the LMA Protector vs the LMA Supreme; a prospective, randomized,

controlled clinical trial. *Acta Anaesthesiol Scand.* **63**, 322-328 (2018).

90 Moser, B. et al. A prospective, randomized trial of the Ambu AuraGain laryngeal mask versus the LMA Protector Airway in paralyzed, anesthetized adult. *Minerva Anestesiol.* **84**, 684-692 (2018).
91 van Zundert, A. A. J., Wyssusek, K. H., Pelecanos, A., Roets, M. & Kumar, C. M. A prospective randomized comparison of airway seal using the novel visionguided insertion of LMA-Supreme and LMA-Protector. *J Clin Monit Comput.* **34**, 285-294 (2020)

92 Chang, J. E. et al. A prospective, randomized comparison of the LMA-Protector and I-gel in paralyzed, anesthetized patients. *BMC Anesthesiol*. **19**, 118 (2019).
93 Shariffuddin, II, Teoh, W. H., Tang, E., Hashim, N. & Loh, P. S. Ambu AuraGain versus LMA Supreme Second Seal: a randomised controlled trial comparing oropharyngeal leak pressures and gastric drain functionality in spontaneously breathing patients. *Anaesth Intensive Care.* **45**, 244-250 (2017).

94 Shariffuddin, II. & Wang, C.Y. Randomised crossover comparison of the Ambu AuraOnce Laryngeal Mask with the LMA Classic Laryngeal Mask Airway in

paralysed anaesthetised patients. *Anaesthesia.* **63**, 82-85 (2008)

95 Cook, T.M., McKinstry, C., Hardy, R. & Twigg, S. Randomized crossover comparison of the ProSeal Laryngeal Mask Airway with the Laryngeal Tube during

anaesthesia with controlled ventilation. *Br J Anaesth.* **91**, 678-683 (2003).

96 Cook, T.M. & Cranshaw, J. Randomized crossover comparison of ProSeal Laryngeal Mask Airway with Laryngeal Tube Sonda during anaesthesia with

controlled ventilation. *Br J Anaesth*. **95,** 261-266 (2005).
97 Cook, T.M. et al. Randomized crossover comparison of the Proseal with the Classic Laryngeal Mask Airway in unparalysed anaesthetized patients. *Br J Anaesth.* **88,** 527-533 (2002).

98 Paech, M.J. et al. Randomised, crossover comparison of the single-use SoftSeal and the LMA Unique Laryngeal Mask Airways. *Anaesthesia.* **60**, 354-359 (2005).

99 Janakiraman, C., Chethan, D.B., Wilkes, A.R., Stacey, M.R. & Goodwin, N. A randomised crossover trial comparing the I-gel supraglottic airway and Classic Laryngeal Mask Airway. *Anaesthesia.* **64**, 674-678 (2009).

100 Brimacombe, J., von Goedecke, A., Keller, C., Brimacombe, L. & Brimacombe, M. The Laryngeal Mask Airway Unique versus the Soft Seal Laryngeal Mask: a

randomized, crossover study in paralyzed, anesthetized patients. *Anesth Analg.* **99**, 1560-1563 (2004).

101 Brimacombe, J. & Keller, C. Stability of the LMA-ProSeal and standard laryngeal mask airway in different head and neck positions: a randomized crossover study. *Eur J Anaesthesiol.* **20**, 65-69, (2003).

102 Tham, H.M., Tan, S.M., Woon, K.L. & Zhao, Y.D. A comparison of the Supreme Laryngeal Mask Airway with the Proseal Laryngeal Mask Airway in anesthetized paralyzed adult patients: a randomized crossover study. *Can J Anaesth.* **57,** 672-678 (2010).
103 Uppal, V., Gangaiah, S., Fletcher, G. & Kinsella, J. Randomized crossover comparison between the I-gel and the LMA-Unique in anaesthetized, paralysed adults. *Br J Anaesth.* **103**, 882-885 (2009).

104 Brimacombe, J., Keller, C., Boehler, M. & Puhringer, F. Positive pressure ventilation with the ProSeal versus Classic Laryngeal Mask Airway: a randomized, crossover study of healthy female patients. *Anesth Analg.* **93,** 1351-1353 (2001).

105 Brimacombe, J. & Keller, C. The ProSeal laryngeal mask airway: A randomized, crossover study with the standard Laryngeal Mask Airway in paralyzed, anesthetized patients. *Anesthesiology.* **93**, 104-109 (2000).

106 Eschertzhuber, S., Brimacombe, J., Hohlrieder, M. & Keller, C. The Laryngeal Mask Airway Supreme--a single use Laryngeal Mask Airway with an

oesophageal vent. A randomised, cross-over study with the Laryngeal Mask Airway ProSeal in paralysed, anaesthetised patients. *Anaesthesia.* **64**, 79-83 (2009).

107 Sudhir, G., Redfern, D., Hall, J.E., Wilkes, A.R. & Cann, C. A comparison of the disposable Ambu AuraOnce Laryngeal Mask with the reusable LMA Classic Laryngeal Mask Airway. *Anaesthesia.* **62,** 719-722 (2007).
108 Weber, U. et al. Comparison of the i-gel and the LMA-Unique Laryngeal Mask Airway in patients with mild to moderate obesity during elective short-term surgery. *Anaesthesia.* **66,** 481-487 (2011).

**Table S2:** **Results for ranking of the oropharyngeal leak pressure**

|  | **Treatment** | **SUCRA** | **Mean Rank** |
| --- | --- | --- | --- |
| 1 | Protector | 94.1 | 2.0 |
| 2 | AuraGain | 81.4 | 4.0 |
| 3 | LTS-II | 80.7 | 4.1 |
| 4 | P-LMA | 76.8 | 4.7 |
| 5 | Air-Q | 69.4 | 5.9 |
| 6 | LT | 62.6 | 7.0 |
| 7 | SLIPA | 56.6 | 8.0 |
| 8 | Cobra | 56.5 | 8.0 |
| 9 | S-LMA | 51.0 | 8.8 |
| 10 | SoftSeal | 47.1 | 9.5 |
| 11 | LTS-D | 43.3 | 10.1 |
| 12 | LTS | 43.3 | 10.1 |
| 13 | I-gel | 36.0 | 11.2 |
| 14 | Ambu-O | 24.0 | 13.2 |
| 15 | Solus | 16.2 | 14.4 |
| 16 | C-LMA | 6.7 | 16.0 |
| 17 | U-LMA | 4.4 | 16.3 |

Note: SUCRA, surface under the cumulative ranking curve; C-LMA, LMA Classic; U-LMA, Unique LMA; Protector, LMA Protector Airway; AuraGain, Ambu AuraGain Disposable Laryngeal Mask; P-LMA, Proseal LMA; I-gel, I-gel supraglottic airway; S-LMA, Supreme LMA; SLIPA, Streamlined Liner of the Pharynx Airway; Ambu-O, Ambu AuraOnce; Air-Q, Air-Q Masked Laryngeal Airway.; SoftSeal, The Portex Soft Seal Laryngeal Mask; Solus, Solus Standard Laryngeal Mask Airway; Cobra, Cobra Aerilaryngeal Airway; LT, Laryngeal Tube; LTSII, Laryngeal Tube Suction II; LTS-D, Laryngeal Tube Disposable; LTS, Laryngeal Tube Sonda.

.

**Table S3:** **Results for ranking of the oropharyngeal leak pressure excluded the studies related to the high- risk bias**

|  | Treatment | SUCRA | Mean Rank |
| --- | --- | --- | --- |
| 1 | LTS-II | 85.9 | 3.0 |
| 2 | Protector | 81.9 | 3.5 |
| 3 | Cobra | 79.5 | 3.9 |
| 4 | P-LMA | 79.1 | 3.9 |
| 5 | Air-Q | 73.7 | 4.7 |
| 6 | S-LMA | 61.7 | 6.4 |
| 7 | LT | 58.8 | 6.8 |
| 8 | LTS-D | 52.2 | 7.7 |
| 9 | LTS | 49.7 | 8.0 |
| 10 | i-gel | 41.9 | 9.1 |
| 11 | Ambu-O | 36.9 | 9.8 |
| 12 | SLIPA | 24.0 | 11.6 |
| 13 | C-LMA | 14.5 | 13.0 |
| 14 | SoftSeal | 8.9 | 13.8 |
| 15 | U-LMA | 1.3 | 14.8 |

Note: SUCRA, surface under the cumulative ranking curve; C-LMA, LMA Classic; U-LMA, Unique LMA; Protector, LMA Protector Airway; AuraGain, Ambu AuraGain Disposable Laryngeal Mask; P-LMA, Proseal LMA; I-gel, I-gel supraglottic airway; S-LMA, Supreme LMA; SLIPA, Streamlined Liner of the Pharynx Airway; Ambu-O, Ambu AuraOnce; Air-Q, Air-Q Masked Laryngeal Airway.; SoftSeal, The Portex Soft Seal Laryngeal Mask; Solus, Solus Standard Laryngeal Mask Airway; Cobra, Cobra Perilaryngeal Airway; LT, Laryngeal Tube; LTSII, Laryngeal Tube Suction II; LTS-D, Laryngeal Tube Disposable; LTS, Laryngeal Tube Sonda.

.

**Table S4 Results for ranking of the risk of first-attempt insertion failure (from low to high failure rate)**

|  | **Treatment** | **SUCRA** | **MeanRank** |
| --- | --- | --- | --- |
| 1 | Ambu-O | 82.1 | 3.9 |
| 2 | S-LMA | 81.8 | 3.9 |
| 3 | U-LMA | 75.9 | 4.9 |
| 4 | Air-Q | 74.4 | 5.1 |
| 5 | AuraGain | 72.2 | 5.5 |
| 6 | LT | 66.1 | 6.4 |
| 7 | Protector | 57.6 | 7.8 |
| 8 | i-gel | 52.1 | 8.7 |
| 9 | C-LMA | 50.5 | 8.9 |
| 10 | SoftSeal | 45.4 | 9.7 |
| 11 | LTS-II | 39.1 | 10.7 |
| 12 | Solus | 36.5 | 11.2 |
| 13 | SLIPA | 31.4 | 12 |
| 14 | LTS-D | 27.5 | 12.6 |
| 15 | Cobra | 26.1 | 12.8 |
| 16 | P-LMA | 23.8 | 13.2 |
| 17 | LTS | 7.4 | 15.8 |

Note: SUCRA, surface under the cumulative ranking curve; C-LMA, LMA Classic; U-LMA, Unique LMA; Protector, LMA Protector Airway; AuraGain, Ambu AuraGain Disposable Laryngeal Mask; P-LMA, Proseal LMA; I-gel, I-gel supraglottic airway; S-LMA, Supreme LMA; SLIPA, Streamlined Liner of the Pharynx Airway; Ambu-O, Ambu AuraOnce; Air-Q, Air-Q Masked Laryngeal Airway.; SoftSeal, The Portex Soft Seal Laryngeal Mask; Solus, Solus Standard Laryngeal Mask Airway; Cobra, Cobra Perilaryngeal Airway; LT, Laryngeal Tube; LTSII, Laryngeal Tube Suction II; LTS-D, Laryngeal Tube Disposable; LTS, Laryngeal Tube Sonda.

. .

**Table S5 Results for ranking of the risk of first-attempt insertion failure (from low to high failure rate) excluded two studies related to the inconsistent effect**

|  | **Treatment** | **SUCRA** | **MeanRank** |
| --- | --- | --- | --- |
| 1 | LT | 85.8 | 3.3 |
| 2 | Ambu-O | 82 | 3.9 |
| 3 | S-LMA | 75.7 | 4.9 |
| 4 | U-LMA | 74.8 | 5 |
| 5 | Air-Q | 74.8 | 5 |
| 6 | AuraGain | 66.6 | 6.3 |
| 7 | Protector | 58.3 | 7.7 |
| 8 | C-LMA | 53.9 | 8.4 |
| 9 | SLIPA | 49.9 | 9 |
| 10 | I-gel | 46.4 | 9.6 |
| 11 | SoftSeal | 45 | 9.8 |
| 12 | Solus | 33.8 | 11.6 |
| 13 | LTS-II | 31.8 | 11.9 |
| 14 | Cobra | 25.4 | 12.9 |
| 15 | LTS-D | 22.4 | 13.4 |
| 16 | P-LMA | 18.6 | 14 |
| 17 | LTS | 4.8 | 16.2 |

Note: SUCRA, surface under the cumulative ranking curve; C-LMA, LMA Classic; U-LMA, Unique LMA; Protector, LMA Protector Airway; AuraGain, Ambu AuraGain Disposable Laryngeal Mask; P-LMA, Proseal LMA; I-gel, I-gel supraglottic airway; S-LMA, Supreme LMA; SLIPA, Streamlined Liner of the Pharynx Airway; Ambu-O, Ambu AuraOnce: Air-Q, Air-Q Masked Laryngeal Airway.; SoftSeal, The Portex Soft Seal Laryngeal Mask; Solus, Solus Standard Laryngeal Mask Airway; Cobra, Cobra Perilaryngeal Airway; LT, Laryngeal Tube; LTSII, Laryngeal Tube Suction II; LTS-D, Laryngeal Tube Disposable; LTS, Laryngeal Tube Sonda.

**Table S6 Results for ranking of the risk of first-attempt insertion failure (from low to high failure rate) excluded the studies related to the high risk of bias**

|  | **Treatment** | **SUCRA** | **MeanRank** |
| --- | --- | --- | --- |
| 1 | Ambu-O | 88.5 | 2.6 |
| 2 | S-LMA | 82.3 | 3.5 |
| 3 | AuraGain | 75.2 | 4.5 |
| 4 | LT | 64.7 | 5.9 |
| 5 | U-LMA | 62.6 | 6.2 |
| 6 | Protector | 59.1 | 6.7 |
| 7 | Solus | 58.5 | 6.8 |
| 8 | C-LMA | 50.1 | 8 |
| 9 | I-gel | 50.1 | 8 |
| 10 | LTS-II | 41.1 | 9.3 |
| 11 | LTS-D | 34.2 | 10.2 |
| 12 | P-LMA | 29.1 | 10.9 |
| 13 | SoftSeal | 27.8 | 11.1 |
| 14 | SLIPA | 13.7 | 13.1 |
| 15 | LTS | 12.9 | 13.2 |

Note: SUCRA, surface under the cumulative ranking curve; C-LMA, LMA Classic; U-LMA, Unique LMA; Protector, LMA Protector Airway; AuraGain, Ambu AuraGain Disposable Laryngeal Mask; P-LMA, Proseal LMA; I-gel, I-gel supraglottic airway; S-LMA, Supreme LMA; SLIPA, Streamlined Liner of the Pharynx Airway; Ambu-O, Ambu AuraOnce, Air-Q, Air-Q Masked Laryngeal Airway.; SoftSeal, The Portex Soft Seal Laryngeal Mask; Solus, Solus Standard Laryngeal Mask Airway; Cobra, Cobra Perilaryngeal Airway; LT, Laryngeal Tube; LTSII, Laryngeal Tube Suction II.

**Table S7 Results for ranking of the postoperative sore throat rate (from low to high postoperative sore throat rate)**

|  | **Treatment** | **SUCRA** | **MeanRank** |
| --- | --- | --- | --- |
| 1 | AuraGain | 92.8 | 2.1 |
| 2 | Ambu-O | 85.8 | 3.1 |
| 3 | I-gel | 77.9 | 4.3 |
| 4 | SLIPA | 58.4 | 7.2 |
| 5 | LTS-II | 57.7 | 7.3 |
| 6 | P-LMA | 55.6 | 7.7 |
| 7 | Protector | 50.7 | 8.4 |
| 8 | SoftSeal | 47.7 | 8.8 |
| 9 | S-LMA | 45.5 | 9.2 |
| 10 | C-LMA | 45.4 | 9.2 |
| 11 | LT | 40.1 | 10.0 |
| 12 | Solus | 34.7 | 10.8 |
| 13 | U-LMA | 32.2 | 11.2 |
| 14 | LTS-D | 28.1 | 11.8 |
| 15 | LTS | 25.2 | 12.2 |
| 16 | Cobra | 22.2 | 12.7 |

Note: SUCRA, surface under the cumulative ranking curve; C-LMA, LMA Classic, U-LMA, Unique LMA; Protector: LMA Protector Airway; AuraGain: Ambu AuraGain Disposable Laryngeal Mask; P-LMA, Proseal LMA; I-gel, I-gel supraglottic airway; S-LMA, Supreme LMA; SLIPA, Streamlined Liner of the Pharynx Airway; Ambu-O, Ambu AuraOnce; SoftSeal, The Portex Soft Seal Laryngeal Mask; Solus, Solus Standard Laryngeal Mask Airway; Cobra, Cobra Perilaryngeal Airway; LT, Laryngeal Tube; LTSII, Laryngeal Tube Suction II; LTS, Laryngeal Tube Sonda.

.

**Table S8 Results for ranking of the postoperative sore throat rate (from low to high postoperative sore throat rate) excluded two studies related to the inconsistent effect**

|  | **Treatment** | **SUCRA** | **MeanRank** |
| --- | --- | --- | --- |
| 1 | AuraGain | 93.4 | 2.0 |
| 2 | Ambu-O | 85.8 | 3.1 |
| 3 | P-LMA | 66.9 | 6.0 |
| 4 | I-gel | 66.6 | 6.0 |
| 5 | LTS-II | 66.6 | 6.0 |
| 6 | SLIPA | 60.9 | 6.9 |
| 7 | SoftSeal | 47.2 | 8.9 |
| 8 | S-LMA | 45.2 | 9.2 |
| 9 | Protector | 45.0 | 9.3 |
| 10 | C-LMA | 43.4 | 9.5 |
| 11 | LT | 40.7 | 9.9 |
| 12 | LTS-D | 31.0 | 11.3 |
| 13 | U-LMA | 30.5 | 11.4 |
| 14 | LTS | 29.8 | 11.5 |
| 15 | Solus | 26.9 | 12.0 |
| 16 | Cobra | 20.0 | 13.0 |

Note: SUCRA, surface under the cumulative ranking curve; C-LMA, LMA Classic; U-LMA, Unique LMA; Protector, LMA Protector Airway; AuraGain, Ambu AuraGain Disposable Laryngeal Mask; P-LMA, Proseal LMA; I-gel, I-gel supraglottic airway; S-LMA, Supreme LMA; SLIPA, Streamlined Liner of the Pharynx Airway; Ambu-O, Ambu AuraOnce; SoftSeal, The Portex Soft Seal Laryngeal Mask; Solus, Solus Standard Laryngeal Mask Airway; Cobra, Cobra Perilaryngeal Airway; LT, Laryngeal Tube; LTSII, Laryngeal Tube Suction II; LTS-D, Laryngeal Tube Disposable; LTS, Laryngeal Tube Sonda.

**Table S9 Results for ranking of the postoperative sore throat rate (from low to high postoperative sore throat rate) excluded the studies related to the high risk of bias**

|  | **Treatment** | **SUCRA** | **MeanRank** |
| --- | --- | --- | --- |
| 1 | AuraGain | 90.3 | 2.2 |
| 2 | Ambu-O | 77.8 | 3.7 |
| 3 | I-gel | 72.6 | 4.3 |
| 4 | SLIPA | 55.2 | 6.4 |
| 5 | LTS-II | 51 | 6.9 |
| 6 | P-LMA | 50.1 | 7 |
| 7 | Protector | 48.2 | 7.2 |
| 8 | S-LMA | 45.7 | 7.5 |
| 9 | C-LMA | 39.5 | 8.3 |
| 10 | LT | 36.5 | 8.6 |
| 11 | Solus | 34.5 | 8.9 |
| 12 | LTS-D | 27.5 | 9.7 |
| 13 | LTS | 21.3 | 10.4 |

Note: SUCRA, surface under the cumulative ranking curve; C-LMA, LMA Classic; U-LMA, Unique LMA; Protector, LMA Protector Airway; AuraGain, Ambu AuraGain Disposable Laryngeal Mask; P-LMA, Proseal LMA; I-gel, I-gel supraglottic airway; S-LMA, Supreme LMA; SLIPA, Streamlined Liner of the Pharynx Airway; Ambu-O, Ambu AuraOnce; SoftSeal, The Portex Soft Seal Laryngeal Mask; Solus, Solus Standard Laryngeal Mask Airway; Cobra, Cobra Perilaryngeal Airway; LT, Laryngeal Tube.

**Table S10: Results of systematic review for the occurrence of patients with overall insertion failure rate during the induction**

| **Supraglottic airway devices** | **Total patient number of overall failure insertion reported** | **Patient number of occurrences** | **Incidence (%)** |
| --- | --- | --- | --- |
| **C-LMA** | **2048** | **26** | **1.27** |
| **P-LMA** | **3032** | **20** | **0.66** |
| **i-gel** | **1777** | **44** | **2.48** |
| **S-LMA** | **1549** | **13** | **0.84** |
| **Ambu-O** | **318** | **2** | **0.63** |
| **U-LMA** | **739** | **6** | **0.82** |
| **SLIPA** | **325** | **5** | **1.53** |
| **Solus** | **110** | **5** | **4.55** |
| **SoftSeal** | **548** | **12** | **2.19** |
| **Cobra** | **385** | **11** | **2.86** |
| **Air-Q®** | **11** | **0** | **0** |
| **LT** | **279** | **12** | **4.30** |
| **LTSII** | **356** | **15** | **4.21** |
| **LTS-D** | **70** | **13** | **18.57** |
| **LTS** | **114** | **1** | **0.87** |

Note: C-LMA, LMA Classic; U-LMA, Unique LMA; Protector, LMA Protector Airway; AuraGain, Ambu AuraGain Disposable Laryngeal Mask; P-LMA, Proseal LMA; I-gel, I-gel supraglottic airway; S-LMA, Supreme LMA; SLIPA, Streamlined Liner of the Pharynx Airway; Ambu-O, Ambu AuraOnce; Air-Q, Air-Q Masked Laryngeal Airway.; SoftSeal, The Portex Soft Seal Laryngeal Mask; Solus, Solus Standard Laryngeal Mask Airway; Cobra, Cobra Perilaryngeal Airway; LT, Laryngeal Tube; LTSII, Laryngeal Tube Suction II; LTS-D, Laryngeal Tube Disposable; LTS, Laryngeal Tube Sonda.

.

**Table S11: Results of systematic review for** **the occurrence of patients with device function incorrectly after successful insertion**

| **Supraglottic airway devices** | **Total patient number** | **Patient number of occurrences** | **Incidence (%)** |
| --- | --- | --- | --- |
| **C-LMA** | **185** | **0** | **0** |
| **P-LMA** | **419** | **4** | **0.95** |
| **I-gel** | **447** | **6** | **1.34** |
| **S-LMA** | **142** | **4** | **2.82** |
| **Ambu-O** | **139** | **13** | **9.35** |
| **U-LMA** | **49** | **3** | **6.12** |
| **SLIPA** | **89** | **4** | **4.49** |
| **Solus** | **46** | **10** | **21.73** |
| **SoftSeal** | **46** | **8** | **17.39** |
| **LTS-II** | **121** | **0** | **0** |
| **LTS** | **29** | **0** | **0** |

Note: C-LMA, LMA Classic; U-LMA, Unique LMA; P-LMA, Proseal LMA; I-gel, I-gel supraglottic airway; S-LMA, Supreme LMA; SLIPA, Streamlined Liner of the Pharynx Airway; Ambu-O, Ambu AuraOnce; SoftSeal, The Portex Soft Seal Laryngeal Mask; Solus, Solus Standard Laryngeal Mask Airway; LTSII, Laryngeal Tube Suction II; LTS, Laryngeal Tube Sonda.

**Table S12: Results of systematic review for the occurrence of patients with device failure during the maintenance**

| **Supraglottic airway devices** | **Total patient number** | **Patient number of occurrences** | **Incidence (%)** |
| --- | --- | --- | --- |
| **C-LMA** | **487** | **0** | **0** |
| **P-LMA** | **633** | **2** | **0.32** |
| **I-gel** | **661** | **12** | **1.82** |
| **S-LMA** | **177** | **6** | **3.39** |
| **Ambu-O** | **39** | **0** | **0** |
| **U-LMA** | **140** | **0** | **0** |
| **SLIPA** | **58** | **0** | **0** |
| **Solus** | **59** | **0** | **0** |
| **SoftSeal** | **145** | **2** | **1.38** |
| **Cobra** | **70** | **0** | **0** |
| **LT** | **181** | **4** | **2.21** |
| **LTS-II** | **229** | **0** | **0** |
| **LTS** | **14** | **1** | **7.14** |

Note: C-LMA, LMA Classic; P-LMA, LMA ProSeal; I-gel, i-gel supraglottic airway; S-LMA, LMA Supreme; Ambu-O, Ambu AuraOnce; U-LMA, LMA Unique; SLIPA, Streamlined Liner of the Pharynx Airway; Solus, Solus Standard Laryngeal Mask Airway; SoftSeal, The Portex Soft Seal Laryngeal Mask; Cobra, Cobra Perilaryngeal Airway; LT, Laryngeal Tube; LTSII, Laryngeal Tube Suction II; LTS, Laryngeal Tube Sonda.

**Table S13: Results of systematic review for the occurrence of patients with hypoxia**

| **Supraglottic airway devices** | **Total patient number of hypoxia reported** | **Patient number of occurrences of hypoxia** | **Incidence (%)** |
| --- | --- | --- | --- |
| **C-LMA** | **156** | **0** | **0** |
| **P-LMA** | **279** | **0** | **0** |
| **I-gel** | **509** | **0** | **0** |
| **S-LMA** | **196** | **0** | **0** |
| **Ambu-O** | **39** | **0** | **0** |
| **U-LMA** | **183** | **0** | **0** |
| **Solus** | **59** | **0** | **0** |
| **SoftSeal** | **140** | **0** | **0** |
| **Cobra** | **135** | **0** | **0** |
| **LT** | **96** | **4** | **4.17** |
| **AuraGain** | **49** | **0** | **0** |
| **Protector** | **97** | **0** | **0** |

Note: C-LMA, LMA Classic; U-LMA, Unique LMA; Protector, LMA Protector Airway; AuraGain, Ambu AuraGain Disposable Laryngeal Mask; P-LMA, Proseal LMA; I-gel, I-gel supraglottic airway; S-LMA, Supreme LMA; SLIPA, Streamlined Liner of the Pharynx Airway; Ambu-O, Ambu AuraOnce; Air-Q, Air-Q Masked Laryngeal Airway.; SoftSeal, The Portex Soft Seal Laryngeal Mask; Solus, Solus Standard Laryngeal Mask Airway; Cobra, Cobra Perilaryngeal Airway; LT, Laryngeal Tube; LTSII, Laryngeal Tube Suction II; LTS-D, Laryngeal Tube Disposable; LTS, Laryngeal Tube Sonda;.

**Table S14: Results of systematic review for the occurrence of patients with aspiration**

| **Supraglottic airway devices** | **Total patient number of hypoxia reported** | **Patient number of occurrence of hypoxia** |
| --- | --- | --- |
| **C-LMA** | **105** | **0** |
| **P-LMA** | **296** | **0** |
| **I-gel** | **124** | **0** |
| **S-LMA** | **50** | **0** |
| **Ambu-O** | **50** | **0** |
| **LT** | **60** | **0** |
| **LTS** | **73** | **0** |
| **AuraGain** | **50** | **0** |

Note: C-LMA, LMA Classic; P-LMA, LMA ProSeal; I-gel, I-gel supraglottic airway; S-LMA, LMA Supreme; Ambu-O, Ambu AuraOnce; LT, Laryngeal Tube; LTS, Laryngeal Tube Sonda; AuraGain: Ambu AuraGain Disposable Laryngeal Mask.

**Table S15: Risk bias of included studies**

| **Study, Year, Country** | **Random sequence generation**  **(selection bias)** | **Allocation concealment**  **(selection bias)** | **Blinding of participants and personnel**  **(Performance bias)** | **Blinding of outcome assessment**  **(detection bias)** | **Incomplete outcome data**  **(attrition bias)** | **Selective report**  **(reporting bias)** |
| --- | --- | --- | --- | --- | --- | --- |
| WJ. Shin et al., 2010, KP | low | low | low | high | low | low |
| L.Gasteiger et al., 2010, AU | low | low | low | unclear | low | low |
| W.H.L.Teoh et al., 2010, SG | low | low | high | low | low | low |
| B. Sharma et al., 2010, IN | low | low | unclear | unclear | low | low |
| PA. Jadhav et al., 2015, IN | low | low | unclear | unclear | low | low |
| E.F.F.Chew et al., 2010, MY | low | low | low | low | low | low |
| AM. Helmy et al., 2010, EG | low | unlcear | unclear | unclear | low | low |
| WJ. Jeon et al., 2012, KP | low | low | high | high | low | low |
| R.Ragazzi et al., 2012, IT | low | low | unclear | unclear | low | low |
| G. Chauhan et al., 2013, IN | low | low | unclear | low | low | low |
| T.C.R.V. van Zundert et al., 2012, AU | low | low | unclear | unlcear | low | low |
| D.Cattano et al., 2011, US | low | low | high | high | low | low |
| E. Seet et al., 2010, CA | low | low | unclear | unclear | low | low |
| N. Joly et al., 2014, CA | low | low | high | unclear | low | low |
| R. Polat et al., 2014, TR | low | low | high | low | low | low |
| O. Ekinci et al., 2014, TR. | low | low | high | high | low | low |
| JM. Beleña et al., 2015, ES | low | low | high | unlcear | low | low |
| JM. Beleña et al., 2013, ES | low | low | high | low | low | low |
| S. Chattopadhyay et al., 2013, IN | low | low | unclear | unlcear | Low | low |
| T. Hoşten et al., 2012, TR | low | low | unlcear | low | low | low |
| 1. Ali et al., 2009, TR | low | low | Unclear | unclear | low | low |
| 1. Kumar et al., 2015, IN | low | low | high | high | low | low |
| S. Mukadder et al., 2015, TR | low | unclear | high | high | low | low |
| SY. Park et al., 2015, KP | low | unclear | high | high | low | low |
| S.Y.NG et al., 2007, SG | low | low | unclear | high | low | low |
| AB Suzanna et al., 2011, MY | low | unclear | high | high | low | low |
| AM López et al., 2008, ES | low | low | high | high | unclear | low |
| CS. Strydom et al., 2008, ZA | low | unclear | high | high | low | low |
| MZ. Ali et al., 2011, EG | low | unlcear | high | high | low | low |
| H. Francksen et al., 2009, DE | low | low | high | high | low | low |
| J. Brimacombe et al., 2002, AU | low | low | high | high | low | low |
| L. Gaitini et al., 2006, US | low | low | high | high | low | low |
| CJ. Chung et al., 2009, KP | low | low | high | high | low | low |
| RE. Galgon et al., 2011, US | low | unclear | unclear | unclear | low | low |
| S. Amini et al., 2010, IR | low | low | high | Unclear | low | low |
| K. Schebesta et al., 2010, AT | low | unclear | high | high | low | low |
| O. Akca et al., 2004, US | low | low | high | high | low | low |
| 1. Abualhassan et al., 2011, EG | low | unclear | high | high | low | low |
| AV Zundert et al., 2006, BE | low | low | high | high | low | low |
| K. EL-Radaideh et al., 2015, JO | low | low | high | high | low | low |
| YM Choi et al., 2010, KR | low | low | unclear | unclear | low | low |
| YC Woo et al., 2011, KR | low | low | high | high | low | low |
| LK Anand et al., 2016, IN | low | low | high | high | low | low |
| K Hayashi et al., 2012, JP | low | low | high | high | low | low |
| H. Francksen et al., 2007, DE | low | low | high | high | low | low |
| DA Fernández et al., 2009, ES | low | unclear | high | high | low | low |
| SK Mishra et al., 2015, IN | low | Unclear | high | high | low | low |
| S Taxak et al., 2013, IN | Low | unclear | high | high | low | low |
| YB Shi et al., 2013, CN | Low | Unclear | high | high | low | low |
| K Schebesta et al., 2014, AT | Low | unclear | high | high | low | low |
| AJ van Zundert et al., 2003, UK | Low | unclear | high | high | low | low |
| SK OH et al., 2012, KP | Low | unclear | high | high | low | low |
| EM Galvin et al., 2007, AN | unclear | unclear | high | high | low | low |
| A Turan et al., 2006, TR | Low | low | high | high | low | low |
| YK Kim et al., 2007, KP | Low | unclear | high | high | low | low |
| P.P. Lu et al., 2002, AU | Low | low | unlcear | unclear | low | low |
| T.Hosten et al., 2009, TR | Low | low | unclear | unclear | low | low |
| A.Das et al., 2014,IN | Low | low | unclear | unclear | low | low |
| U.Braun et al., 2002, DE | Low | low | unclear | unclear | low | low |
| T.M.Cook et al., 2005, UK | Low | low | unclear | unclear | low | low |
| Y. Li. et al., 2011, CN | Low | unclear | unclear | unclear | low | low |
| AR.I et al., 2012,MY | Low | unclear | unclear | unclear | low | low |
| B.Bein et al., 2005,DE | Low | unclear | unclear | unclear | low | low |
| J.Brimacombe et al., 2002, AT | Low | unclear | unclear | low | low | low |
| TM.Cook et al., 2003, JP | Low | unclear | unclear | unclear | low | low |
| E.Figueredo et al., 2003, ES | Low | low | unclear | unclear | low | low |
| LA. Gaitini et al., 2004, US | Low | low | Unclear | unclear | low | low |
| HV.Genzwuerker et al., 2007,DE | Low | low | unclear | unclear | low | low |
| J.F.Heuer et al., 2009,DE | Low | unclear | unclear | unclear | low | low |
| T.Kikuchi et al., 2008,JP | Low | low | unclear | unclear | low | low |
| G.Kini et al.,2014,IN | Low | unclear | unclear | unclear | low | low |
| M.Lange et al., 2007, DE | Low | unclear | unclear | unclear | low | low |
| A.Lee et al., 2009, SG | Low | unclear | unclear | unclear | low | low |
| S.Russo et al., 2012,DE | Low | unclear | unclear | unclear | low | low |
| A.Siddiqui et al.,2010,PK | Low | unclear | unclear | unclear | low | low |
| C.Thee et al. 2010,D | Low | unclear | unclear | unclear | low | low |
| H.Roth et al.2005,DE | Low | low | Unclear | unclear | low | low |
| T.S.Yildiz et al., 2007, TR | Low | low | unclear | unclear | low | low |
| K.Esa et al., 2011,MY | Low | low | unclear | unclear | low | low |
| N.S.Klaver et al., 2007,AN | Low | unclear | unclear | unclear | low | low |
| M.Wrobel et al., 2004, DE | Low | unclear | unclear | unclear | low | low |
| S.Mishra et al., 2015, IN | Low | unclear | unclear | unclear | low | low |
| S.Park et al., 2009,KP | Low | unclear | unclear | unclear | low | low |
| G.Liew et al., 2016, SG | Low | unclear | unclear | unclear | low | low |
| M.Somri et al., 2016,IL | Low | low | unclear | unclear | low | low |
| J.Belena et al., 2016,ES | Low | low | unclear | unclear | low | low |
| F.Zand et al., 2007, IR | Low | unclear | unclear | unclear | low | low |
| v. Zundert et al., 2020, AU  (accepted in 2019) | low | low | high | unclear | low | low |
| T. Wong et al., 2018, CA | Low | low | low | high | low | Low |
| B.Moser et al., 2018, CH | Low | low | low | high | low | Low |
| A Laurent et al., 2018, CH | low | low | low | high | low | low |
| Shariffuddin et al., 2016,MY. | low | low | low | high | low | low |
| Chang et al., 2019, R.O.K | low | unclear | unclear | unclear | low | low |
| Cross-over study | | | | | | |
| I.I.Shariffuddin et al., 2008, MY | Low | unclear | unclear | unclear | low | low |
| TM.Cook. et al., 2003, UK | Low | Unclear | unclear | unclear | low | low |
| TM.Cook. et al. 2005, UK | Low | low | unclear | unclear | low | low |
| TM.Cook. et al., 2002, UK | Low | low | unclear | unclear | low | low |
| M.J.Paech et al., 2005,AU | Low | low | unclear | unclear | low | low |
| C.Janakiraman et al., 2009, UK | Low | unclear | unclear | unclear | low | low |
| J.Brimacombe et al., 2004, AT | Low | low | unclear | unclear | low | low |
| J.Brimacombe et al., 2003, AT | Low | low | unclear | unclear | low | low |
| H.Tham et al., 2010, SG | low | unclear | unclear | unclear | low | low |
| V.Uppal et al.,2009,UK | Low | low | unclear | unclear | low | low |
| J.Brimacombe et al., 2001, AU | Low | unclear | high | high | low | low |
| J.Brimacombe et al., 2000, AU | Low | low | unclear | unclear | low | low |
| S.Eschertzhuber et al., 2009, AU | Low | low | unclear | unclear | low | low |
| G.Sudfir et al., 2007,UK | Low | unclear | unclear | unclear | low | low |
| U.Weber et al., 2011, AT | Low | low | unclear | unclear | low | low |

**Table S16: the list of excluded studies**

| **Study, Year, Country** | Reasons for exclusions |
| --- | --- |
| Donaldson et al., 2011, GB.^1^ | The demographic data is not clear illustrated in this study. |
| M. Ayedi et al., 2011, TN.^2^ | The measurements of primary outcomes and the demographic data have not been clear illustrated in this conference abstract. |
| DM. Miller et al., 2001, GB.^3^ | The measurements of OLP, first attempt insertion failure rate and postoperative sore throat rate were not clear. |
| J Kurola et al., 2006, FI.^4^ | The manipulator is paramedical students, not experienced operators. |
| TM. Abdel-Halim et al., 2014, MY.^5^ | The demographic data is not clear illustrated in this study. |
| H. Ocker et al., 2002, AT.^6^ | The definition of BMI is not clear illustrated. |
| C. Hein et al., 2006, AU.^7^ | The manipulator is medical students, not experienced operators. |
| V. Khazin et al., 2008, IL.^8^ | The measurements of OLP, first insertion failure rate and postoperative sore throat rate were not been clearly illustrated. |
| G. Venkateshwarlu, et al.,2015, IN^9^ | The measurements of our primary outcomes were not clearly illustrated and the BMI was not clear described. |
| M.G.E. Tan, et al., 2005, SG.^10^ | It is not clearly described patient BMI. |
| H.D. Durrani, et al., 2013, PK.^11^ | It is not clearly described the demographic data of patients. |
| I. Singh, et al., 2009, IN.^12^ | It is not clearly described the demographic data of patients. |
| A.Uday, et al., 2011, AU.^13^ | It is not clearly described patient’s BMI. |
| D.M. Miller, et al., 2003, GB.^14^ | It is not clearly described patient’s BMI. |
| EU. Neoh, et al., 2012, MY.^15^ | It is not clearly described patient’s BMI. |
| M. Agah, et al., 2006, IR.^16^ | It is not clearly described patient’s BMI. |
| K.S. Radhika, et al., 2016, IN.^17^ | It is not clearly described patient’s BMI. |
| Sk. Malhotra, et al., 2016, IN.^18^ | It is not clearly described patient’s BMI. |
| V. Mann et al., 2012, DE^19^ | It is not clearly described the OLP detailed data. |

**Reference for the excluded studies:**

1 Donaldson, W., Abraham, A., Deighan, M. & Michalek, P. I-gel vs. AuraOnce Laryngeal Mask for general anaesthesia with controlled ventilation in paralyzed patients. Biomedical papers of the Medical Faculty of the University Palacky, Olomouc, Czechoslovakia 155, 155-163 (2011).

2 Ayedi M., Z. J., Jarraya A., Bouaziz I., Kolsi K. The performance of the I-gel in comparison with the Laryngeal Mask Airway Classic. *Eur J Anaesthesiol.* **28**, 233 (2011).

3 Miller, D. M., Youkhana, I. & Pearce, A. C. The Laryngeal Mask and VBM Laryngeal Tube compared during spontaneous ventilation. A pilot study. *Eur J Anaesthesiol.* **18**, 593-598 (2001).

4 Kurola, J. et al. Comparison of airway management with the Intubating Laryngeal Mask, Laryngeal Tube and CobraPLA by paramedical students in anaesthetized patients. *Acta Anaesthesiol Scand.* **50,** 40-44 (2006).

5 Abdel-Halim, T. M., Abo El Enin, M. A., Elgoushi, M. M., Afifi, M. G. & Atwa, H. S. Comparative study between Air-Q and Intubating Laryngeal Mask Airway when used as conduit for fiber-optic.

*Egypt J Anaesth.* **30**, 107-113 (2014).

6 Ocker, H., Wenzel, V., Schmucker, P., Steinfath, M. & Dorges, V. A comparison of the Laryngeal Tube with the Laryngeal Mask Airway during routine surgical procedures. *Anesth Analg.* **95**, 1094-1097 (2002).

7 Hein, C., Owen, H. & Plummer, J. Randomized comparison of the SLIPA (Streamlined Liner of the Pharynx Airway) and the SS-LM (Soft Seal Laryngeal Mask) by medical students. *Emerg Med Australas.* **18**, 478-483 (2006).

8 Khazin, V. et al. Gastroesophageal regurgitation during anesthesia and controlled ventilation with six airway devices. J Clin Anesth. 20, 508-513 (2008).

9 G. Venkateshwarlu, C. S. Randomised Controlled Study Comparing Two Supraglottic Airway Devices: the I-gel and the Conventional Laryngeal Mask Airway in Anaesthetized Patients. *Journal of evidence based medicine and healthcare.* **2**, 8898-8902 (2015).

10 Tan, M. G., Chin, E. R., Kong, C. S., Chan, Y. H. & Ip-Yam, P. C. Comparison of the re-usable LMA Classic and two single-use Laryngeal Masks (LMA Unique and SoftSeal) in airway management by novice personnel. *Anaesth Intensive Care***. 33**, 739-743 (2005).

11 Durrani, D.H. et al. Comparison of LMA Classic and I-gel in anesthetized, spontaneously breathing patients during elective surgical procedures. *Anaesth Pain & Intensive Care*. **17**, 274-278 (2013).

12 Singh, I., Gupta, M. & Tandon, M. Comparison of Clinical Performance of I-gel with LMA-Proseal in Elective Surgeries. *Indian J Anaesth.* **53,** 302-305 (2009).

13 Ambi, U.S., Koppal, R., Joshi, C.& Iyer, H. LMA Classic and LMA Proseal: A Comparative Study in Paralyzed Anaesthetized Patients *Journal of Clinic Diagnostic Research*. **5**, 940-943 (2011).

14 Miller, D. M. & Light, D. Laboratory and clinical comparisons of the Streamlined Liner of the Pharynx Airway (SLIPA) with the Laryngeal Mask Airway. *Anaesthesia*. **58**, 136-142 (2003).

15 EU Neoh, Y. C. Comparison of the air-Q ILA and the LMA-Fastrach in airway management during general anaesthesia. *Southern African Journal of Anaesthesia and Analgesia.* **18,** 150-155 (2012).

16 Mahvash Agah, P. Y.& Fatemeh Roundneshin. Comparison between Classic Laryngeal Mask and Cobra Perilaryngeal Airway during Mechanical Ventilation. *Tanaffos* **5**, 13-19 (2006).

17 Radhika, K.S. et al. Assessment of suitability of I-gel and Laryngeal Mask Airway-supreme for controlled ventilation in anesthetized paralyzed patients : A prospective randomized trial. *Anesth Essays Res.* **10**, 88-93 (2016).

18 Malhotra, S. K., Bharath, K. V. & Saini, V. Comparison of success rate of intubation through Air-Q with ILMA using two different endotracheal tubes. *Indian J Anaesth*. **60**, 242-247 (2016).

19 Mann, V. et al. Einfluss von Kopfposition und Muskelrelaxierung auf die Effektivität der supraglottischen Atemwegssicherung. *Notfall + Rettungsmedizin.* **15**, 136-141 (2012).

**Table S17. Estimates of effects and quality ratings for comparison of different supraglottic airway devices to oropharyngeal leak pressure**

| comparisons | number of study | Direct evidence | | |  | Indirect evidence | |  | Network meta-analysis | |
| --- | --- | --- | --- | --- | --- | --- | --- | --- | --- | --- |
|  |  | Mean Difference  (95%CI) | I^2^ | Quality of evidence |  | Mean Difference  (95%CI) | Quality of evidence |  | Mean Difference  (95%CI) | Quality of evidence |
| U-LMA vs. C-LMA | -- | -- |  |  |  | -0.46 (-3.39 to 2.47) | A |  | -0.46 (-3.39 to 2.47) | A |
| Protector vs. C-LMA | -- | -- |  |  |  | 9.18 (5.60 to 12.75) | B^**^ |  | 9.18 (5.60 to 12.75) | B^**^ |
| AuraGain vs. C-LMA | -- | -- |  |  |  | 7.65 (3.63 to 11.67) | B^**^ |  | 7.65 (3.63 to 11.67) | B^**^ |
| P-LMA vs. C-LMA | 6 | 7.65 (7.18 to 8.12) | 0.97 | A |  | 6.01 (3.61 to 8.40) | A |  | 6.72 (5.05 to 8.40) | A |
| I-gel vs. C-LMA | 3 | 3.49 (2.01 to 4.97) | 0.00 | A |  | 4.18 (2.00 to 6.37) | A |  | 3.98 (2.14 to 5.82) | A |
| S-LMA vs. C-LMA | 1 | 8.50 (7.26 to 9.74) | -- | A |  | 4.43 (2.31 to 6.56) | A |  | 4.96 (2.96 to 6.95) | A |
| SLIPA vs. C-LMA | 1 | 0.00 (-1.82 to 1.82) | -- | A |  | 7.40 (4.01 to 10.79) | A |  | 5.39 (2.41 to 8.37) | A |
| Ambu-O vs. C-LMA | 2 | 1.63 (-0.26 to 3.51) | 0.86 | C ^††^ |  | 3.98 (-0.87 to 8.84) | C^††^ |  | 2.50 (-0.70 to 5.71) | C^††^ |
| Air-Q vs. C-LMA | -- | -- |  |  |  | 6.72 (0.49 to 12.95) | C^††^ |  | 6.72 (0.49 to 12.95) | C^††^ |
| SoftSeal vs. C-LMA | -- | -- |  |  |  | 4.55 (1.13 to 7.96) | B** |  | 4.55 (1.13 to 7.96) | B** |
| Solus vs. C-LMA | -- | -- |  |  |  | 1.41 (-2.87 to 5.69) | C^††^ |  | 1.41 (-2.87 to 5.69) | C^††^ |
| Cobra vs. C-LMA | 2 | 4.42 (2.85 to 5.99) | 0.00 | A |  | 5.80 (2.40 to 9.19) | A |  | 5.28 (2.66 to 7.90) | A |
| LT vs. C-LMA | 2 | 7.26 (5.30 to 9.22) | 0.00 | A |  | 4.39 (-0.34 to 9.13) | A |  | 5.94 (2.75 to 9.13) | A |
| LTS-II vs. C-LMA | -- | -- |  |  |  | 7.25 (4.40 to 10.10) | B^**^ |  | 7.25 (4.40 to 10.10) | B^**^ |
| LTS-D vs. C-LMA | -- | -- |  |  |  | 4.16 (-0.08 to 8.39) | C^††^ |  | 4.16 (-0.08 to 8.39) | C^††^ |
| LTS vs. C-LMA | -- | -- |  |  |  | 4.03 (-0.77 to 8.83) | C^††^ |  | 4.03 (-0.77 to 8.83) | C^††^ |
| Protector vs. U-LMA | -- | -- |  |  |  | 9.63 (5.44 to 13.83) | C^††^ |  | 9.63 (5.44 to 13.83) | C^††^ |
| AuraGain vs. U-LMA | -- | -- |  |  |  | 8.11 (3.51 to 12.70) | C^††^ |  | 8.11 (3.51 to 12.70) | C^††^ |
| P-LMA vs. U-LMA | -- | -- |  |  |  | 7.18 (4.33 to 10.03) | B^**^ |  | 7.18 (4.33 to 10.03) | B^**^ |
| I-gel vs. U-LMA | 2 | 10.03 (9.45 to 10.61) | 0.95 | B^**^ |  | 2.46 (-1.24 to 6.16) | B^**^ |  | 4.44 (1.66 to 7.23) | B^**^ |
| S-LMA vs. U-LMA | -- | -- |  |  |  | 5.41 (2.42 to 8.41) | B^**^ |  | 5.41 (2.42 to 8.41) | B^**^ |
| SLIPA vs. U-LMA | -- | -- |  |  |  | 5.85 (2.02 to 9.69) | B^**^ |  | 5.85 (2.02 to 9.69) | B^**^ |
| Ambu-O vs. U-LMA | 2 | 3.87 (1.98 to 5.75) | 0.00 | B^**^ |  | 1.95 (-2.97 to 6.88) | C^††^ |  | 2.96 (-0.27 to 6.19) | B^**^ |
| Air-Q vs. U-LMA | -- | -- |  |  |  | 7.18 (0.54 to 13.82) | C^††^ |  | 7.18 (0.54 to 13.82) | C^††^ |
| SoftSeal vs. U-LMA | 5 | 3.12 (2.39 to 3.86) | 0.90 | B^**^ |  | 12.22 (2.47 to 21.98) | C^††^ |  | 5.00 (2.47 to 7.54) | B |
| Solus vs. U-LMA | 1 | -1.35 (-4.12 to 1.42) | -- | B^**^ |  | 5.04 (-0.71 to 10.80) | C^††^ |  | 1.87 (-2.29 to 6.03) | C^††^ |
| Cobra vs. U-LMA | 2 | 5.29 (3.91 to 6.66) | 0.00 | B^**^ |  | 6.00 (1.81 to 10.19) | C^††^ |  | 5.73 (2.80 to 8.66) | C^††^ |
| LT vs. U-LMA | -- | -- |  |  |  | 6.40 (2.26 to 10.54) | C^††^ |  | 6.40 (2.26 to 10.54) | C^††^ |
| LTS-II vs. U-LMA | -- | -- |  |  |  | 7.71 (4.10 to 11.32) | C^††^ |  | 7.71 (4.10 to 11.32) | C^††^ |
| LTS-D vs. U-LMA | -- | -- |  |  |  | 4.61 (-0.15 to 9.38) | C^††^ |  | 4.61 (-0.15 to 9.38) | C^††^ |
| LTS vs. U-LMA | -- | -- |  |  |  | 4.49 (-0.84 to 9.82) | C^††^ |  | 4.49 (-0.84 to 9.82) | C^††^ |
| AuraGain vs. Protector | 1 | -1.90 (-4.48 to 0.68) | -- | B^**^ |  | -1.24 (-6.59 to 4.11) | B^**^ |  | -1.53 (-5.51 to 2.45) | B^**^ |
| P-LMA vs. Protector | -- | -- |  |  |  | -2.45 (-5.75 to 0.84) | B^**^ |  | -2.45 (-5.75 to 0.84) | B^**†^ |
| I-gel vs. Protector | 1 | -4.00 (-6.44 to -1.56) | -- | C^††^ |  | -5.68 (-9.52 to -1.85) | C^††^ |  | -5.19 (-8.41 to -1.98) | C^††^ |
| S-LMA vs. Protector | 2 | -4.27 (-5.39 to -3.16) | 0.00 | A |  | -3.71 (-8.42 to 1.00) | A |  | -4.22 (-7.29 to -1.15) | A |
| SLIPA vs. Protector | -- | -- |  |  |  | -3.78 (-7.99 to 0.43) | B^**^ |  | -3.78 (-7.99 to 0.43) | B^**^ |
| Ambu-O vs. Protector | -- | -- |  |  |  | -6.67 (-11.24 to -2.10) | C^††^ |  | -6.67 (-11.24 to -2.10) | C^††^ |
| Air-Q vs. Protector | -- | -- |  |  |  | -2.45 (-9.30 to 4.39) | C^††^ |  | -2.45 (-9.30 to 4.39) | C^††^ |
| SoftSeal vs. Protector | -- | -- |  |  |  | -4.63 (-9.22 to -0.04) | C^††^ |  | -4.63 (-9.22 to -0.04) | C^††^ |
| Solus vs. Protector | -- | -- |  |  |  | -7.76 (-12.95 to -2.58) | C^††^ |  | -7.76 (-12.95 to -2.58) | C^††^ |
| Cobra vs. Protector | -- | -- |  |  |  | -3.90 (-7.98 to 0.18) | C^††^ |  | -3.90 (-7.98 to 0.18) | C^††^ |
| LT vs. Protector | -- | -- |  |  |  | -3.24 (-7.78 to 1.31) | C^††^ |  | -3.24 (-7.78 to 1.31) | C^††^ |
| LTS-II vs. Protector | -- | -- |  |  |  | -1.93 (-5.97 to 2.12) | C^††^ |  | -1.93 (-5.97 to 2.12) | C^††^ |
| LTS-D vs. Protector | -- | -- |  |  |  | -5.02 (-9.89 to -0.15) | C^††^ |  | -5.02 (-9.89 to -0.15) | C^††^ |
| LTS vs. Protector | -- | -- |  |  |  | -5.15 (-10.72 to 0.43) | C^††^ |  | -5.15 (-10.72 to 0.43) | C^††^ |
| P-LMA vs. AuraGain | -- | -- |  |  |  | -0.93 (-4.69 to 2.84) | C^††^ |  | -0.93 (-4.69 to 2.84) | C^††^ |
| I-gel vs. AuraGain | -- | -- |  |  |  | -3.66 (-7.40 to 0.07) | C^††^ |  | -3.66 (-7.40 to 0.07) | C^††^ |
| S-LMA vs. AuraGain | 2 | -4.35 (-5.25 to -3.45) | 0.88 | B^**^ |  | -2.20 (-9.15 to 4.74) | C^††^ |  | -2.69 (-6.21 to 0.83) | C^††^ |
| SLIPA vs. AuraGain | -- | -- |  |  |  | -2.26 (-6.85 to 2.34) | C^††^ |  | -2.26 (-6.85 to 2.34) | C^††^ |
| Ambu-O vs. AuraGain | -- | -- |  |  |  | -5.15 (-10.08 to -0.21) | C^††^ |  | -5.15 (-10.08 to -0.21) | C^††^ |
| Air-Q vs. AuraGain | -- | -- |  |  |  | -0.93 (-8.01 to 6.16) | C^††^ |  | -0.93 (-8.01 to 6.16) | C^††^ |
| SoftSeal vs. AuraGain | -- | -- |  |  |  | -3.10 (-8.05 to 1.85) | C^††^ |  | -3.10 (-8.05 to 1.85) | C^††^ |
| Solus vs. AuraGain | -- | -- |  |  |  | -6.24 (-11.76 to -0.72) | C^††^ |  | -6.24 (-11.76 to -0.72) | C^††^ |
| Cobra vs. AuraGain | -- | -- |  |  |  | -2.37 (-6.86 to 2.11) | C^††^ |  | -2.37 (-6.86 to 2.11) | C^††^ |
| LT vs. AuraGain | -- | -- |  |  |  | -1.71 (-6.61 to 3.19) | C^††^ |  | -1.71 (-6.61 to 3.19) | C^††^ |
| LTS-II vs. AuraGain | -- | -- |  |  |  | -0.40 (-4.84 to 4.04) | C^††^ |  | -0.40 (-4.84 to 4.04) | C^††^ |
| LTS-D vs. AuraGain | -- | -- |  |  |  | -3.49 (-8.68 to 1.69) | C^††^ |  | -3.49 (-8.68 to 1.69) | C^††^ |
| LTS vs. AuraGain | -- | -- |  |  |  | -3.62 (-9.49 to 2.25) | C^††^ |  | -3.62 (-9.49 to 2.25) | C^††^ |
| I-gel vs. P-LMA | 14 | -2.90 (-3.37 to -2.42) | 0.86 | B^**^ |  | -2.84 (-5.10 to -0.58) | B^**^ |  | -2.74 (-4.02 to -1.46) | B^**^ |
| S-LMA vs. P-LMA | 11 | -1.11 (-1.65 to -0.58) | 0.74 | B^**^ |  | -2.52 (-4.95 to -0.08) | B^**^ |  | -1.77 (-3.19 to -0.35) | B^**^ |
| SLIPA vs. P-LMA | 3 | 0.20 (-1.15 to 1.55) | 0.56 | B^**^ |  | -3.42 (-7.59 to 0.74) | B^**^ |  | -1.33 (-4.08 to 1.42) | B^**^ |
| Ambu-O vs. P-LMA | -- | -- |  |  |  | -4.22 (-7.57 to -0.87) | C^††^ |  | -4.22 (-7.57 to -0.87) | C^††^ |
| Air-Q vs. P-LMA | 1 | 0.00 (-2.55 to 2.55) | -- | B^**^ |  | -13.50  (-8060.04 to 8033.05) | C^††^ |  | -0.00 (-6.00 to 6.00) | B^**^ |
| SoftSeal vs. P-LMA | -- | -- |  |  |  | -2.18 (-5.57 to 1.21) | B^**^ |  | -2.18 (-5.57 to 1.21) | B^**^ |
| Solus vs. P-LMA | -- | -- |  |  |  | -5.31 (-9.52 to -1.10) | B^**^ |  | -5.31 (-9.52 to -1.10) | B^**^ |
| Cobra vs. P-LMA | 2 | -0.20 (-1.40 to 1.00) | 0.80 | B^**^ |  | -2.71 (-6.01 to 0.60) | B^**^ |  | -1.45 (-4.01 to 1.12) | B^**^ |
| LT vs. P-LMA | 2 | -2.10 (-4.20 to 0.00) | 0.00 | A |  | 0.72 (-3.95 to 5.39) | A |  | -0.78 (-3.98 to 2.42) | A |
| LTS-II vs. P-LMA | 6 | 1.17 (0.20 to 2.14) | 0.90 | B^**^ |  | -4.64 (-16.31 to 7.04) | B^**^ |  | 0.53 (-1.84 to 2.89) | B^**^ |
| LTS-D vs. P-LMA | -- | -- |  |  |  | -2.57 (-6.57 to 1.43) | C^††^ |  | -2.57 (-6.57 to 1.43) | C^††^ |
| LTS vs. P-LMA | 2 | -2.41 (-4.68 to -0.15) | 0.13 | A |  | -13.61  (-5871.34 to 5844.12) | B^**^ |  | -2.69 (-7.19 to 1.81) | A |
| S-LMA vs. I-gel | 13 | 0.57 (0.04 to 1.10) | 0.83 | B^**^ |  | 1.76 (-0.73 to 4.25) | B^**^ |  | 0.97 (-0.40 to 2.34) | B^**^ |
| SLIPA vs. I-gel | 1 | 2.84 (1.35 to 4.33) | -- | A |  | 0.91 (-2.43 to 4.25) | A |  | 1.41 (-1.45 to 4.27) | A |
| Ambu-O vs. I-gel | -- | -- |  |  |  | -1.48 (-4.83 to 1.87) | B^**^ |  | -1.48 (-4.83 to 1.87) | B^**^ |
| Air-Q vs. I-gel | -- | -- |  |  |  | 2.74 (-3.40 to 8.87) | B^**^ |  | 2.74 (-3.40 to 8.87) | B^**^ |
| SoftSeal vs. I-gel | -- | -- |  |  |  | 0.56 (-2.79 to 3.92) | B^**^ |  | 0.56 (-2.79 to 3.92) | B^**^ |
| Solus vs. I-gel | 1 | 3.30 (0.60 to 6.01) | -- | B^**^ |  | -7.59 (-12.97 to -2.21) | B^**^ |  | -2.57 (-6.69 to 1.54) | B^**^ |
| Cobra vs. I-gel | -- | -- |  |  |  | 1.29 (-1.39 to 3.98) | B^**^ |  | 1.29 (-1.39 to 3.98) | B^**^ |
| LT vs. I-gel | -- | -- |  |  |  | 1.96 (-1.41 to 5.32) | B^**^ |  | 1.96 (-1.41 to 5.32) | B^**^ |
| LTS-II vs. I-gel | -- | -- |  |  |  | 3.26 (0.59 to 5.93) | B^**^ |  | 3.26 (0.59 to 5.93) | B^**^ |
| LTS-D vs. I-gel | 1 | -1.90 (-4.23 to 0.43) | -- | A |  | 1.88 (-3.42 to 7.19) | A |  | 0.17 (-3.76 to 4.10) | A |
| LTS vs. I-gel | -- | -- |  |  |  | 0.05 (-4.63 to 4.73) | C^††^ |  | 0.05 (-4.63 to 4.73) | C^††^ |
| SLIPA vs. S-LMA | -- | -- |  |  |  | 0.44 (-2.56 to 3.43) | C^††^ |  | 0.44 (-2.56 to 3.43) | C^††^ |
| Ambu-O vs. S-LMA | -- | -- |  |  |  | -2.45 (-5.95 to 1.04) | C^††^ |  | -2.45 (-5.95 to 1.04) | C^††^ |
| Air-Q vs. S-LMA | -- | -- |  |  |  | 1.77 (-4.40 to 7.93) | C^††^ |  | 1.77 (-4.40 to 7.93) | C^††^ |
| SoftSeal vs. S-LMA | -- | -- |  |  |  | -0.41 (-3.93 to 3.11) | B^**^ |  | -0.41 (-3.93 to 3.11) | B^**^ |
| Solus vs. S-LMA | -- | -- |  |  |  | -3.54 (-7.83 to 0.74) | C^††^ |  | -3.54 (-7.83 to 0.74) | C^††^ |
| Cobra vs. S-LMA | -- | -- |  |  |  | 0.32 (-2.50 to 3.14) | B^**^ |  | 0.32 (-2.50 to 3.14) | B^**^ |
| LT vs. S-LMA | -- | -- |  |  |  | 0.99 (-2.45 to 4.42) | B^**^ |  | 0.99 (-2.45 to 4.42) | B^**^ |
| LTS-II vs. S-LMA | -- | -- |  |  |  | 2.29 (-0.45 to 5.04) | B^**^ |  | 2.29 (-0.45 to 5.04) | B^**^ |
| LTS-D vs. S-LMA | 2 | -0.11 (-1.54 to 1.31) | 0.92 | B^**^ |  | -2.84 (-13.61 to 7.93) | B^**^ |  | -0.80 (-4.62 to 3.02) | B^**^ |
| LTS vs. S-LMA | -- | -- |  |  |  | -0.93 (-5.65 to 3.79) | C^††^ |  | -0.93 (-5.65 to 3.79) | C^††^ |
| Ambu-O vs. SLIPA | -- | -- |  |  |  | -2.89 (-7.08 to 1.30) | B^**^ |  | -2.89 (-7.08 to 1.30) | B^**^ |
| Air-Q vs. SLIPA | -- | -- |  |  |  | 1.33 (-5.27 to 7.93) | C^††^ |  | 1.33 (-5.27 to 7.93) | C^††^ |
| SoftSeal vs. SLIPA | -- | -- |  |  |  | -0.85 (-5.09 to 3.40) | B^**^ |  | -0.85 (-5.09 to 3.40) | B^**^ |
| Solus vs. SLIPA | -- | -- |  |  |  | -3.98 (-8.90 to 0.94) | C^††^ |  | -3.98 (-8.90 to 0.94) | C^††^ |
| Cobra vs. SLIPA | -- | -- |  |  |  | -0.12 (-3.77 to 3.54) | B^**^ |  | -0.12 (-3.77 to 3.54) | B^**^ |
| LT vs. SLIPA | -- | -- |  |  |  | 0.55 (-3.58 to 4.67) | C^††^ |  | 0.55 (-3.58 to 4.67) | C^††^ |
| LTS-II vs. SLIPA | -- | -- |  |  |  | 1.85 (-1.76 to 5.47) | B^**^ |  | 1.85 (-1.76 to 5.47) | B^**^ |
| LTS-D vs. SLIPA | -- | -- |  |  |  | -1.24 (-6.02 to 3.54) | C^††^ |  | -1.24 (-6.02 to 3.54) | C^††^ |
| LTS vs. SLIPA | -- | -- |  |  |  | -1.36 (-6.64 to 3.91) | C^††^ |  | -1.36 (-6.64 to 3.91) | C^††^ |
| Air-Q vs. Ambu-O | -- | -- |  |  |  | 4.22 (-2.65 to 11.09) | C^††^ |  | 4.22 (-2.65 to 11.09) | C^††^ |
| SoftSeal vs. Ambu-O | 2 | 2.66 (0.65 to 4.66) | 0.56 | A |  | 1.17 (-4.63 to 6.97) | A |  | 2.05 (-1.42 to 5.51) | A |
| Solus vs. Ambu-O | 1 | -4.08 (-6.55 to -1.61) | -- | A |  | 2.76 (-3.84 to 9.37) | A |  | -1.09 (-5.52 to 3.34) | A |
| Cobra vs. Ambu-O | -- | -- |  |  |  | 2.77 (-0.88 to 6.43) | B^**^ |  | 2.77 (-0.88 to 6.43) | B^**^ |
| LT vs. Ambu-O | -- | -- |  |  |  | 3.44 (-0.98 to 7.86) | C^††^ |  | 3.44 (-0.98 to 7.86) | C^††^ |
| LTS-II vs. Ambu-O | -- | -- |  |  |  | 4.75 (0.71 to 8.79) | C^††^ |  | 4.75 (0.71 to 8.79) | C^††^ |
| LTS-D vs. Ambu-O | -- | -- |  |  |  | 1.65 (-3.45 to 6.75) | C^††^ |  | 1.65 (-3.45 to 6.75) | C^††^ |
| LTS vs. Ambu-O | -- | -- |  |  |  | 1.53 (-4.08 to 7.14) | C^††^ |  | 1.53 (-4.08 to 7.14) | C^††^ |
| SoftSeal vs. Air-Q | -- | -- |  |  |  | -2.18 (-9.07 to 4.72) | C^††^ |  | -2.18 (-9.07 to 4.72) | C^††^ |
| Solus vs. Air-Q | -- | -- |  |  |  | -5.31 (-12.64 to 2.02) | C^††^ |  | -5.31 (-12.64 to 2.02) | C^††^ |
| Cobra vs. Air-Q | -- | -- |  |  |  | -1.45 (-7.97 to 5.08) | C^††^ |  | -1.45 (-7.97 to 5.08) | C^††^ |
| LT vs. Air-Q | -- | -- |  |  |  | -0.78 (-7.58 to 6.02) | C^††^ |  | -0.78 (-7.58 to 6.02) | C^††^ |
| LTS-II vs. Air-Q | -- | -- |  |  |  | 0.53 (-5.92 to 6.98) | C^††^ |  | 0.53 (-5.92 to 6.98) | C^††^ |
| LTS-D vs. Air-Q | -- | -- |  |  |  | -2.57 (-9.78 to 4.64) | C^††^ |  | -2.57 (-9.78 to 4.64) | C^††^ |
| LTS vs. Air-Q | -- | -- |  |  |  | -2.69 (-10.19 to 4.81) | C^††^ |  | -2.69 (-10.19 to 4.81) | C^††^ |
| Solus vs. SoftSeal | 1 | -8.16 (-10.68 to -5.64) | -- | C^††^ |  | 2.69 (-3.48 to 8.86) | C^††^ |  | -3.13 (-7.48 to 1.21) | C^††^ |
| Cobra vs. SoftSeal | 1 | -1.00 (-2.50 to 0.50) | -- | A |  | 1.72 (-2.56 to 5.99) | A |  | 0.73 (-2.67 to 4.13) | A |
| LT vs. SoftSeal | -- | -- |  |  |  | 1.39 (-3.12 to 5.91) | C^††^ |  | 1.39 (-3.12 to 5.91) | C^††^ |
| LTS-II vs. SoftSeal | -- | -- |  |  |  | 2.70 (-1.34 to 6.74) | C^††^ |  | 2.70 (-1.34 to 6.74) | C^††^ |
| LTS-D vs. SoftSeal | -- | -- |  |  |  | -0.39 (-5.51 to 4.72) | C^††^ |  | -0.39 (-5.51 to 4.72) | C^††^ |
| LTS vs. SoftSeal | -- | -- |  |  |  | -0.52 (-6.15 to 5.12) | C^††^ |  | -0.52 (-6.15 to 5.12) | C^††^ |
| Cobra vs. Solus | -- | -- |  |  |  | 3.86 (-0.65 to 8.38) | C^††^ |  | 3.86 (-0.65 to 8.38) | C^††^ |
| LT vs. Solus | -- | -- |  |  |  | 4.53 (-0.65 to 9.71) | C^††^ |  | 4.53 (-0.65 to 9.71) | C^††^ |
| LTS-II vs. Solus | -- | -- |  |  |  | 5.84 (1.06 to 10.62) | C^††^ |  | 5.84 (1.06 to 10.62) | C^††^ |
| LTS-D vs. Solus | -- | -- |  |  |  | 2.74 (-2.92 to 8.40) | C^††^ |  | 2.74 (-2.92 to 8.40) | C^††^ |
| LTS vs. Solus | -- | -- |  |  |  | 2.62 (-3.54 to 8.78) | C^††^ |  | 2.62 (-3.54 to 8.78) | C^††^ |
| LT vs. Cobra | -- | -- |  |  |  | 0.66 (-3.28 to 4.60) | C^††^ |  | 0.66 (-3.28 to 4.60) | C^††^ |
| LTS-II vs. Cobra | 1 | 1.50 (-0.37 to 3.37) | -- | A |  | 2.18 (-1.81 to 6.18) | A |  | 1.97 (-1.29 to 5.24) | A |
| LTS-D vs. Cobra | -- | -- |  |  |  | -1.12 (-5.79 to 3.55) | C^††^ |  | -1.12 (-5.79 to 3.55) | C^††^ |
| LTS vs. Cobra | -- | -- |  |  |  | -1.25 (-6.43 to 3.93) | C^††^ |  | -1.25 (-6.43 to 3.93) | C^††^ |
| LTS-II vs. LT | -- | -- |  |  |  | 1.31 (-2.65 to 5.27) | C^††^ |  | 1.31 (-2.65 to 5.27) | C^††^ |
| LTS-D vs. LT | -- | -- |  |  |  | -1.79 (-6.86 to 3.29) | C^††^ |  | -1.79 (-6.86 to 3.29) | C^††^ |
| LTS vs. LT | -- | -- |  |  |  | -1.91 (-7.43 to 3.61) | C^††^ |  | -1.91 (-7.43 to 3.61) | C^††^ |
| LTS-D vs. LTS-II | -- | -- |  |  |  | -3.09 (-7.73 to 1.54) | C^††^ |  | -3.09 (-7.73 to 1.54) | C^††^ |
| LTS vs. LTS-II | -- | -- |  |  |  | -3.22 (-8.30 to 1.87) | C^††^ |  | -3.22 (-8.30 to 1.87) | C^††^ |
| LTS vs. LTS-D | -- | -- |  |  |  | -0.13 (-6.15 to 5.89) | C^††^ |  | -0.13 (-6.15 to 5.89) | C^††^ |

* limitation (risk of bias), † heterogeneity ‡imprecision, ¶ inconsistency in side-splitting and down grading, p<0.05, ** contributing direct evidence of moderate quality, †† contributing evidence of low or very low quality, ‡‡ no first order indirect loop

Quality of evidence: A: high, B: moderate, C: low, D: very low

Note: C-LMA, LMA Classic; U-LMA, Unique LMA; Protector: LMA Protector Airway; AuraGain: Ambu AuraGain Disposable Laryngeal Mask; P-LMA, Proseal LMA; I-gel, i-gel supraglottic airway; S-LMA, Supreme LMA; SLIPA, Streamlined Liner of the Pharynx Airway; Ambu-O, Ambu AuraOnce; Air-Q, Air-Q Masked Laryngeal Airway.; SoftSeal, The Portex Soft Seal Laryngeal Mask; Solus, Solus Standard Laryngeal Mask Airway; Cobra, Cobra Perilaryngeal Airway; LT, Laryngeal Tube; LTSII, Laryngeal Tube Suction II; LTS-D, Laryngeal Tube Disposable; LTS, Laryngeal Tube Sonda.

**Table S18. Estimates of effects and quality ratings for comparison of different supraglottic airway devices to the risk of first-attempt failure insertion failure**

| comparisons | number of study | Direct evidence | | |  | Indirect evidence | |  | Network meta-analysis | |
| --- | --- | --- | --- | --- | --- | --- | --- | --- | --- | --- |
|  |  | Risk Ratio  (95%CI) | I^2^ | Quality of evidence |  | Risk Ratio  (95%CI) | Quality of evidence |  | Risk Ratio  (95%CI) | Quality of evidence |
| U-LMA vs. C-LMA | 1 | 1.49 (0.46 to 4.84) | -- | A |  | 0.67 (0.38 to 1.16) | A |  | 0.74 (0.44 to 1.24) | A |
| Protector vs. C-LMA | -- | -- |  |  |  | 0.90 (0.38 to 2.11) | B^**^ |  | 0.90 (0.38 to 2.11) | B^**^ |
| AuraGain vs. C-LMA | -- | -- |  |  |  | 0.71 (0.29 to 1.79) | B^**^ |  | 0.71 (0.29 to 1.79) | B^**^ |
| P-LMA vs. C-LMA | 9 | 1.68 (1.28 to 2.22) | 0.13 | A |  | 1.08 (0.68 to 1.71) | A |  | 1.34 (0.96 to 1.86) | A |
| I-gel vs. C-LMA | 6 | 1.47 (1.01 to 2.14) | 0.57 | A |  | 0.77 (0.49 to 1.21) | A |  | 0.99 (0.70 to 1.40) | A |
| S-LMA vs. C-LMA | 1 | 0.50 (0.17 to 1.51) | -- | A |  | 0.71 (0.46 to 1.10) | A |  | 0.69 (0.45 to 1.04) | A |
| SLIPA vs. C-LMA | 3 | 0.78 (0.40 to 1.52) | 0.01 | A |  | 2.28 (0.84 to 6.21) | A |  | 1.29 (0.67 to 2.51) | A |
| Ambu-O vs. C-LMA | 5 | 0.71 (0.42 to 1.18) | 0.13 | A |  | 0.67 (0.28 to 1.61) | A |  | 0.67 (0.40 to 1.13) | A |
| Air-Q vs. C-LMA | -- | -- |  |  |  | 0.56 (0.11 to 2.94) | A |  | 0.56 (0.11 to 2.94) | A |
| SoftSeal vs. C-LMA | 2 | 1.56 (0.65 to 3.79) | 0.00 | A |  | 0.92 (0.46 to 1.81) | A |  | 1.06 (0.60 to 1.88) | A |
| Solus vs. C-LMA | -- | -- |  |  |  | 1.22 (0.59 to 2.55) | A |  | 1.22 (0.59 to 2.55) | A |
| Cobra vs. C-LMA | 5 | 1.22 (0.63 to 2.37) | 0.58 | A |  | 1.59 (0.71 to 3.58) | A |  | 1.37 (0.77 to 2.42) | A |
| LT vs. C-LMA | 4 | 0.48 (0.33 to 0.71) | 0.34 | A |  | 2.72 (1.25 to 5.96) | A |  | 0.85 (0.52 to 1.38) | A |
| LTS-II vs. C-LMA | -- | -- |  |  |  | 1.16 (0.64 to 2.10) | B^**^ |  | 1.16 (0.64 to 2.10) | B^**^ |
| LTS-D vs. C-LMA | -- | -- |  |  |  | 1.40 (0.56 to 3.50) | B^**^ |  | 1.40 (0.56 to 3.50) | B^**^ |
| LTS vs. C-LMA | -- | -- |  |  |  | 2.40 (0.88 to 6.53) | B^**^ |  | 2.40 (0.88 to 6.53) | B^**^ |
| Protector vs. U-LMA | -- | -- |  |  |  | 1.21 (0.47 to 3.11) | B^**^ |  | 1.21 (0.47 to 3.11) | B^**^ |
| AuraGain vs. U-LMA | -- | -- |  |  |  | 0.96 (0.35 to 2.64) | B^**^ |  | 0.96 (0.35 to 2.64) | B^**^ |
| P-LMA vs. U-LMA | -- | -- |  |  |  | 1.80 (1.05 to 3.11) | B^**^ |  | 1.80 (1.05 to 3.11) | B^**^ |
| I-gel vs. U-LMA | 4 | 1.21 (0.64 to 2.29) | 0.00 | A |  | 1.43 (0.73 to 2.82) | A |  | 1.33 (0.80 to 2.23) | A |
| S-LMA vs. U-LMA | -- | -- |  |  |  | 0.93 (0.52 to 1.66) | B^**^ |  | 0.93 (0.52 to 1.66) | B^**^ |
| SLIPA vs. U-LMA | -- | -- |  |  |  | 1.75 (0.77 to 3.96) | B^**^ |  | 1.75 (0.77 to 3.96) | B^**^ |
| Ambu-O vs. U-LMA | 2 | 1.07 (0.57 to 2.02) | 0.00 | A |  | 0.79 (0.35 to 1.79) | A |  | 0.90 (0.49 to 1.64) | A |
| Air-Q vs. U-LMA | 1 | 0.49 (0.10 to 2.26) | -- |  |  | 4.19 (0.18 to 95.96) | C^††^ |  | 0.75 (0.15 to 3.81) | C^††^ |
| SoftSeal vs. U-LMA | 7 | 1.49 (1.06 to 2.10) | 0.32 | A |  | 1.07 (0.25 to 4.62) | A |  | 1.43 (0.89 to 2.30) | A |
| Solus vs. U-LMA | 1 | 2.30 (1.22 to 4.32) | -- | A |  | 1.16 (0.40 to 3.37) | A |  | 1.65 (0.80 to 3.42) | A |
| Cobra vs. U-LMA | 4 | 1.50 (0.83 to 2.71) | 0.01 | A |  | 2.45 (0.98 to 6.17) | A |  | 1.84 (1.03 to 3.30) | A |
| LT vs. U-LMA | -- | -- |  |  |  | 1.14 (0.57 to 2.28) | B^**^ |  | 1.14 (0.57 to 2.28) | B^**^ |
| LTS-II vs. U-LMA | -- | -- |  |  |  | 1.57 (0.76 to 3.26) | B^**^ |  | 1.57 (0.76 to 3.26) | B^**^ |
| LTS-D vs. U-LMA | -- | -- |  |  |  | 1.89 (0.70 to 5.13) | B^**^ |  | 1.89 (0.70 to 5.13) | B^**^ |
| LTS vs. U-LMA | -- | -- |  |  |  | 3.24 (1.09 to 9.65) | C^††^ |  | 3.24 (1.09 to 9.65) | C^††^ |
| AuraGain vs. Protector | 1 | 0.08 (0.01 to 0.57) | -- | B^**^ |  | 1.62 (0.51 to 5.19) | B^**^ |  | 0.80 (0.27 to 2.30) | B^**^ |
| P-LMA vs. Protector | -- | -- |  |  |  | 1.49 (0.66 to 3.38) | B^**^ |  | 1.49 (0.65 to 3.38) | B^**^ |
| I-gel vs. Protector | 1 | 0.80 (0.23 to 2.82) | -- | B^**^ |  | 1.24 (0.48 to 3.20) | B^**^ |  | 1.10 (0.49 to 2.47) | B^**^ |
| S-LMA vs. Protector | 2 | 1.48 (0.87 to 2.52) | 0.00 | A |  | 0.27 (0.07 to 0.96) | A |  | 0.77 (0.35 to 1.66) | A |
| SLIPA vs. Protector | -- | -- |  |  |  | 1.44 (0.51 to 4.08) | C^††^ |  | 1.44 (0.51 to 4.08) | C^††^ |
| Ambu-O vs. Protector | -- | -- |  |  |  | 0.74 (0.28 to 1.96) | B^**^ |  | 0.74 (0.28 to 1.96) | B^**^ |
| Air-Q vs. Protector | -- | -- |  |  |  | 0.62 (0.10 to 3.93) | C^††^ |  | 0.62 (0.10 to 3.93) | C^††^ |
| SoftSeal vs. Protector | -- | -- |  |  |  | 1.18 (0.44 to 3.16) | C^††^ |  | 1.18 (0.44 to 3.16) | C^††^ |
| Solus vs. Protector | -- | -- |  |  |  | 1.36 (0.47 to 3.98) | C^††^ |  | 1.36 (0.47 to 3.98) | C^††^ |
| Cobra vs. Protector | -- | -- |  |  |  | 1.52 (0.56 to 4.11) | C^††^ |  | 1.52 (0.56 to 4.11) | C^††^ |
| LT vs. Protector | -- | -- |  |  |  | 0.94 (0.36 to 2.44) | B^**^ |  | 0.94 (0.36 to 2.44) | B^**^ |
| LTS-II vs. Protector | -- | -- |  |  |  | 1.33 (0.52 to 3.36) | C^††^ |  | 1.29 (0.50 to 3.36) | C^††^ |
| LTS-D vs. Protector | -- | -- |  |  |  | 1.57 (0.49 to 4.94) | C^††^ |  | 1.56 (0.49 to 4.94) | C^††^ |
| LTS vs. Protector | -- | -- |  |  |  | 2.67 (0.76 to 9.36) | C^††^ |  | 2.67 (0.76 to 9.36) | C^††^ |
| P-LMA vs. AuraGain | -- | -- |  |  |  | 1.87 (0.77 to 4.54) | C^††^ |  | 1.87 (0.77 to 4.54) | C^††^ |
| I-gel vs. AuraGain | -- | -- |  |  |  | 1.38 (0.57 to 3.34) | C^††^ |  | 1.38 (0.57 to 3.34) | C^††^ |
| S-LMA vs. AuraGain | 2 | 0.68 (0.36 to 1.28) | 0.87 | B^**^ |  | 13.89 (1.40 to 137.57) | B^**^ |  | 0.96 (0.42 to 2.19) | B^**^ |
| SLIPA vs. AuraGain | -- | -- |  |  |  | 1.81 (0.60 to 5.44) | C^††^ |  | 1.81 (0.60 to 5.44) | C^††^ |
| Ambu-O vs. AuraGain | -- | -- |  |  |  | 0.93 (0.33 to 2.61) | B^**^ |  | 0.93 (0.33 to 2.61) | B^**^ |
| Air-Q vs. AuraGain | -- | -- |  |  |  | 0.78 (0.12 to 5.10) | C^††^ |  | 0.78 (0.12 to 5.10) | C^††^ |
| SoftSeal vs. AuraGain | -- | -- |  |  |  | 1.49 (0.52 to 4.22) | C^††^ |  | 1.49 (0.52 to 4.22) | C^††^ |
| Solus vs. AuraGain | -- | -- |  |  |  | 1.71 (0.56 to 5.27) | C^††^ |  | 1.71 (0.56 to 5.27) | C^††^ |
| Cobra vs. AuraGain | -- | -- |  |  |  | 1.91 (0.67 to 5.44) | C^††^ |  | 1.91 (0.67 to 5.44) | C^††^ |
| LT vs. AuraGain | -- | -- |  |  |  | 1.18 (0.43 to 3.27) | C^††^ |  | 1.18 (0.43 to 3.27) | C^††^ |
| LTS-II vs. AuraGain | -- | -- |  |  |  | 1.67 (0.57 to 4.48) | C^††^ |  | 1.63 (0.59 to 4.48) | C^††^ |
| LTS-D vs. AuraGain | -- | -- |  |  |  | 1.97 (0.60 to 6.47) | C^††^ |  | 1.96 (0.60 to 6.47) | C^††^ |
| LTS vs. AuraGain | -- | -- |  |  |  | 3.36 (0.92 to 12.28) | C^††^ |  | 3.36 (0.92 to 12.28) | C^††^ |
| I-gel vs. P-LMA | 18 | 0.71 (0.53 to 0.96) | 0.23 | A |  | 0.85 (0.53 to 1.37) | A |  | 0.74 (0.55 to 1.00) | A |
| S-LMA vs. P-LMA | 15 | 0.45 (0.32 to 0.64) | 0.00 | A |  | 0.67 (0.38 to 1.18) | A |  | 0.51 (0.37 to 0.72) | A |
| SLIPA vs. P-LMA | 3 | 2.24 (0.94 to 5.35) | 0.00 | A |  | 0.49 (0.20 to 1.20) | A |  | 0.97 (0.49 to 1.90) | A |
| Ambu-O vs. P-LMA | 1 | 0.50 (0.13 to 1.86) | -- | A |  | 0.50 (0.27 to 0.92) | A |  | 0.50 (0.28 to 0.89) | A |
| Air-Q vs. P-LMA | -- | -- |  |  |  | 0.42 (0.08 to 2.22) | B^**^ |  | 0.42 (0.08 to 2.22) | B^**^ |
| SoftSeal vs. P-LMA | -- | -- |  |  |  | 0.79 (0.43 to 1.46) | A |  | 0.79 (0.43 to 1.46) | A |
| Solus vs. P-LMA | -- | -- |  |  |  | 0.92 (0.44 to 1.93) | A |  | 0.92 (0.44 to 1.93) | A |
| Cobra vs. P-LMA | 2 | 1.93 (0.61 to 6.13) | 0.68 | A |  | 0.90 (0.46 to 1.77) | A |  | 1.02 (0.56 to 1.87) | A |
| LT vs. P-LMA | 2 | 1.52 (0.88 to 2.63) | 0.57 | A |  | 0.35 (0.19 to 0.65) | A |  | 0.63 (0.37 to 1.07) | A |
| LTS-II vs. P-LMA | 7 | 0.90 (0.66 to 1.21) | 0.00 | A |  | 1.07 (0.05 to 21.66) | A |  | 0.87 (0.53 to 1.42) | A |
| LTS-D vs. P-LMA | -- | -- |  |  |  | 1.05 (0.43 to 2.55) | B^**^ |  | 1.05 (0.43 to 2.55) | B^**^ |
| LTS vs. P-LMA | 2 | 1.84 (0.89 to 3.80) | 0.00 | A |  | 0.39 (0.00 to NA) | A |  | 1.80 (0.70 to 4.63) | A |
| S-LMA vs. I-gel | 16 | 0.81 (0.60 to 1.08) | 0.21 | A |  | 0.56 (0.31 to 1.02) | A |  | 0.70 (0.50 to 0.96) | A |
| SLIPA vs. I-gel | 1 | 0.11 (0.01 to 2.00) | -- | A |  | 1.51 (0.73 to 3.11) | A |  | 1.31 (0.65 to 2.64) | A |
| Ambu-O vs. I-gel | 1 | 0.43 (0.12 to 1.54) | -- | A |  | 0.74 (0.40 to 1.35) | A |  | 0.68 (0.38 to 1.20) | A |
| Air-Q vs. I-gel | -- | -- |  |  |  | 0.56 (0.11 to 2.99) | B^**^ |  | 0.56 (0.11 to 2.99) | B^**^ |
| SoftSeal vs. I-gel | -- | -- |  |  |  | 1.07 (0.60 to 1.94) | B^**^ |  | 1.07 (0.60 to 1.94) | B^**^ |
| Solus vs. I-gel | 1 | 0.86 (0.43 to 1.70) | -- | A |  | 1.63 (0.63 to 4.19) | A |  | 1.24 (0.61 to 2.53) | A |
| Cobra vs. I-gel | -- | -- |  |  |  | 1.38 (0.75 to 2.54) | B^**^ |  | 1.38 (0.75 to 2.54) | B^**^ |
| LT vs. I-gel | -- | -- |  |  |  | 0.85 (0.49 to 1.50) | B^**^ |  | 0.86 (0.49 to 1.50) | B^**^ |
| LTS-II vs. I-gel | -- | -- |  |  |  | 1.18 (0.66 to 2.09) | B^**^ |  | 1.18 (0.66 to 2.09) | B^**^ |
| LTS-D vs. I-gel | 1 | 1.81 (0.97 to 3.37) | -- | B^**^ |  | 0.82 (0.15 to 4.38) | B^**^ |  | 1.42 (0.60 to 3.37) | B^**^ |
| LTS vs. I-gel | -- | -- |  |  |  | 2.43 (0.90 to 6.55) | C^††^ |  | 2.43 (0.90 to 6.55) | C^††^ |
| SLIPA vs. S-LMA | -- | -- |  |  |  | 1.88 (0.90 to 3.91) | C^††^ |  | 1.88 (0.90 to 3.91) | C^††^ |
| Ambu-O vs. S-LMA | -- | -- |  |  |  | 0.97 (0.52 to 1.81) | B^**^ |  | 0.97 (0.52 to 1.81) | B^**^ |
| Air-Q vs. S-LMA | -- | -- |  |  |  | 0.81 (0.15 to 4.39) | C^††^ |  | 0.81 (0.15 to 4.39) | C^††^ |
| SoftSeal vs. S-LMA | -- | -- |  |  |  | 1.54 (0.81 to 2.94) | B^**^ |  | 1.54 (0.81 to 2.94) | B^**^ |
| Solus vs. S-LMA | -- | -- |  |  |  | 1.78 (0.83 to 3.85) | C^††^ |  | 1.78 (0.83 to 3.85) | C^††^ |
| Cobra vs. S-LMA | -- | -- |  |  |  | 1.99 (1.03 to 3.81) | C^††^ |  | 1.99 (1.03 to 3.81) | C^††^ |
| LT vs. S-LMA | -- | -- |  |  |  | 1.23 (0.68 to 2.24) | B^**^ |  | 1.23 (0.68 to 2.24) | B^**^ |
| LTS-II vs. S-LMA | -- | -- |  |  |  | 1.69 (0.93 to 3.06) | C^††^ |  | 1.69 (0.93 to 3.06) | C^††^ |
| LTS-D vs. S-LMA | 1 | 1.68 (0.93 to 3.06) | -- | C^††^ |  | 3.20 (0.59 to 17.45) | C^††^ |  | 2.04 (0.86 to 4.85) | C^††^ |
| LTS vs. S-LMA | -- | -- |  |  |  | 3.49 (1.28 to 9.53) | B^**^ |  | 3.49 (1.28 to 9.53) | B^**^ |
| Ambu-O vs. SLIPA | -- | -- |  |  |  | 0.52 (0.22 to 1.19) | A |  | 0.52 (0.22 to 1.19) | A |
| Air-Q vs. SLIPA | -- | -- |  |  |  | 0.43 (0.07 to 2.56) | A |  | 0.43 (0.07 to 2.56) | A |
| SoftSeal vs. SLIPA | -- | -- |  |  |  | 0.82 (0.35 to 1.94) | A |  | 0.82 (0.35 to 1.94) | A |
| Solus vs. SLIPA | -- | -- |  |  |  | 0.95 (0.36 to 2.49) | B^**^ |  | 0.95 (0.36 to 2.49) | B^**^ |
| Cobra vs. SLIPA | -- | -- |  |  |  | 1.06 (0.45 to 2.49) | B^**^ |  | 1.06 (0.45 to 2.49) | B^**^ |
| LT vs. SLIPA | -- | -- |  |  |  | 0.65 (0.29 to 1.46) | B^**^ |  | 0.65 (0.29 to 1.46) | B^**^ |
| LTS-II vs. SLIPA | -- | -- |  |  |  | 0.90 (0.39 to 2.07) | B^**^ |  | 0.90 (0.39 to 2.07) | B^**^ |
| LTS-D vs. SLIPA | -- | -- |  |  |  | 1.09 (0.36 to 3.25) | B^**^ |  | 1.09 (0.36 to 3.25) | B^**^ |
| LTS vs. SLIPA | -- | -- |  |  |  | 1.86 (0.58 to 5.93) | C^††^ |  | 1.86 (0.58 to 5.93) | C^††^ |
| Air-Q vs. Ambu-O | -- | -- |  |  |  | 0.84 (0.15 to 4.58) | C^††^ |  | 0.84 (0.15 to 4.58) | C^††^ |
| SoftSeal vs. Ambu-O | 2 | 1.39 (0.79 to 2.45) | 0.00 | A |  | 1.95 (0.78 to 4.88) | A |  | 1.59 (0.85 to 2.98) | A |
| Solus vs. Ambu-O | 1 | 2.09 (1.15 to 3.82) | -- | A |  | 1.49 (0.45 to 4.88) | A |  | 1.83 (0.85 to 3.95) | A |
| Cobra vs. Ambu-O | -- | -- |  |  |  | 2.04 (1.01 to 4.15) | A |  | 2.04 (1.01 to 4.15) | A |
| LT vs. Ambu-O | -- | -- |  |  |  | 1.27 (0.62 to 2.57) | A |  | 1.27 (0.62 to 2.57) | A |
| LTS-II vs. Ambu-O | -- | -- |  |  |  | 1.74 (0.82 to 3.71) | B^**^ |  | 1.74 (0.82 to 3.71) | B^**^ |
| LTS-D vs. Ambu-O | -- | -- |  |  |  | 2.10 (0.76 to 5.84) | C^††^ |  | 2.10 (0.76 to 5.84) | C^††^ |
| LTS vs. Ambu-O | -- | -- |  |  |  | 3.59 (1.19 to 10.88) | C^††^ |  | 3.59 (1.19 to 10.88) | C^††^ |
| SoftSeal vs. Air-Q | -- | -- |  |  |  | 1.90 (0.36 to 10.10) | C^††^ |  | 1.90 (0.36 to 10.10) | C^††^ |
| Solus vs. Air-Q | -- | -- |  |  |  | 2.20 (0.38 to 12.70) | C^††^ |  | 2.19 (0.38 to 12.70) | C^††^ |
| Cobra vs. Air-Q | 1 | 1.50 (0.31 to 7.30) | -- | C^††^ |  | 12.95 (0.60 to 278.66) | C^††^ |  | 2.45 (0.48 to 12.45) | C^††^ |
| LT vs. Air-Q | -- | -- |  |  |  | 1.52 (0.27 to 8.50) | C^††^ |  | 1.52 (0.27 to 8.50) | C^††^ |
| LTS-II vs. Air-Q | -- | -- |  |  |  | 2.08 (0.36 to 11.90) | C^††^ |  | 2.08 (0.36 to 11.90) | C^††^ |
| LTS-D vs. Air-Q | -- | -- |  |  |  | 2.52 (0.39 to 16.36) | C^††^ |  | 2.52 (0.39 to 16.36) | C^††^ |
| LTS vs. Air-Q | -- | -- |  |  |  | 4.30 (0.63 to 29.38) | C^††^ |  | 4.30 (0.63 to 29.38) | C^††^ |
| Solus vs. SoftSeal | 1 | 1.35 (0.83 to 2.21) | -- | A |  | 0.91 (0.29 to 2.92) | A |  | 1.15 (0.55 to 2.42) | A |
| Cobra vs. SoftSeal | 2 | 1.98 (0.85 to 4.58) | 0.77 | B^**^ |  | 0.90 (0.38 to 2.13) | B^**^ |  | 1.29 (0.67 to 2.46) | B^**^ |
| LT vs. SoftSeal | -- | -- |  |  |  | 0.80 (0.38 to 1.66) | B^**^ |  | 0.80 (0.38 to 1.66) | B^**^ |
| LTS-II vs. SoftSeal | -- | -- |  |  |  | 1.09 (0.50 to 2.38) | B^**^ |  | 1.09 (0.50 to 2.38) | B^**^ |
| LTS-D vs. SoftSeal | -- | -- |  |  |  | 1.32 (0.47 to 3.72) | B^**^ |  | 1.32 (0.47 to 3.72) | B^**^ |
| LTS vs. SoftSeal | -- | -- |  |  |  | 2.26 (0.73 to 6.95) | C^††^ |  | 2.26 (0.73 to 6.95) | C^††^ |
| Cobra vs. Solus | -- | -- |  |  |  | 1.12 (0.48 to 2.59) | A |  | 1.12 (0.48 to 2.59) | A |
| LT vs. Solus | -- | -- |  |  |  | 0.69 (0.29 to 1.64) | A |  | 0.69 (0.29 to 1.64) | A |
| LTS-II vs. Solus | -- | -- |  |  |  | 0.95 (0.39 to 2.32) | A |  | 0.95 (0.39 to 2.32) | A |
| LTS-D vs. Solus | -- | -- |  |  |  | 1.15 (0.38 to 3.50) | A |  | 1.15 (0.38 to 3.50) | A |
| LTS vs. Solus | -- | -- |  |  |  | 1.96 (0.59 to 6.54) | C^††^ |  | 1.96 (0.59 to 6.54) | C^††^ |
| LT vs. Cobra | 1 | 9.00 (1.21 to 66.70) | -- | C^††^ |  | 0.49 (0.22 to 1.10) | C^††^ |  | 0.62 (0.30 to 1.29) | C^††^ |
| LTS-II vs. Cobra | -- | -- |  |  |  | 0.85 (0.39 to 1.85) | B^**^ |  | 0.85 (0.39 to 1.85) | B^**^ |
| LTS-D vs. Cobra | -- | -- |  |  |  | 1.03 (0.36 to 2.91) | B^**^ |  | 1.03 (0.36 to 2.91) | B^**^ |
| LTS vs. Cobra | -- | -- |  |  |  | 1.76 (0.57 to 5.40) | C^††^ |  | 1.76 (0.57 to 5.40) | C^††^ |
| LTS-II vs. LT | -- | -- |  |  |  | 1.38 (0.67 to 2.82) | B^**^ |  | 1.38 (0.67 to 2.82) | B^**^ |
| LTS-D vs. LT | -- | -- |  |  |  | 1.66 (0.60 to 4.57) | C^††^ |  | 1.66 (0.60 to 4.57) | C^††^ |
| LTS vs. LT | -- | -- |  |  |  | 2.84 (0.96 to 8.38) | C^††^ |  | 2.84 (0.96 to 8.38) | C^††^ |
| LTS-D vs. LTS-II | 1 | 1.00 (0.07 to 15.26) | -- | B^**^ |  | 1.24 (0.43 to 3.57) | B^**^ |  | 1.21 (0.45 to 3.25) | B^**^ |
| LTS vs. LTS-II | -- | -- |  |  |  | 2.06 (0.71 to 6.00) | B^**^ |  | 2.06 (0.71 to 6.00) | B^**^ |
| LTS vs. LTS-D | -- | -- |  |  |  | 1.71 (0.47 to 6.25) | B^**^ |  | 1.71 (0.47 to 6.25) | B^**^ |

* limitation (risk of bias), † heterogeneity ‡imprecision, ¶ inconsistency in side-splitting and down grading, p<0.05, ** contributing direct evidence of moderate quality, †† contributing evidence of low or very low quality, ‡‡ no first order indirect loop

Quality of evidence: A: high, B: moderate, C: low, D: very low

Note: C-LMA, LMA Classic; U-LMA, Unique LMA; Protector, LMA Protector Airway; AuraGain: Ambu AuraGain Disposable Laryngeal Mask; P-LMA, Proseal LMA; I-gel, I-gel supraglottic airway; S-LMA, Supreme LMA; SLIPA, Streamlined Liner of the Pharynx Airway; Ambu-O, Ambu AuraOnce; Air-Q, Air-Q Masked Laryngeal Airway; SoftSeal, The Portex Soft Seal Laryngeal Mask; Solus, Solus Standard Laryngeal Mask Airway; Cobra, Cobra Perilaryngeal Airway; LT, Laryngeal Tube; LTSII, Laryngeal Tube Suction II; LTS-D, Laryngeal Tube Disposable; LTS, Laryngeal Tube Sonda.

**Table S19. Estimates of effects and quality ratings for comparison of different supraglottic airway devices to the postoperative sore throat rate**

| comparisons | number of study | Direct evidence | | |  | Indirect evidence | |  | Network meta-analysis | |
| --- | --- | --- | --- | --- | --- | --- | --- | --- | --- | --- |
|  |  | Risk Ratio  (95%CI) | I^2^ | Quality of evidence |  | Risk Ratio  (95%CI) | Quality of evidence |  | Risk Ratio  (95%CI) | Quality of evidence |
| U-LMA vs. C-LMA | -- | -- |  |  |  | 1.39 (0.43 to 4.53) | B^**^ |  | 1.39 (0.43 to 4.53) | B^**^ |
| Protector vs. C-LMA | -- | -- |  |  |  | 0.92 (0.33 to 2.55) | B^**^ |  | 0.92 (0.33 to 2.55) | B^**^ |
| AuraGain vs. C-LMA | -- | -- |  |  |  | 0.23 (0.05 to 1.11) | B^**^ |  | 0.23 (0.05 to 1.11) | B^**^ |
| P-LMA vs. C-LMA | 2 | 0.44 (0.25 to 0.77) | 0.00 | A |  | 1.57 (0.74 to 3.36) | A |  | 0.85 (0.46 to 1.59) | A |
| I-gel vs. C-LMA | 4 | 0.91 (0.59 to 1.41) | 0.55 | A |  | 0.20 (0.06 to 0.61) | A |  | 0.58 (0.31 to 1.08) | A |
| S-LMA vs. C-LMA | -- | -- |  |  |  | 0.99 (0.44 to 2.23) | B^**^ |  | 0.99 (0.44 to 2.23) | B^**^ |
| SLIPA vs. C-LMA | 2 | 0.44 (0.16 to 1.21) | 0.50 | A |  | 1.87 (0.48 to 7.23) | A |  | 0.80 (0.31 to 2.06) | A |
| Ambu-O vs. C-LMA | 2 | 0.45 (0.23 to 0.89) | 0.00 | A |  | 0.20 (0.01 to 4.64) | A |  | 0.39 (0.13 to 1.12) | A |
| SoftSeal vs. C-LMA | 1 | 0.75 (0.41 to 1.38) | -- | A |  | 1.71 (0.35 to 8.44) | C^††^ |  | 1.00 (0.39 to 2.54) | B^**^ |
| Solus vs. C-LMA | -- | -- |  |  |  | 1.36 (0.19 to 9.86) | C^††^ |  | 1.36 (0.19 to 9.86) | C^††^ |
| Cobra vs. C-LMA | 1 | 2.14 (1.02 to 4.50) | -- | C^††^ |  | 1.16 (0.22 to 6.12) | C^††^ |  | 1.72 (0.64 to 4.57) | C^††^ |
| LT vs. C-LMA | 2 | 1.37 (0.84 to 2.23) | 0.00 | A |  | 0.53 (0.11 to 2.64) | A |  | 1.12 (0.53 to 2.37) | A |
| LTS-II vs. C-LMA | -- | -- |  |  |  | 0.84 (0.32 to 2.20) | B^**^ |  | 0.84 (0.32 to 2.20) | B^**^ |
| LTS-D vs. C-LMA | -- | -- |  |  |  | 2.59 (0.08 to 81.40) | C^††^ |  | 2.59 (0.08 to 81.40) | C^††^ |
| LTS vs. C-LMA | -- | -- |  |  |  | 2.03 (0.36 to 11.52) | C^††^ |  | 2.03 (0.36 to 11.52) | C^††^ |
| Protector vs. U-LMA | -- | -- |  |  |  | 0.66 (0.14 to 3.14) | B^**^ |  | 0.66 (0.14 to 3.14) | B^**^ |
| AuraGain vs. U-LMA | -- | -- |  |  |  | 0.17 (0.02 to 1.18) | B^**^ |  | 0.17 (0.02 to 1.18) | B^**^ |
| P-LMA vs. U-LMA | -- | -- |  |  |  | 0.61 (0.16 to 2.32) | B^**^ |  | 0.61 (0.16 to 2.32) | B^**^ |
| I-gel vs. U-LMA | -- | -- |  |  |  | 0.41 (0.11 to 1.57) | B^**^ |  | 0.41 (0.11 to 1.57) | B^**^ |
| S-LMA vs. U-LMA | -- | -- |  |  |  | 0.71 (0.17 to 2.97) | B^**^ |  | 0.71 (0.17 to 2.97) | B^**^ |
| SLIPA vs. U-LMA | -- | -- |  |  |  | 0.58 (0.13 to 2.60) | B^**^ |  | 0.58 (0.13 to 2.60) | B^**^ |
| Ambu-O vs. U-LMA | 1 | 0.34 (0.01 to 8.14) | -- | B^**^ |  | 0.22 (0.04 to 1.21) | B^**^ |  | 0.28 (0.06 to 1.27) | B^**^ |
| SoftSeal vs. U-LMA | 2 | 0.89 (0.35 to 2.27) | 0.35 | A |  | 0.22 (0.02 to 2.06) | A |  | 0.72 (0.25 to 2.08) | A |
| Solus vs. U-LMA | -- | -- |  |  |  | 0.98 (0.10 to 9.78) | C^††^ |  | 0.98 (0.10 to 9.78) | C^††^ |
| Cobra vs. U-LMA | 2 | 1.06 (0.52 to 2.13) | 0.00 | A |  | 4.49 (0.35 to 56.97) | C^††^ |  | 1.23 (0.49 to 3.12) | B^**^ |
| LT vs. U-LMA | -- | -- |  |  |  | 0.81 (0.20 to 3.25) | B^**^ |  | 0.81 (0.20 to 3.25) | B^**^ |
| LTS-II vs. U-LMA | -- | -- |  |  |  | 0.60 (0.13 to 2.75) | B^**^ |  | 0.60 (0.13 to 2.75) | B^**^ |
| LTS-D vs. U-LMA | -- | -- |  |  |  | 1.86 (0.05 to 71.05) | C^††^ |  | 1.86 (0.05 to 71.05) | C^††^ |
| LTS vs. U-LMA | -- | -- |  |  |  | 1.45 (0.18 to 11.88) | C^††^ |  | 1.45 (0.18 to 11.88) | C^††^ |
| AuraGain vs. Protector | -- | -- |  |  |  | 0.25 (0.05 to 1.23) | B^**^ |  | 0.25 (0.05 to 1.23) | B^**^ |
| P-LMA vs. Protector | -- | -- |  |  |  | 0.93 (0.35 to 2.46) | B^**^ |  | 0.93 (0.35 to 2.46) | B^**^ |
| I-gel vs. Protector | 1 | 0.69 (0.35 to 1.34) | -- | A |  | 0.55 (0.14 to 2.10) | A |  | 0.63 (0.26 to 1.51) | A |
| S-LMA vs. Protector | 2 | 1.00 (0.46 to 2.20) | 0.00 | A |  | 1.25 (0.29 to 5.42) | A |  | 1.08 (0.46 to 2.50) | A |
| SLIPA vs. Protector | -- | -- |  |  |  | 0.87 (0.24 to 3.19) | B^**^ |  | 0.87 (0.24 to 3.19) | B^**^ |
| Ambu-O vs. Protector | -- | -- |  |  |  | 0.42 (0.10 to 1.85) | B^**^ |  | 0.42 (0.10 to 1.85) | B^**^ |
| SoftSeal vs. Protector | -- | -- |  |  |  | 1.09 (0.27 to 4.34) | B^**^ |  | 1.09 (0.27 to 4.34) | B^**^ |
| Solus vs. Protector | -- | -- |  |  |  | 1.48 (0.19 to 11.77) | C^††^ |  | 1.48 (0.19 to 11.77) | C^††^ |
| Cobra vs. Protector | -- | -- |  |  |  | 1.87 (0.45 to 7.68) | C^††^ |  | 1.87 (0.45 to 7.68) | C^††^ |
| LT vs. Protector | -- | -- |  |  |  | 1.22 (0.36 to 4.13) | B^**^ |  | 1.22 (0.36 to 4.13) | B^**^ |
| LTS-II vs. Protector | -- | -- |  |  |  | 0.91 (0.27 to 3.09) | B^**^ |  | 0.91 (0.27 to 3.09) | B^**^ |
| LTS-D vs. Protector | -- | -- |  |  |  | 2.82 (0.08 to 96.00) | C^††^ |  | 2.82 (0.08 to 96.00) | C^††^ |
| LTS vs. Protector | -- | -- |  |  |  | 2.20 (0.33 to 14.63) | C^††^ |  | 2.20 (0.33 to 14.63) | C^††^ |
| P-LMA vs. AuraGain | -- | -- |  |  |  | 3.64 (0.82 to 16.24) | C^††^ |  | 3.64 (0.82 to 16.24) | C^††^ |
| I-gel vs. AuraGain | -- | -- |  |  |  | 2.46 (0.55 to 11.01) | C^††^ |  | 2.46 (0.55 to 11.01) | C^††^ |
| S-LMA vs. AuraGain | 1 | 4.23 (1.73 to 10.38) | -- | C^††^ |  | 1.48 (0.00 to NA) | C^††^ |  | 4.23 (1.12 to 15.96) | C^††^ |
| SLIPA vs. AuraGain | -- | -- |  |  |  | 3.43 (0.60 to 19.56) | C^††^ |  | 3.43 (0.60 to 19.56) | C^††^ |
| Ambu-O vs. AuraGain | -- | -- |  |  |  | 1.66 (0.25 to 10.96) | C^††^ |  | 1.66 (0.25 to 10.96) | C^††^ |
| SoftSeal vs. AuraGain | -- | -- |  |  |  | 4.27 (0.70 to 26.17) | C^††^ |  | 4.27 (0.70 to 26.17) | C^††^ |
| Solus vs. AuraGain | -- | -- |  |  |  | 5.84 (0.53 to 64.34) | C^††^ |  | 5.84 (0.53 to 64.34) | C^††^ |
| Cobra vs. AuraGain | -- | -- |  |  |  | 7.34 (1.17 to 46.17) | C^††^ |  | 7.34 (1.17 to 46.17) | C^††^ |
| LT vs. AuraGain | -- | -- |  |  |  | 4.80 (0.89 to 25.91) | C^††^ |  | 4.80 (0.89 to 25.91) | C^††^ |
| LTS-II vs. AuraGain | -- | -- |  |  |  | 3.58 (0.67 to 18.97) | C^††^ |  | 3.58 (0.67 to 18.97) | C^††^ |
| LTS-D vs. AuraGain | -- | -- |  |  |  | 11.09 (0.27 to 451.17) | C^††^ |  | 11.09 (0.27 to 451.17) | C^††^ |
| LTS vs. AuraGain | -- | -- |  |  |  | 8.67 (0.95 to 78.67) | C^††^ |  | 8.67 (0.95 to 78.67) | C^††^ |
| I-gel vs. P-LMA | 5 | 0.40 (0.19 to 0.83) | 0.49 | A |  | 1.29 (0.53 to 3.15) | A |  | 0.68 (0.35 to 1.30) | A |
| S-LMA vs. P-LMA | 3 | 0.93 (0.58 to 1.50) | 0.00 | A |  | 1.95 (0.55 to 6.89) | A |  | 1.16 (0.58 to 2.32) | A |
| SLIPA vs. P-LMA | 1 | 1.81 (0.90 to 3.66) | -- | A |  | 0.38 (0.09 to 1.59) | A |  | 0.94 (0.37 to 2.43) | A |
| Ambu-O vs. P-LMA | -- | -- |  |  |  | 0.46 (0.13 to 1.58) | B^**^ |  | 0.46 (0.13 to 1.58) | B^**^ |
| SoftSeal vs. P-LMA | -- | -- |  |  |  | 1.17 (0.38 to 3.59) | B^**^ |  | 1.17 (0.38 to 3.59) | B^**^ |
| Solus vs. P-LMA | -- | -- |  |  |  | 1.60 (0.22 to 11.68) | C^††^ |  | 1.60 (0.22 to 11.68) | C^††^ |
| Cobra vs. P-LMA | -- | -- |  |  |  | 2.02 (0.63 to 6.46) | C^††^ |  | 2.02 (0.63 to 6.46) | C^††^ |
| LT vs. P-LMA | 1 | 0.71 (0.24 to 2.13) | -- | B^**^ |  | 1.84 (0.63 to 5.37) | B^**^ |  | 1.32 (0.55 to 3.16) | B^**^ |
| LTS-II vs. P-LMA | 4 | 1.11 (0.65 to 1.87) | 0.61 | B^**^ |  | 1.23 (0.00 to NA) | C^††^ |  | 0.98 (0.47 to 2.06) | B^**^ |
| LTS-D vs. P-LMA | -- | -- |  |  |  | 3.05 (0.10 to 90.50) | C^††^ |  | 3.05 (0.10 to 90.50) | C^††^ |
| LTS vs. P-LMA | 1 | 2.38 (0.65 to 8.68) | -- | C^††^ |  | 1.16 (0.00 to NA) | C^††^ |  | 2.38 (0.47 to 12.05) | C^††^ |
| S-LMA vs. I-gel | 5 | 2.81 (1.26 to 6.26) | 0.19 | A |  | 1.15 (0.39 to 3.36) | A |  | 1.72 (0.86 to 3.45) | A |
| SLIPA vs. I-gel | -- | -- |  |  |  | 1.40 (0.50 to 3.92) | A |  | 1.40 (0.50 to 3.92) | A |
| Ambu-O vs. I-gel | -- | -- |  |  |  | 0.68 (0.20 to 2.31) | A |  | 0.68 (0.20 to 2.31) | A |
| SoftSeal vs. I-gel | -- | -- |  |  |  | 1.74 (0.56 to 5.40) | B^**^ |  | 1.74 (0.56 to 5.40) | B^**^ |
| Solus vs. I-gel | 1 | 2.37 (0.48 to 11.74) | -- | C^††^ |  | 2.26 (0.00 to NA) | C^††^ |  | 2.37 (0.36 to 15.46) | C^††^ |
| Cobra vs. I-gel | -- | -- |  |  |  | 2.98 (0.93 to 9.55) | C^††^ |  | 2.98 (0.93 to 9.55) | C^††^ |
| LT vs. I-gel | -- | -- |  |  |  | 1.95 (0.77 to 4.94) | B^**^ |  | 1.95 (0.77 to 4.94) | B^**^ |
| LTS-II vs. I-gel | -- | -- |  |  |  | 1.45 (0.55 to 3.87) | B^**^ |  | 1.45 (0.55 to 3.87) | B^**^ |
| LTS-D vs. I-gel | -- | -- |  |  |  | 4.51 (0.14 to 142.14) | C^††^ |  | 4.51 (0.14 to 142.14) | C^††^ |
| LTS vs. I-gel | -- | -- |  |  |  | 3.52 (0.61 to 20.25) | C^††^ |  | 3.52 (0.61 to 20.25) | C^††^ |
| SLIPA vs. S-LMA | -- | -- |  |  |  | 0.81 (0.26 to 2.50) | B^**^ |  | 0.81 (0.26 to 2.50) | B^**^ |
| Ambu-O vs. S-LMA | -- | -- |  |  |  | 0.39 (0.10 to 1.50) | B^**^ |  | 0.39 (0.10 to 1.50) | B^**^ |
| SoftSeal vs. S-LMA | -- | -- |  |  |  | 1.01 (0.29 to 3.47) | B^**^ |  | 1.01 (0.29 to 3.47) | B^**^ |
| Solus vs. S-LMA | -- | -- |  |  |  | 1.38 (0.19 to 10.18) | C^††^ |  | 1.38 (0.19 to 10.18) | C^††^ |
| Cobra vs. S-LMA | -- | -- |  |  |  | 1.73 (0.49 to 6.19) | C^††^ |  | 1.73 (0.49 to 6.19) | C^††^ |
| LT vs. S-LMA | -- | -- |  |  |  | 1.13 (0.40 to 3.21) | B^**^ |  | 1.13 (0.40 to 3.21) | B^**^ |
| LTS-II vs. S-LMA | -- | -- |  |  |  | 0.85 (0.31 to 2.32) | B^**^ |  | 0.85 (0.31 to 2.32) | B^**^ |
| LTS-D vs. S-LMA | -- | -- |  |  |  | 2.62 (0.08 to 83.37) | C^††^ |  | 2.62 (0.08 to 83.37) | C^††^ |
| LTS vs. S-LMA | -- | -- |  |  |  | 2.05 (0.35 to 11.92) | C^††^ |  | 2.05 (0.35 to 11.92) | C^††^ |
| Ambu-O vs. SLIPA | -- | -- |  |  |  | 0.48 (0.12 to 1.98) | B^**^ |  | 0.48 (0.12 to 1.98) | B^**^ |
| SoftSeal vs. SLIPA | -- | -- |  |  |  | 1.24 (0.33 to 4.75) | B^**^ |  | 1.24 (0.33 to 4.75) | B^**^ |
| Solus vs. SLIPA | -- | -- |  |  |  | 1.70 (0.20 to 14.45) | C^††^ |  | 1.70 (0.20 to 14.45) | C^††^ |
| Cobra vs. SLIPA | -- | -- |  |  |  | 2.14 (0.55 to 8.30) | C^††^ |  | 2.14 (0.55 to 8.30) | C^††^ |
| LT vs. SLIPA | -- | -- |  |  |  | 1.40 (0.44 to 4.45) | B^**^ |  | 1.40 (0.44 to 4.45) | B^**^ |
| LTS-II vs. SLIPA | -- | -- |  |  |  | 1.04 (0.32 to 3.43) | B^**^ |  | 1.04 (0.32 to 3.43) | B^**^ |
| LTS-D vs. SLIPA | -- | -- |  |  |  | 3.23 (0.10 to 108.76) | C^††^ |  | 3.23 (0.10 to 108.76) | C^††^ |
| LTS vs. SLIPA | -- | -- |  |  |  | 2.52 (0.39 to 16.51) | B^**^ |  | 2.52 (0.39 to 16.51) | B^**^ |
| SoftSeal vs. Ambu-O | 1 | 7.18 (0.38 to 134.48) | -- | C^††^ |  | 1.80 (0.43 to 7.58) | C^††^ |  | 2.57 (0.66 to 9.95) | C^††^ |
| Solus vs. Ambu-O | -- | -- |  |  |  | 3.51 (0.37 to 33.04) | C^††^ |  | 3.51 (0.37 to 33.04) | C^††^ |
| Cobra vs. Ambu-O | -- | -- |  |  |  | 4.42 (1.09 to 17.88) | C^††^ |  | 4.42 (1.09 to 17.88) | C^††^ |
| LT vs. Ambu-O | -- | -- |  |  |  | 2.89 (0.79 to 10.58) | C^††^ |  | 2.89 (0.79 to 10.58) | C^††^ |
| LTS-II vs. Ambu-O | -- | -- |  |  |  | 2.15 (0.51 to 9.04) | C^††^ |  | 2.15 (0.51 to 9.04) | C^††^ |
| LTS-D vs. Ambu-O | -- | -- |  |  |  | 6.67 (0.18 to 245.87) | C^††^ |  | 6.67 (0.18 to 245.87) | C^††^ |
| LTS vs. Ambu-O | -- | -- |  |  |  | 5.21 (0.68 to 40.10) | C^††^ |  | 5.21 (0.68 to 40.10) | C^††^ |
| Solus vs. SoftSeal | -- | -- |  |  |  | 1.37 (0.15 to 12.21) | C^††^ |  | 1.37 (0.15 to 12.21) | C^††^ |
| Cobra vs. SoftSeal | 1 | 2.14 (0.77 to 5.94) | -- | A |  | 1.36 (0.31 to 5.89) | A |  | 1.72 (0.63 to 4.69) | A |
| LT vs. SoftSeal | -- | -- |  |  |  | 1.12 (0.34 to 3.73) | B^**^ |  | 1.12 (0.34 to 3.73) | B^**^ |
| LTS-II vs. SoftSeal | -- | -- |  |  |  | 0.84 (0.22 to 3.21) | B^**^ |  | 0.84 (0.22 to 3.21) | B^**^ |
| LTS-D vs. SoftSeal | -- | -- |  |  |  | 2.59 (0.07 to 92.28) | C^††^ |  | 2.59 (0.07 to 92.28) | C^††^ |
| LTS vs. SoftSeal | -- | -- |  |  |  | 2.03 (0.28 to 14.54) | C^††^ |  | 2.03 (0.28 to 14.54) | C^††^ |
| Cobra vs. Solus | -- | -- |  |  |  | 1.26 (0.14 to 11.41) | C^††^ |  | 1.26 (0.14 to 11.41) | C^††^ |
| LT vs. Solus | -- | -- |  |  |  | 0.82 (0.10 to 6.66) | B^**^ |  | 0.82 (0.10 to 6.66) | B^**^ |
| LTS-II vs. Solus | -- | -- |  |  |  | 0.61 (0.07 to 5.08) | B^**^ |  | 0.61 (0.07 to 5.08) | B^**^ |
| LTS-D vs. Solus | -- | -- |  |  |  | 1.90 (0.04 to 96.43) | C^††^ |  | 1.90 (0.04 to 96.43) | C^††^ |
| LTS vs. Solus | -- | -- |  |  |  | 1.48 (0.11 to 19.28) | C^††^ |  | 1.48 (0.11 to 19.28) | C^††^ |
| LT vs. Cobra | -- | -- |  |  |  | 0.65 (0.19 to 2.24) | B^**^ |  | 0.65 (0.19 to 2.24) | B^**^ |
| LTS-II vs. Cobra | -- | -- |  |  |  | 0.49 (0.12 to 1.93) | B^**^ |  | 0.49 (0.12 to 1.93) | B^**^ |
| LTS-D vs. Cobra | -- | -- |  |  |  | 1.51 (0.04 to 54.39) | C^††^ |  | 1.51 (0.04 to 54.39) | C^††^ |
| LTS vs. Cobra | -- | -- |  |  |  | 1.18 (0.16 to 8.69) | C^††^ |  | 1.18 (0.16 to 8.69) | C^††^ |
| LTS-II vs. LT | -- | -- |  |  |  | 0.74 (0.24 to 2.33) | B^**^ |  | 0.74 (0.24 to 2.33) | B^**^ |
| LTS-D vs. LT | -- | -- |  |  |  | 2.31 (0.07 to 76.48) | C^††^ |  | 2.31 (0.07 to 76.48) | C^††^ |
| LTS vs. LT | -- | -- |  |  |  | 1.81 (0.29 to 11.38) | C^††^ |  | 1.81 (0.29 to 11.38) | C^††^ |
| LTS-D vs. LTS-II | 1 | 3.10 (0.13 to 73.14) | -- | C^††^ |  | 1.07 (0.00 to NA) | C^††^ |  | 3.10 (0.11 to 84.81) | C^††^ |
| LTS vs. LTS-II | -- | -- |  |  |  | 2.42 (0.41 to 14.41) | C^††^ |  | 2.42 (0.41 to 14.41) | C^††^ |
| LTS vs. LTS-D | -- | -- |  |  |  | 0.78 (0.02 to 33.53) | C^††^ |  | 0.78 (0.02 to 33.53) | C^††^ |

* limitation (risk of bias), † heterogeneity ‡imprecision, ¶ inconsistency in side-splitting and down grading, p<0.05, ** contributing direct evidence of moderate quality, †† contributing evidence of low or very low quality, ‡‡ no first order indirect loop

Quality of evidence: A: high, B: moderate, C: low, D: very low

Note: C-LMA, LMA Classic; U-LMA, Unique LMA; Protector, LMA Protector Airway; AuraGain, Ambu AuraGain Disposable Laryngeal Mask; P-LMA, Proseal LMA; I-gel, I-gel supraglottic airway; S-LMA, Supreme LMA; SLIPA, Streamlined Liner of the Pharynx Airway; Ambu-O, Ambu AuraOnce; Air-Q, Air-Q Masked Laryngeal Airway.; SoftSeal, The Portex Soft Seal Laryngeal Mask; Solus, Solus Standard Laryngeal Mask Airway; Cobra, Cobra Perilaryngeal Airway; LT, Laryngeal Tube; LTSII, Laryngeal Tube Suction II; LTS-D, Laryngeal Tube Disposable; LTS, Laryngeal Tube Sonda.

**Table S20: Results for oropharyngeal leak pressure without neuromuscular blocking agents from network meta-analysis**

| C-LMA | -0.89  (-4.65,2.86) | **8.95**  **(3.98,13.93)** | **7.22**  **(2.23,12.21)** | **6.38**  **(3.84,8.92)** | **3.28**  **(0.62,5.94)** | **4.35**  **(1.40,7.30)** | 0.00  (-5.64,5.64) | 1.02  (-3.15,5.18) | 6.38  (-0.06,12.82) | 3.51  (-0.62,7.64) | -3.34  (-9.31,2.62) | **4.23**  **(0.81,7.65)** | **7.45**  **(1.53,13.37)** | **6.94**  **(2.07,11.81)** | 1.31  (-4.35,6.97) |
| --- | --- | --- | --- | --- | --- | --- | --- | --- | --- | --- | --- | --- | --- | --- | --- |
|  | U-LMA | **9.85**  **(3.95,15.74)** | **8.11**  **(2.20,14.02)** | **7.27**  **(3.12,11.43)** | **4.17**  **(0.12,8.23)** | **5.24**  **(0.92,9.56)** | 0.89  (-5.88,7.67) | 1.91  (-1.56,5.38) | **7.27**  **(0.04,14.50)** | **4.40**  **(1.88,6.93)** | -2.45  (-7.52,2.62) | **5.12**  **(1.81,8.44)** | **8.34**  **(1.33,15.36)** | **7.83**  **(1.96,13.71)** | 2.20  (-4.25,8.66) |
|  |  | Protector | -1.73  (-7.42,3.95) | -2.57  (-7.05,1.90) | **-5.67**  **(-10.09,-1.26)** | **-4.61**  **(-8.61,-0.60)** | **-8.95**  **(-16.48,-1.43)** | **-7.94**  **(-14.20,-1.67)** | -2.57  (-9.99,4.85) | -5.44  (-11.62,0.74) | **-12.30**  **(-19.84,-4.76)** | -4.72  (-10.58,1.13) | -1.50  (-9.24,6.23) | -2.01  (-8.12,4.09) | **-7.64**  **(-14.11,-1.17)** |
|  |  |  | AuraGain | -0.84  (-5.33,3.66) | -3.94  (-8.38,0.50) | -2.87  (-6.90,1.16) | -7.22  (-14.75,0.31) | -6.20  (-12.48,0.08) | -0.84  (-8.27,6.59) | -3.71  (-9.90,2.49) | **-10.56**  **(-18.11,-3.01)** | -2.99  (-8.86,2.88) | 0.23  (-7.52,7.98) | -0.28  (-6.40,5.84) | -5.91  (-12.39,0.58) |
|  |  |  |  | P-LMA | **-3.10**  **(-5.01,-1.20)** | **-2.03**  **(-4.03,-0.03)** | **-6.38**  **(-12.57,-0.19)** | **-5.36**  **(-10.00,-0.73)** | -0.00  (-5.92,5.92) | -2.87  (-7.41,1.67) | **-9.72**  **(-15.99,-3.46)** | -2.15  (-6.20,1.90) | 1.07  (-5.38,7.52) | 0.56  (-3.60,4.71) | -5.07  (-10.35,0.22) |
|  |  |  |  |  | i-gel | 1.07  (-0.79,2.93) | -3.28  (-9.51,2.96) | -2.26  (-6.86,2.33) | 3.10  (-3.11,9.32) | 0.23  (-4.24,4.70) | **-6.62**  **(-12.84,-0.41)** | 0.95  (-3.08,4.99) | 4.17  (-2.32,10.66) | 3.66  (-0.91,8.23) | -1.97  (-7.06,3.12) |
|  |  |  |  |  |  | S-LMA | -4.35  (-10.71,2.02) | -3.33  (-8.15,1.49) | 2.03  (-4.21,8.28) | -0.84  (-5.54,3.87) | **-7.69**  **(-14.08,-1.30)** | -0.12  (-4.39,4.16) | 3.10  (-3.52,9.72) | 2.59  (-2.02,7.20) | -3.04  (-8.12,2.05) |
|  |  |  |  |  |  |  | SLIPA | 1.02  (-5.99,8.03) | 6.38  (-2.18,14.94) | 3.51  (-3.48,10.50) | -3.34  (-11.55,4.87) | 4.23  (-2.36,10.83) | 7.45  (-0.73,15.63) | 6.94  (-0.51,14.39) | 1.31  (-6.68,9.30) |
|  |  |  |  |  |  |  |  | Ambu-O | 5.36  (-2.15,12.88) | 2.50  (-1.10,6.09) | -4.36  (-9.61,0.89) | 3.22  (-1.04,7.47) | 6.43  (-0.81,13.67) | 5.92  (-0.30,12.15) | 0.30  (-6.51,7.10) |
|  |  |  |  |  |  |  |  |  | Air-Q | -2.87  (-10.33,4.59) | **-9.72**  **(-18.34,-1.11)** | -2.15  (-9.32,5.02) | 1.07  (-7.68,9.82) | 0.56  (-6.67,7.79) | -5.07  (-13.00,2.87) |
|  |  |  |  |  |  |  |  |  |  | SoftSeal | **-6.85**  **(-11.94,-1.77)** | 0.72  (-2.98,4.42) | 3.94  (-3.28,11.16) | 3.43  (-2.73,9.58) | -2.20  (-8.92,4.52) |
|  |  |  |  |  |  |  |  |  |  |  | Solus | **7.58**  **(1.77,13.38)** | **10.79**  **(2.39,19.20)** | **10.28**  **(2.77,17.80)** | 4.66  (-3.34,12.65) |
|  |  |  |  |  |  |  |  |  |  |  |  | Cobra | 3.22  (-3.62,10.06) | 2.71  (-3.10,8.51) | -2.92  (-9.35,3.51) |
|  |  |  |  |  |  |  |  |  |  |  |  |  | LT | -0.51  (-8.18,7.16) | -6.14  (-14.33,2.06) |
|  |  |  |  |  |  |  |  |  |  |  |  |  |  | LTS-II | -5.63  (-12.35,1.10) |
|  |  |  |  |  |  |  |  |  |  |  |  |  |  |  | LTS-D |

Note: C-LMA, LMA Classic; U-LMA, Unique LMA; Protector, LMA Protector Airway; AuraGain, Ambu AuraGain Disposable Laryngeal Mask; P-LMA, Proseal LMA; I-gel, I-gel supraglottic airway; S-LMA, Supreme LMA; SLIPA, Streamlined Liner of the Pharynx Airway; Ambu-O, Ambu AuraOnce; Air-Q, Air-Q Masked Laryngeal Airway; SoftSeal, The Portex Soft Seal Laryngeal Mask; Solus, Solus Standard Laryngeal Mask Airway; Cobra, Cobra Perilaryngeal Airway; LT, Laryngeal Tube; LTSII, Laryngeal Tube Suction II; LTS-D, Laryngeal Tube Disposable; LTS, Laryngeal Tube Sonda.

**Table S21: Results for oropharyngeal leak pressure with neuromuscular blocking agents from network meta-analysis**

| C-LMA | -4.37  (-9.49,0.75) | **9.90**  **(4.26,15.54)** | **8.00**  **(0.39,15.60)** | **8.55**  **(6.26,10.84)** | **5.90**  **(3.36,8.43)** | **7.02**  **(4.46,9.58)** | **8.82**  **(5.38,12.25)** | 3.90  (-1.17,8.97) | **9.20**  **(3.44,14.96)** | **8.41**  **(4.47,12.34)** | **6.62**  **(3.18,10.07)** | **9.32**  **(5.97,12.67)** | **9.02**  **(3.59,14.44)** | **5.89**  **(1.37,10.41)** |
| --- | --- | --- | --- | --- | --- | --- | --- | --- | --- | --- | --- | --- | --- | --- |
|  | U-LMA | **14.27**  **(7.55,20.99)** | **12.37**  **(3.93,20.81)** | **12.92**  **(8.23,17.61)** | **10.27**  **(5.82,14.72)** | **11.39**  **(6.59,16.18)** | **13.19**  **(7.94,18.44)** | **8.27**  **(1.07,15.47)** | **13.57**  **(6.75,20.39)** | **12.78**  **(7.10,18.45)** | **10.99**  **(5.36,16.63)** | **13.69**  **(8.40,18.98)** | **13.39**  **(6.62,20.16)** | **10.26 (4.17,16.36)** |
|  |  | Protector | -1.90  (-7.01,3.21) | -1.35  (-6.60,3.91) | -4.00  (-9.04,1.04) | -2.88  (-8.23,2.46) | -1.08  (-6.83,4.67) | -6.00  (-13.58,1.58) | -0.70  (-7.92,6.52) | -1.49  (-7.64,4.66) | -3.28  (-9.39,2.84) | -0.58  (-6.37,5.22) | -0.88  (-8.06,6.29) | -4.01  (-10.55,2.53) |
|  |  |  | AuraGain | 0.55  (-6.77,7.88) | -2.10  (-9.27,5.07) | -0.98  (-8.38,6.41) | 0.82  (-6.87,8.51) | -4.10  (-13.24,5.04) | 1.20  (-7.64,10.04) | 0.41  (-7.59,8.40) | -1.38  (-9.34,6.59) | 1.32  (-6.40,9.05) | 1.02  (-7.79,9.82) | -2.11  (-10.40,6.19) |
|  |  |  |  | P-LMA | **-2.65**  **(-4.15,-1.15)** | -1.53  (-3.26,0.20) | 0.27  (-2.36,2.89) | -4.65  (-10.21,0.91) | 0.65  (-4.74,6.03) | -0.14  (-3.34,3.05) | -1.93  (-5.11,1.25) | 0.77  (-1.67,3.21) | 0.47  (-4.62,5.55) | -2.66  (-6.55,1.24) |
|  |  |  |  |  | i-gel | 1.12  (-0.68,2.92) | **2.92**  **(0.14,5.71)** | -2.00  (-7.66,3.67) | 3.30  (-1.87,8.47) | 2.51  (-1.02,6.04) | 0.72  (-2.74,4.19) | **3.42**  **(0.55,6.29)** | 3.12  (-1.99,8.23) | -0.01  (-4.18,4.17) |
|  |  |  |  |  |  | S-LMA | 1.80  (-1.24,4.84) | -3.12  (-8.79,2.56) | 2.18  (-3.29,7.66) | 1.39  (-2.25,5.02) | -0.39  (-3.93,3.15) | 2.30  (-0.69,5.29) | 2.00  (-2.78,6.78) | -1.12  (-5.38,3.14) |
|  |  |  |  |  |  |  | SLIPA | -4.92  (-11.04,1.20) | 0.38  (-5.49,6.25) | -0.41  (-4.55,3.72) | -2.20  (-6.30,1.91) | 0.50  (-3.08,4.09) | 0.20  (-5.47,5.87) | -2.93  (-7.62,1.77) |
|  |  |  |  |  |  |  |  | Ambu-O | 5.30  (-2.37,12.97) | 4.51  (-1.91,10.92) | 2.72  (-3.40,8.85) | 5.42  (-0.65,11.49) | 5.12  (-2.31,12.54) | 1.99  (-4.80,8.78) |
|  |  |  |  |  |  |  |  |  | Solus | -0.79  (-7.05,5.47) | -2.58  (-8.80,3.65) | 0.12  (-5.79,6.03) | -0.18  (-7.45,7.09) | -3.31  (-9.95,3.34) |
|  |  |  |  |  |  |  |  |  |  | Cobra | -1.78  (-6.29,2.72) | 0.91  (-2.64,4.47) | 0.61  (-5.40,6.62) | -2.51  (-7.55,2.53) |
|  |  |  |  |  |  |  |  |  |  |  | LT | 2.70  (-1.31,6.71) | 2.39  (-3.56,8.34) | -0.73  (-5.76,4.30) |
|  |  |  |  |  |  |  |  |  |  |  |  | LTS-II | -0.30  (-5.94,5.34) | -3.43  (-8.03,1.17) |
|  |  |  |  |  |  |  |  |  |  |  |  |  | LTS-D | -3.12  (-9.53,3.28) |
|  |  |  |  |  |  |  |  |  |  |  |  |  |  | LTS |

Note: C-LMA, LMA Classic; U-LMA, Unique LMA; Protector, LMA Protector Airway; AuraGain, Ambu AuraGain Disposable Laryngeal Mask; P-LMA, Proseal LMA; I-gel, I-gel supraglottic airway; S-LMA, Supreme LMA; SLIPA, Streamlined Liner of the Pharynx Airway; Ambu-O, Ambu AuraOnce; Air-Q, Air-Q Masked Laryngeal Airway.; SoftSeal, The Portex Soft Seal Laryngeal Mask; Solus, Solus Standard Laryngeal Mask Airway; Cobra, Cobra Perilaryngeal Airway; LT, Laryngeal Tube; LTSII, Laryngeal Tube Suction II; LTS-D, Laryngeal Tube Disposable; LTS, Laryngeal Tube Sonda.

**Table S22: Results for the risk of first-attempt insertion failure without neuromuscular blocking agents from network meta-analysis**

| C-LMA | 1.04  (0.59,1.82) | 0.44  (0.15,1.28) | 0.89  (0.32,2.51) | **1.75**  **(1.16,2.64)** | 1.24  (0.80,1.91) | 0.59  (0.33,1.05) | 0.84  (0.35,1.99) | 0.93  (0.51,1.69) | 0.77  (0.15,4.09) | 1.34  (0.73,2.47) | 2.01  (0.80,5.05) | 1.86  (0.97,3.58) | 1.28  (0.55,2.98) | 1.65  (0.77,3.56) | 1.49  (0.58,3.79) |
| --- | --- | --- | --- | --- | --- | --- | --- | --- | --- | --- | --- | --- | --- | --- | --- |
|  | U-LMA | 0.42  (0.13,1.33) | 0.86  (0.28,2.61) | 1.69  (0.91,3.14) | 1.19  (0.67,2.12) | 0.56  (0.28,1.15) | 0.81  (0.29,2.25) | 0.89  (0.48,1.66) | 0.74  (0.15,3.71) | 1.29  (0.80,2.08) | 1.93  (0.83,4.49) | 1.79  (0.97,3.30) | 1.23  (0.46,3.31) | 1.59  (0.65,3.90) | 1.43  (0.52,3.96) |
|  |  | Protector | 2.05  (0.58,7.19) | **4.03**  **(1.42,11.49)** | **2.85**  **(1.03,7.89)** | 1.35  (0.54,3.37) | 1.93  (0.48,7.67) | 2.13  (0.64,7.03) | 1.77  (0.25,12.54) | 3.08  (0.93,10.17) | **4.61**  **(1.16,18.30)** | **4.28**  **(1.26,14.61)** | 2.94  (0.78,11.04) | **3.80**  **(1.11,12.96)** | 3.41  (0.98,11.95) |
|  |  |  | AuraGain | 1.97  (0.72,5.36) | 1.39  (0.53,3.67) | 0.66  (0.28,1.55) | 0.94  (0.24,3.62) | 1.04  (0.33,3.29) | 0.87  (0.13,5.97) | 1.50  (0.48,4.75) | 2.25  (0.59,8.62) | 2.09  (0.64,6.85) | 1.43  (0.40,5.17) | 1.85  (0.57,6.07) | 1.66  (0.49,5.60) |
|  |  |  |  | P-LMA | 0.71  (0.47,1.06) | **0.33**  **(0.20,0.56)** | 0.48  (0.18,1.24) | 0.53  (0.27,1.04) | 0.44  (0.08,2.39) | 0.76  (0.38,1.52) | 1.14  (0.43,3.02) | 1.06  (0.51,2.21) | 0.73  (0.32,1.68) | 0.94  (0.49,1.81) | 0.85  (0.34,2.10) |
|  |  |  |  |  | I-gel | **0.47**  **(0.30,0.75)** | 0.68  (0.26,1.78) | 0.75  (0.38,1.45) | 0.62  (0.12,3.35) | 1.08  (0.56,2.08) | 1.62  (0.63,4.19) | 1.50  (0.73,3.08) | 1.03  (0.43,2.52) | 1.33  (0.62,2.85) | 1.20  (0.51,2.82) |
|  |  |  |  |  |  | S-LMA | 1.43  (0.50,4.05) | 1.58  (0.73,3.42) | 1.31  (0.23,7.42) | **2.28**  **(1.06,4.92)** | **3.42**  **(1.22,9.61)** | **3.17**  **(1.40,7.22)** | 2.18  (0.84,5.65) | **2.82**  **(1.24,6.39)** | **2.53**  **(1.07,5.96)** |
|  |  |  |  |  |  |  | SLIPA | 1.10  (0.39,3.16) | 0.92  (0.14,6.02) | 1.60  (0.55,4.61) | 2.39  (0.68,8.48) | 2.22  (0.75,6.56) | 1.53  (0.45,5.13) | 1.97  (0.62,6.27) | 1.77  (0.50,6.34) |
|  |  |  |  |  |  |  |  | Ambu-O | 0.83  (0.15,4.58) | 1.45  (0.76,2.75) | 2.17  (0.90,5.24) | 2.01  (0.93,4.37) | 1.38  (0.50,3.82) | 1.79  (0.70,4.55) | 1.61  (0.55,4.66) |
|  |  |  |  |  |  |  |  |  | Air-Q | 1.73  (0.33,9.10) | 2.60  (0.43,15.75) | 2.41  (0.48,12.13) | 1.66  (0.26,10.62) | 2.14  (0.35,13.12) | 1.92  (0.29,12.59) |
|  |  |  |  |  |  |  |  |  |  | SoftSeal | 1.50  (0.65,3.45) | 1.39  (0.71,2.75) | 0.96  (0.34,2.65) | 1.23  (0.48,3.16) | 1.11  (0.38,3.21) |
|  |  |  |  |  |  |  |  |  |  |  | Solus | 0.93  (0.35,2.50) | 0.64  (0.19,2.19) | 0.82  (0.26,2.64) | 0.74  (0.21,2.63) |
|  |  |  |  |  |  |  |  |  |  |  |  | Cobra | 0.69  (0.24,1.97) | 0.89  (0.33,2.36) | 0.80  (0.27,2.40) |
|  |  |  |  |  |  |  |  |  |  |  |  |  | LT | 1.29  (0.45,3.71) | 1.16  (0.35,3.89) |
|  |  |  |  |  |  |  |  |  |  |  |  |  |  | LTS-II | 0.90  (0.31,2.64) |
|  |  |  |  |  |  |  |  |  |  |  |  |  |  |  | LTS-D |

Note: C-LMA, LMA Classic; U-LMA, Unique LMA; Protector, LMA Protector Airway; AuraGain, Ambu AuraGain Disposable Laryngeal Mask; P-LMA, Proseal LMA; I-gel, I-gel supraglottic airway; S-LMA, Supreme LMA; SLIPA, Streamlined Liner of the Pharynx Airway; Ambu-O, Ambu AuraOnce; Air-Q, Air-Q Masked Laryngeal Airways.; SoftSeal, The Portex Soft Seal Laryngeal Mask; Solus, Solus Standard Laryngeal Mask Airways; Cobra, Cobra Perilaryngeal Airway; LT, Laryngeal Tube; LTSII, Laryngeal Tube Suction II; LTS-D, Laryngeal Tube Disposable; LTS, Laryngeal Tube Sonda.

**Table S23: Results for the risk of first-attempt insertion failure with neuromuscular blocking agents from network meta-analysis**

| C-LMA | 0.20  (0.01,3.79) | 0.73  (0.16,3.26) | **0.06**  **(0.00,0.73)** | 0.83  (0.48,1.45) | 0.59  (0.33,1.04) | 0.61  (0.33,1.10) | 1.37  (0.48,3.91) | 0.40  (0.15,1.09) | 1.95  (0.05,69.37) | 0.50  (0.17,1.44) | 0.65  (0.20,2.09) | 0.62  (0.37,1.04) | 0.63  (0.28,1.42) | 1.51  (0.56,4.11) |
| --- | --- | --- | --- | --- | --- | --- | --- | --- | --- | --- | --- | --- | --- | --- |
|  | U-LMA | 3.75  (0.15,93.90) | 0.29  (0.01,13.29) | 4.27  (0.23,80.85) | 3.00  (0.16,55.05) | 3.10  (0.16,58.83) | 7.04  (0.33,151.03) | 2.04  (0.09,46.58) | **10.00**  **(1.37,73.08)** | 2.57  (0.12,53.82) | 3.34  (0.15,76.08) | 3.18  (0.16,63.49) | 3.24  (0.16,65.16) | 7.76  (0.37,164.51) |
|  |  | Protector | **0.08**  **(0.01,0.61)** | 1.14  (0.27,4.83) | 0.80  (0.20,3.18) | 0.83  (0.19,3.52) | 1.88  (0.35,10.12) | 0.54  (0.09,3.28) | 2.67  (0.06,117.45) | 0.69  (0.13,3.53) | 0.89  (0.15,5.35) | 0.85  (0.18,4.00) | 0.86  (0.18,4.13) | 2.07  (0.39,10.92) |
|  |  |  | AuraGain | **14.82**  **(1.18,185.26)** | 10.40  (0.86,125.51) | 10.75  (0.86,134.86) | **24.41**  **(1.69,352.92)** | 7.07  (0.45,109.84) | 34.67  (0.46,2595.21) | 8.91  (0.63,125.31) | 11.57  (0.75,179.35) | 11.01  (0.83,146.76) | 11.24  (0.84,150.75) | **26.90**  **(1.89,383.74)** |
|  |  |  |  | P-LMA | 0.70  (0.46,1.07) | 0.73  (0.48,1.09) | 1.65  (0.68,4.02) | 0.48  (0.15,1.50) | 2.34  (0.07,81.43) | 0.60  (0.23,1.60) | 0.78  (0.26,2.33) | 0.74  (0.38,1.44) | 0.76  (0.42,1.37) | 1.82  (0.79,4.16) |
|  |  |  |  |  | I-gel | 1.03  (0.67,1.61) | 2.35  (0.89,6.17) | 0.68  (0.21,2.15) | 3.33  (0.10,113.12) | 0.86  (0.35,2.08) | 1.11  (0.35,3.49) | 1.06  (0.52,2.15) | 1.08  (0.52,2.25) | **2.59**  **(1.02,6.54)** |
|  |  |  |  |  |  | S-LMA | 2.27  (0.86,5.99) | 0.66  (0.20,2.11) | 3.22  (0.09,112.48) | 0.83  (0.31,2.23) | 1.08  (0.34,3.39) | 1.02  (0.50,2.10) | 1.05  (0.51,2.14) | **2.50**  **(1.00,6.28)** |
|  |  |  |  |  |  |  | SLIPA | 0.29  (0.07,1.23) | 1.42  (0.04,54.90) | 0.37  (0.10,1.35) | 0.47  (0.12,1.92) | 0.45  (0.15,1.37) | 0.46  (0.16,1.35) | 1.10  (0.33,3.71) |
|  |  |  |  |  |  |  |  | Ambu-O | 4.91  (0.12,200.01) | 1.26  (0.30,5.39) | 1.64  (0.35,7.57) | 1.56  (0.50,4.84) | 1.59  (0.44,5.78) | 3.81  (0.93,15.65) |
|  |  |  |  |  |  |  |  |  | SoftSeal | 0.26  (0.01,9.74) | 0.33  (0.01,13.58) | 0.32  (0.01,11.57) | 0.32  (0.01,11.87) | 0.78  (0.02,29.69) |
|  |  |  |  |  |  |  |  |  |  | Solus | 1.30  (0.31,5.51) | 1.24  (0.40,3.84) | 1.26  (0.40,3.98) | 3.02  (0.84,10.88) |
|  |  |  |  |  |  |  |  |  |  |  | Cobra | 0.95  (0.28,3.25) | 0.97  (0.28,3.40) | 2.32  (0.59,9.12) |
|  |  |  |  |  |  |  |  |  |  |  |  | LT | 1.02  (0.42,2.45) | 2.44  (0.84,7.07) |
|  |  |  |  |  |  |  |  |  |  |  |  |  | LTS-II | 2.39  (0.86,6.63) |
|  |  |  |  |  |  |  |  |  |  |  |  |  |  | LTS |

Note: C-LMA, LMA Classic; U-LMA, Unique LMA; Protector, LMA Protector Airway; AuraGain, Ambu AuraGain Disposable Laryngeal Mask; P-LMA, Proseal LMA; I-gel, I-gel supraglottic airway; S-LMA, Supreme LMA; SLIPA, Streamlined Liner of the Pharynx Airway; Ambu-O, Ambu AuraOnce; Air-Q, Air-Q Masked Laryngeal Airway.; SoftSeal, The Portex Soft Seal Laryngeal Mask; Solus, Solus Standard Laryngeal Mask Airway; Cobra, Cobra Perilaryngeal Airway; LT, Laryngeal Tube; LTSII, Laryngeal Tube Suction II; LTS-D, Laryngeal Tube Disposable; LTS, Laryngeal Tube Sonda.

**Table S24: Results for the postoperative sore throat rate without neuromuscular blocking agents from network meta-analysis**

| C-LMA | 1.03  (0.20,5.35) | 1.33  (0.28,6.34) | 0.32  (0.05,1.90) | 1.19  (0.45,3.14) | 0.80  (0.35,1.82) | 1.33  (0.45,3.98) | 0.38  (0.10,1.45) | 0.37  (0.12,1.17) | 0.86  (0.26,2.79) | 1.16  (0.20,6.58) | 1.49  (0.40,5.54) | 4.61  (0.13,168.34) |
| --- | --- | --- | --- | --- | --- | --- | --- | --- | --- | --- | --- | --- |
|  | U-LMA | 1.30  (0.13,12.53) | 0.31  (0.03,3.50) | 1.16  (0.17,7.85) | 0.78  (0.12,4.90) | 1.30  (0.18,9.37) | 0.37  (0.05,3.10) | 0.36  (0.05,2.36) | 0.83  (0.25,2.79) | 1.13  (0.40,3.17) | 1.44  (0.18,11.91) | 4.48  (0.09,234.39) |
|  |  | Protector | 0.24  (0.04,1.44) | 0.89  (0.23,3.53) | 0.60  (0.14,2.51) | 1.00  (0.33,3.04) | 0.29  (0.04,2.26) | 0.28  (0.04,1.94) | 0.64  (0.09,4.50) | 0.87  (0.08,8.96) | 1.11  (0.22,5.77) | 3.46  (0.08,144.11) |
|  |  |  | AuraGain | 3.78  (0.74,19.45) | 2.53  (0.47,13.70) | **4.23**  **(1.02,17.57)** | 1.22  (0.13,11.48) | 1.18  (0.14,9.99) | 2.71  (0.32,23.14) | 3.68  (0.30,44.70) | 4.72  (0.73,30.60) | 14.62  (0.32,677.26) |
|  |  |  |  | P-LMA | 0.67  (0.28,1.62) | 1.12  (0.50,2.51) | 0.32  (0.06,1.70) | 0.31  (0.07,1.42) | 0.72  (0.16,3.27) | 0.97  (0.13,7.11) | 1.25  (0.50,3.09) | 3.87  (0.12,124.19) |
|  |  |  |  |  | I-gel | 1.67  (0.67,4.15) | 0.48  (0.10,2.30) | 0.46  (0.11,1.91) | 1.07  (0.25,4.54) | 1.45  (0.21,9.94) | 1.86  (0.53,6.52) | 5.78  (0.16,206.25) |
|  |  |  |  |  |  | S-LMA | 0.29  (0.05,1.63) | 0.28  (0.06,1.37) | 0.64  (0.13,3.18) | 0.87  (0.11,6.77) | 1.11  (0.33,3.75) | 3.46  (0.10,121.68) |
|  |  |  |  |  |  |  | SLIPA | 0.96  (0.17,5.50) | 2.22  (0.37,13.24) | 3.01  (0.34,26.75) | 3.86  (0.59,25.24) | 11.98  (0.26,556.61) |
|  |  |  |  |  |  |  |  | Ambu-O | 2.31  (0.50,10.74) | 3.13  (0.44,22.33) | 4.01  (0.69,23.18) | 12.44  (0.28,545.09) |
|  |  |  |  |  |  |  |  |  | SoftSeal | 1.35  (0.36,5.04) | 1.74  (0.30,10.14) | 5.39  (0.12,237.25) |
|  |  |  |  |  |  |  |  |  |  | Cobra | 1.28  (0.15,11.34) | 3.98  (0.07,216.15) |
|  |  |  |  |  |  |  |  |  |  |  | LTS-II | 3.10  (0.11,88.25) |
|  |  |  |  |  |  |  |  |  |  |  |  | LTS-D |

Note: C-LMA, LMA Classic; U-LMA, Unique LMA; Protector, LMA® Protector Airway; AuraGain, Ambu AuraGain Disposable Laryngeal Mask; P-LMA, Proseal LMA; I-gel, I-gel supraglottic airway; S-LMA, Supreme LMA; SLIPA, Streamlined Liner of the Pharynx Airway; Ambu-O, Ambu AuraOnce; Air-Q, Air-Q Masked Laryngeal Airway; SoftSeal, The Portex Soft Seal Laryngeal Mask; Solus, Solus Standard Laryngeal Mask Airway; Cobra, Cobra Perilaryngeal Airway; LT, Laryngeal Tube; LTSII, Laryngeal Tube Suction II; LTS-D, Laryngeal Tube Disposable; LTS, Laryngeal Tube Sonda.

**Table S25: Results for the postoperative sore throat rate with neuromuscular blocking agents from network meta-analysis**

| C-LMA | 0.51  (0.10,2.72) | 0.87  (0.31,2.48) | 0.35  (0.11,1.17) | 1.41  (0.10,19.44) | 1.58  (0.33,7.61) | 0.84  (0.09,7.64) | 2.14  (0.65,7.10) | 1.13  (0.53,2.43) | 0.39  (0.06,2.39) | 2.08  (0.31,14.04) |
| --- | --- | --- | --- | --- | --- | --- | --- | --- | --- | --- |
|  | Protector | 1.70  (0.31,9.44) | 0.69  (0.22,2.18) | 2.75  (0.20,37.09) | 3.08  (0.39,24.61) | 1.63  (0.18,14.52) | 4.17  (0.54,32.42) | 2.20  (0.38,12.94) | 0.77  (0.08,7.34) | 4.05  (0.39,42.24) |
|  |  | P-LMA | 0.40  (0.11,1.43) | 1.62  (0.11,22.95) | 1.81  (0.56,5.86) | 0.96  (0.10,9.07) | 2.45  (0.50,11.99) | 1.30  (0.44,3.84) | 0.45  (0.10,1.96) | 2.38  (0.48,11.79) |
|  |  |  | i-gel | 4.00  (0.39,41.15) | 4.48  (0.80,25.19) | 2.37  (0.37,15.17) | **6.06**  **(1.11,33.01)** | 3.20  (0.84,12.25) | 1.11  (0.16,7.77) | 5.89  (0.77,45.30) |
|  |  |  |  | S-LMA | 1.12  (0.06,20.37) | 0.59  (0.03,11.67) | 1.52  (0.08,27.05) | 0.80  (0.05,11.79) | 0.28  (0.01,5.79) | 1.47  (0.07,32.61) |
|  |  |  |  |  | SLIPA | 0.53  (0.04,6.69) | 1.35  (0.19,9.76) | 0.72  (0.14,3.55) | 0.25  (0.04,1.64) | 1.32  (0.18,9.57) |
|  |  |  |  |  |  | Solus | 2.55  (0.21,31.52) | 1.35  (0.14,13.33) | 0.47  (0.03,6.89) | 2.48  (0.16,39.12) |
|  |  |  |  |  |  |  | Cobra | 0.53  (0.13,2.19) | 0.18  (0.02,1.60) | 0.97  (0.10,9.25) |
|  |  |  |  |  |  |  |  | LT | 0.35  (0.06,2.17) | 1.84  (0.27,12.71) |
|  |  |  |  |  |  |  |  |  | LTS-II | 5.28  (0.60,46.45) |
|  |  |  |  |  |  |  |  |  |  | LTS |

Note: C-LMA, LMA Classic; U-LMA, Unique LMA; Protector, LMA Protector Airway; AuraGain, Ambu AuraGain Disposable Laryngeal Mask; P-LMA, Proseal LMA; I-gel, I-gel supraglottic airway; S-LMA, Supreme LMA; SLIPA, Streamlined Liner of the Pharynx Airway; Ambu-O, Ambu AuraOnce; Air-Q, Air-Q Masked Laryngeal Airway.; SoftSeal, The Portex Soft Seal Laryngeal Mask; Solus, Solus Standard Laryngeal Mask Airway; Cobra, Cobra Perilaryngeal Airway; LT, Laryngeal Tube; LTSII, Laryngeal Tube Suction II; LTS-D, Laryngeal Tube Disposable; LTS, Laryngeal Tube Sonda.

**Table 26:** Search strategy

| **Pubmed search:** |
| --- |
| ((I-gel) OR (ProSeal LMA) OR (Classic LMA) OR (Supreme LMA) OR (AuraOnce) OR (Ambu LMA) OR (Unique LMA) OR (Streamlined Liner of the Pharynx Airway) OR (Solus LMA) OR (Portex Soft Seal LMA) OR (Air-Q LMA) OR (Cobra Perilaryngeal Airway) OR (Laryngeal Tube) OR (Laryngeal Tube Suction) OR (Laryngeal Tube Disposable) OR (Laryngeal Tube Sonda) OR (Ambu AuraGain Disposable Laryngeal Mask) OR (LMA Protector Airway))  AND  ((oropharyngeal leak pressure) OR (overall insertion failure during the induction) OR (first-attempt insertion failure) OR (insertion failure at first attempt) OR (failure of the device during the maintenance) OR (improper ventilation during the maintenance) OR (ease of insertion) OR (hypoxia) OR (complications) OR (sore throat) OR (pulmonary aspiration) OR (aspiration)) |
| **Embase search** |
| ((I-gel) OR (ProSeal LMA) OR (Classic LMA) OR (Supreme LMA) OR (AuraOnce) OR (Ambu LMA) OR (Unique LMA) OR (Streamlined Liner of the Pharynx Airway) OR (Solus LMA) OR (Portex Soft Seal LMA) OR (Air-Q LMA) OR (Cobra Perilaryngeal Airway) OR (Laryngeal Tube) OR (Laryngeal Tube Suction) OR (Laryngeal Tube Disposable) OR (Laryngeal Tube Sonda) OR (Ambu AuraGain Disposable Laryngeal Mask) OR (LMA Protector Airway))  AND  ((oropharyngeal leak pressure) OR (overall insertion failure during the induction) OR (first-attempt insertion failure) OR (insertion failure at first attempt) OR (failure of the device during the maintenance) OR (improper ventilation during the maintenance) OR (ease of insertion) OR (hypoxia) OR (complications) OR (sore throat) OR (pulmonary aspiration) OR (aspiration)) |
| **Cochrane clinical trial search** |
| ((I-gel) OR (ProSeal LMA) OR (Classic LMA) OR (Supreme LMA) OR (AuraOnce) OR (Ambu LMA) OR (Unique LMA) OR (Streamlined Liner of the Pharynx Airway) OR (Solus LMA) OR (Portex Soft Seal LMA) OR (Air-Q LMA) OR (Cobra Perilaryngeal Airway) OR (Laryngeal Tube) OR (Laryngeal Tube Suction) OR (Laryngeal Tube Disposable) OR (Laryngeal Tube Sonda) OR (Ambu AuraGain Disposable Laryngeal Mask) OR (LMA Protector Airway))  AND  ((oropharyngeal leak pressure) OR (overall insertion failure during the induction) OR (first-attempt insertion failure) OR (insertion failure at first attempt) OR (failure of the device during the maintenance) OR (improper ventilation during the maintenance) OR (ease of insertion) OR (hypoxia) OR (complications) OR (sore throat) OR (pulmonary aspiration) OR (aspiration)) |
