# Supplementary material for: Comparison of the efficacy of supraglottic airway devices in low-risk adult patients: a network meta-analysis and systematic review
Source: Sci Rep. 2021 Jul 23;11:15074. doi: 10.1038/s41598-021-94114-7 (PMC8302760; doi:10.1038/s41598-021-94114-7)

**Comparison of the Efficacy of Supraglottic Airway Devices in Low-Risk Adult Patients: A Network Meta-analysis and Systematic Review**

Chih-Jun Lai^†1,2^, Yi-Chun Yeh^†1,3^, Yu-Kang Tu*****^1,3,4^, Ya Jung Cheng^5,6^, Chih-Min Liu^1^, Shou-Zen Fan*****^2,6^

Short title: Network meta-analysis of supraglottic airways

†Both authors are contributed equally to this work

* Corresponding author: Yu-Kang Tu and Shou-Zen Fan

Affiliation:

1. Institute of Epidemiology and Preventive Medicine, National Taiwan University

2. Department of Anesthesiology, National Taiwan University Hospital

3. Department of Medical Research, National Taiwan University Hospital

4. Department of Dentistry, National Taiwan University Hospital and School of Dentistry, National Taiwan University

5. Department of Anesthesiology, National Taiwan University Cancer Center

6. Department of Anesthesiology, College of Medicine, National Taiwan University

*Corresponding author: Yu-Kang Tu, DDS, MSc, PhD

Address: Institute of Epidemiology and Preventive Medicine, College of Public Health, National Taiwan University, No. 17, Xu-Zhou Road, Taipei 100, Taiwan

Telephone: +88633668039;

Fax: 886-2-2351-1955

E-mail: yukangtu@ntu.edu.tw

*Corresponding author: Shou-Zen, Fan, MD, PhD

Address: Department of Anesthesiology, National Taiwan University Hospital.

No 7 Zhung Shan S. Road, Taipei, Taiwan

Telephone: +886223123456-65410;

Fax: +886227044688

E-mail: shouzen@gmail.com

**Supplementary figure**

Figure S1: Forest plots of network meta-analysis for oropharyngeal leak pressure….3

Figure S2: Forest plots of network meta-analysis for the risk of first-attempt insertion failure………………………………….……………………………………………..12

Figure S3: Forest plots of network meta-analysis for the postoperative sore throat rate………...………………………………………………………………………….21

Figure S4: Transitivity analysis of the use of neuromuscular blocking agents…………………………………………………………………………………29

Figure S5: Forest plots of node-splitting model for oropharyngeal leak pressure…...30

Figure S6: Forest plots of node-splitting model for the risk of first-attempt insertion failure……………….………………………………………………………………..31

Figure S7: Forest plots of node-splitting model for the postoperative sore throat rate……..……………………………………………………………………………..32

Figure S8: The publication bias assessment of oropharyngeal leak pressure……......33

Figure S9: The publication bias assessment of the risk of first-attempt insertion failure…………………………………………………….…………………………..34

Figure S10: The publication bias assessment of postoperative sore throat rate…...…35

Figure S11: The PRISMA Flow Diagram…..……………………………………......36

**Figure S1: Forest plots of network meta-analysis for oropharyngeal leak pressure**

|  |
| --- |
|  |
|  |
|  |
|  |
|  |
|  |
|  |
|  |
|  |
|  |
|  |
|  |
|  |
|  |
|  |
|  |
|  |
|  |
|  |
|  |
|  |
|  |
|  |
|  |
|  |
|  |
|  |
|  |
|  |

**Figure S2: Forest plots of network meta-analysis for the risk of first-attempt insertion failure**

|  |
| --- |
|  |
|  |
|  |
|  |
|  |
|  |
|  |
|  |
|  |
|  |
|  |
|  |
|  |
|  |
|  |
|  |
|  |
|  |
|  |

**Figure S3: Forest plots of network meta-analysis for the postoperative sore throat rate**

|  |
| --- |
|  |
|  |
|  |
|  |
|  |
|  |
|  |
|  |
|  |
|  |
|  |
|  |
|  |
|  |
|  |
|  |
|  |
|  |
|  |
|  |
|  |
|  |
|  |
|  |
|  |
|  |
|  |
|  |
|  |
|  |

**Figure S4: The transitivity analysis of neuromuscular blocking agents**

**
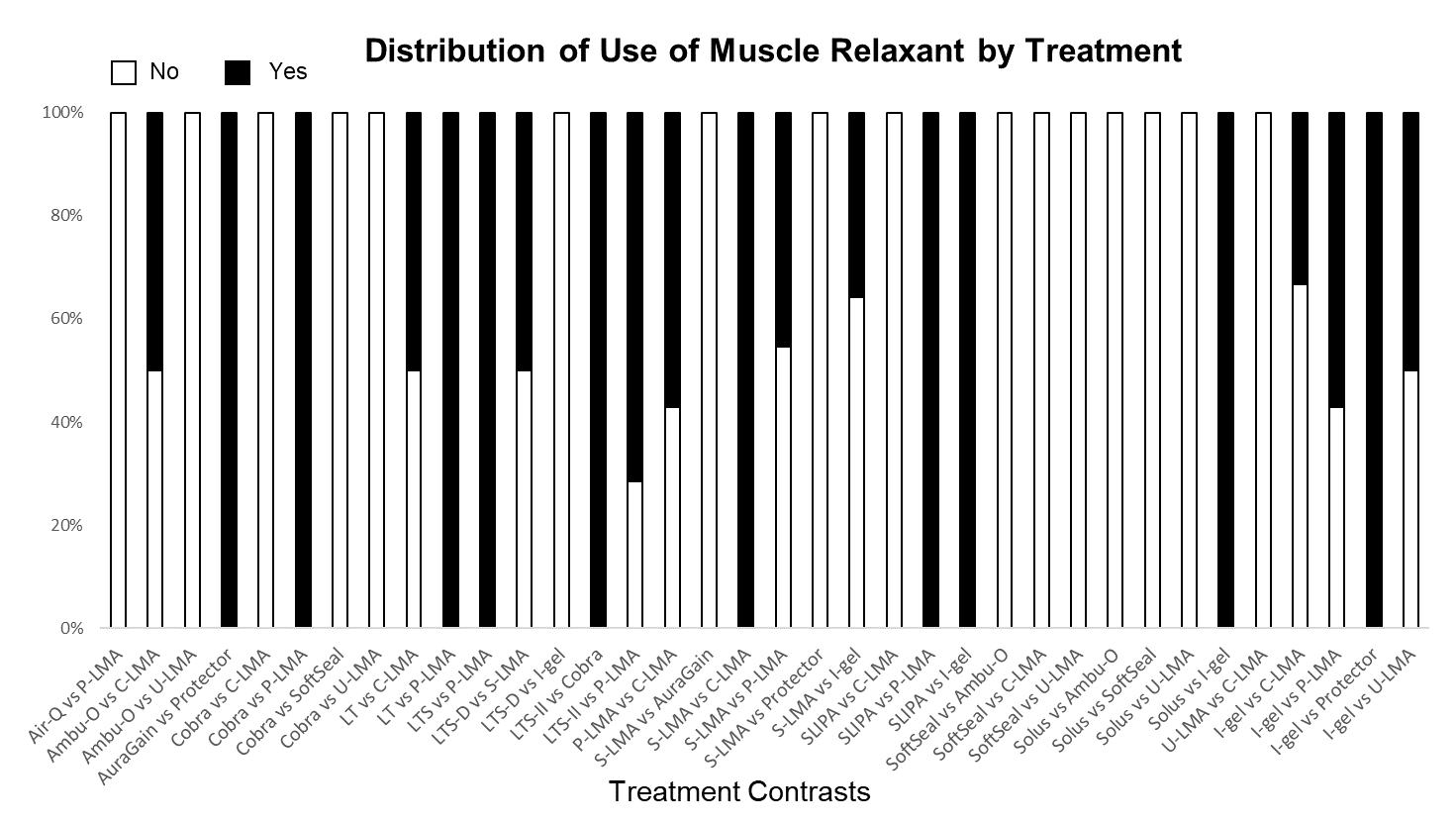
**

**Figure S5: Forest plots of node-splitting model for oropharyngeal leak pressure**

**Figure S6: Forest plots of node-splitting model for risk of first-attempt insertion failure**

**
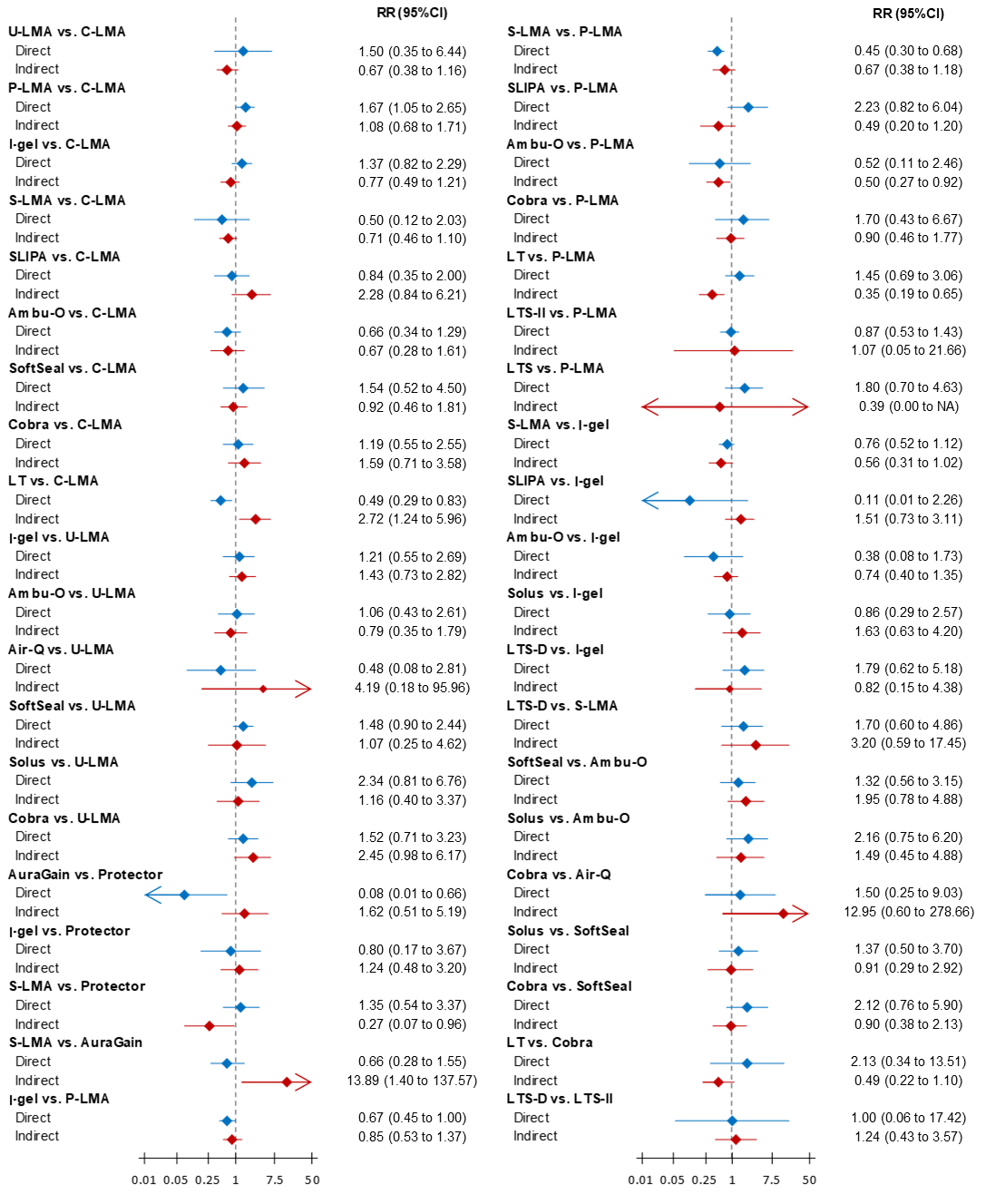
**

**Figure S7: Forest plots of node-splitting model for the postoperative sore throat rate**

**
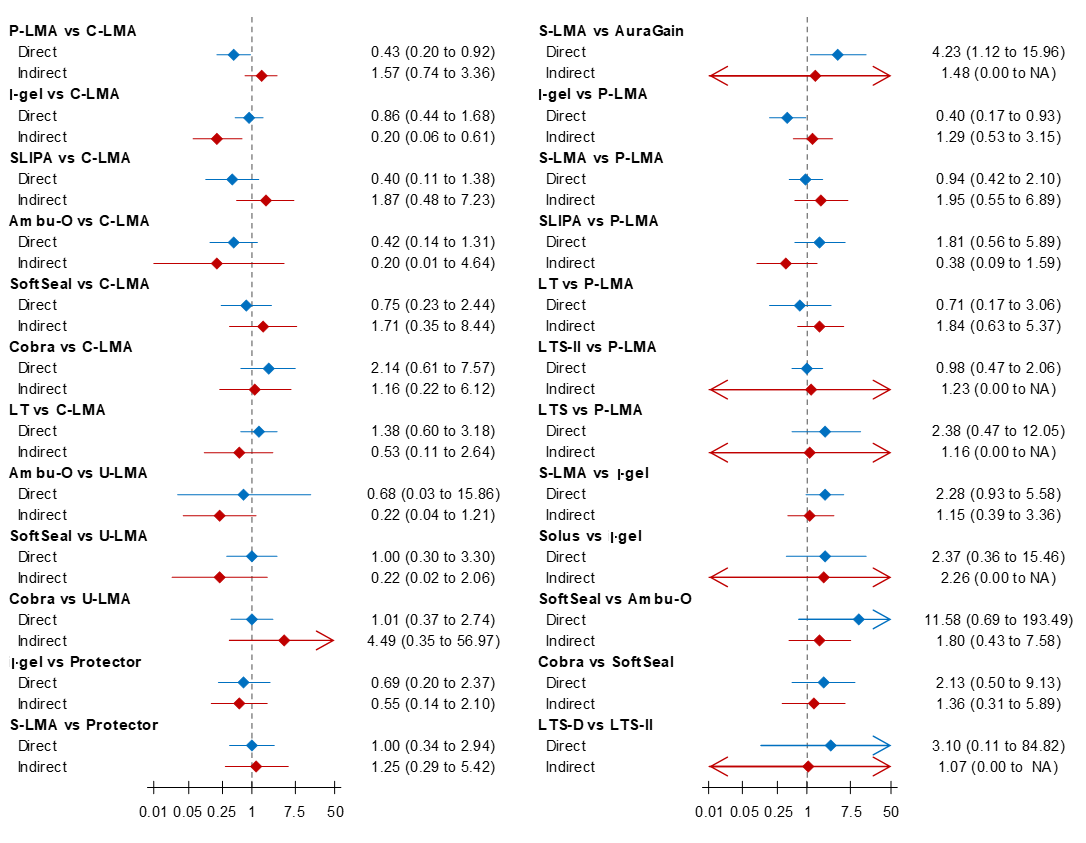
**

**Figure S8: The publication bias assessment of oropharyngeal leak pressure**

A

B

A. Funnel plot

B. Egger test

**Figure S9: The publication bias assessment of** **the risk of first-attempt insertion failure**

A

B

1. Funnel plot
2. Egger test

**Figure S10: The publication bias assessment of the postoperative sore throat rate**


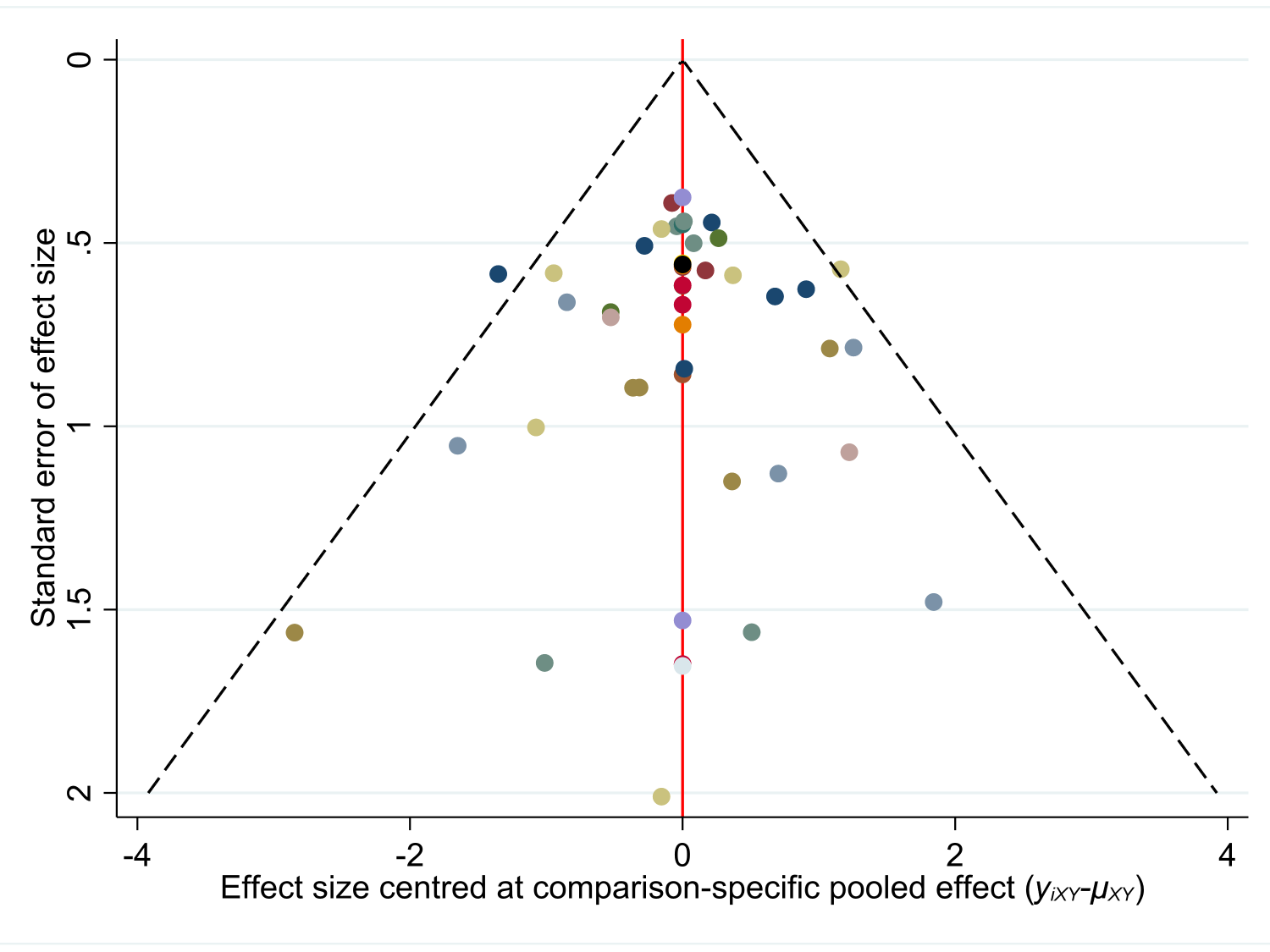


A

B

A. Funnel plot

B. Egger test

**Figure S11: The PRISMA Flow Diagram**


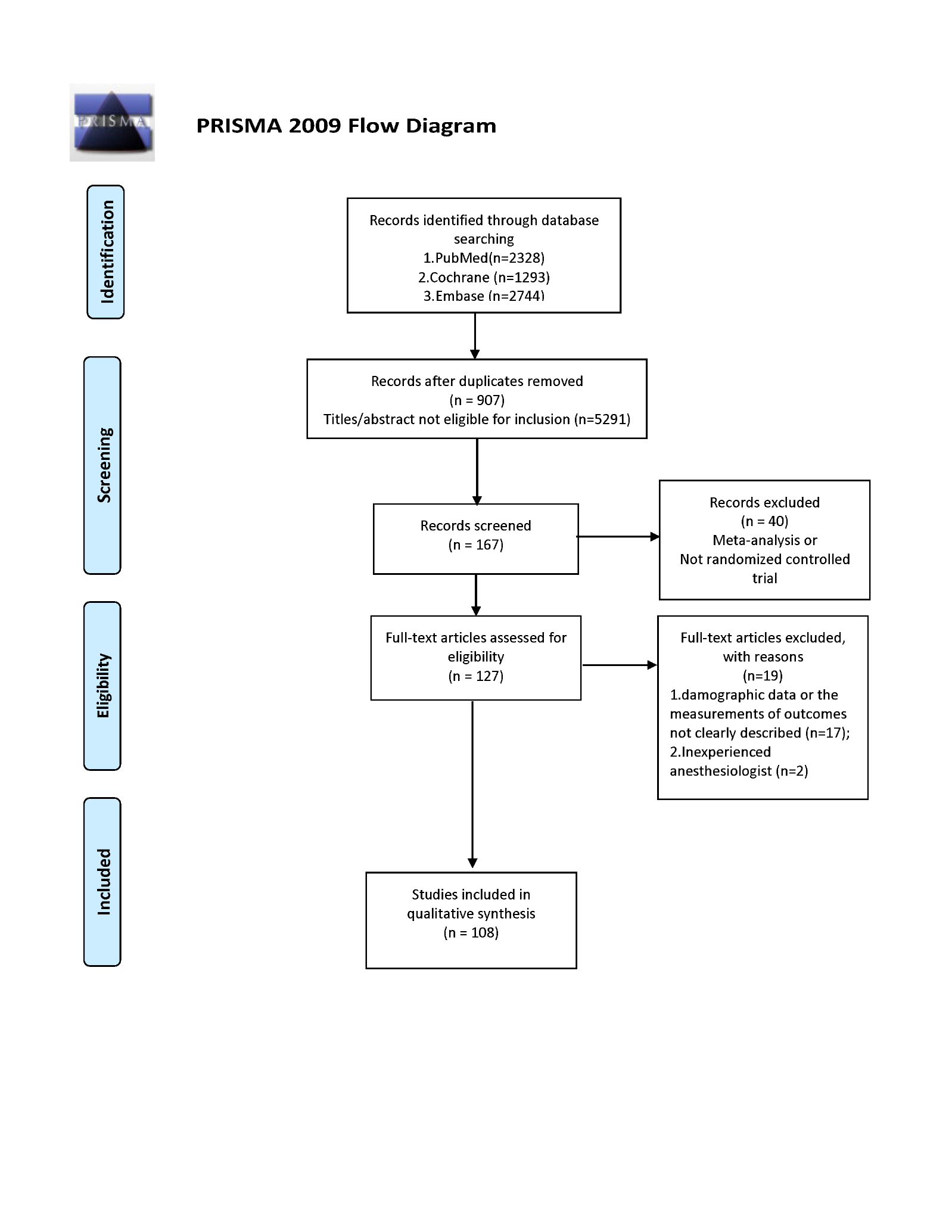

Supplement: Supplementary file 2 — Supplementary Figures. [file 41598_2021_94114_MOESM2_ESM.docx]
